# Pentacyclic aromatic heterocycles from Pd-catalyzed annulation of 1,5-diaryl-1,2,3-triazoles

**DOI:** 10.3762/bjoc.21.194

**Published:** 2025-11-13

**Authors:** Kaylen D Lathrum, Emily M Hanneken, Katelyn R Grzelak, James T Fletcher

**Affiliations:** 1 Department of Chemistry and Biochemistry, Creighton University, 2500 California Avenue, Omaha, NE 68178, USA

**Keywords:** annulation, arenes, antimicrobials, fused-ring systems, UV–vis spectroscopy

## Abstract

A series of pentacyclic aromatic heterocycles representing functionalized phenanthridines, naphthyridines, and phenanthrolines were prepared via Pd-catalyzed annulation of 1,5-diaryl-1,2,3-triazoles. Analogs with triazole N1-connected *ortho*-bromobenzene subunits successfully underwent annulation under microwave irradiation in yields of 31–90%. In contrast, annulations of triazole C1-connected *ortho*-bromobenzene subunit analogs failed under microwave irradiation but were successful using conventional thermal heating in yields of 31–65%. The expanded nature of the aromatic ring system following annulation was reflected by the downfield shifting of aromatic ^1^H NMR signals and the red-shifting of UV–visible absorbance signals relative to their non-annulated counterparts. Structural rigidity of annulated systems compared to non-annulated counterparts was reflected by emission signals with increased intensity and decreased Stokes shifts. Five benzotriazolophenanthroline regioisomers sharing structural similarity regarding N center placement showed antimicrobial activity, as measured by minimum inhibitory concentration assays. MIC values of 2–16 μM towards Gram-positive bacteria *Staphylococcus epidermidis* and *Bacillus subtilis* and 8–16 μM towards *Saccharomyces cerevisiae* yeast were observed for these annulated molecules, while their analogous non-annulated control compounds were not bioactive.

## Introduction

**Figure 1:**
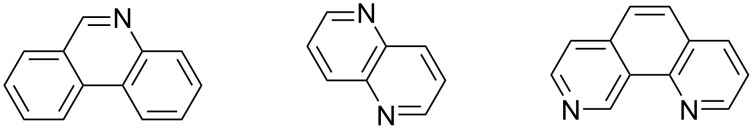
Examples of polycyclic aromatic heterocycle structures: phenanthridine (left), 1,5-naphthyridine (center), and 1,9-phenanthroline (right).

Polycyclic aromatic heterocycles are a diverse class of small molecules with utility in a wide range of applications ranging from materials [1–2] to bioactive molecules [3–4]. Phenanthridine [5–6], naphthyridine [7–9], and phenanthroline [10] ring structures have each been studied for such potential uses (Figure 1). While reported analogs within this class of molecules are numerous, there remains a wide range of chemical space representing these heterocycles that has not yet been explored. Hence, there is an ongoing interest in identifying synthetic strategies to access unreported ring structures and in characterizing the resulting physical and biological properties of such compounds.

The utility of click chemistry [11–12] for achieving chemoselective conjugation in a diversity of chemical environments has established the 1,2,3-triazole ring as a ubiquitous heterocycle in many research areas such as therapeutics [13–16], chemosensors [17–19], bioconjugation [20–21], and materials [22–24]. Numerous examples of 1,4-diaryl-1,2,3-triazoles with quinoline and isoquinoline subunits have been reported, including those with anticancer [25–29], antiviral [30–31], antibacterial [32], antifungal [33], antimalarial [34–35], antitubercular [36], and other bioactive properties [37–44]. In contrast, examples of 1,5-diaryl-1,2,3-triazoles with quinoline or isoquinoline subunits are lacking [45]. The neighboring proximity of the arene subunits in such 1,5-regioisomers allows for potential intramolecular annulation to form expanded ring structures. Recent reports have established this as a valid approach to preparing 1,2,3-triazole-fused phenanthridine analogs [46–47].

**Figure 2:**
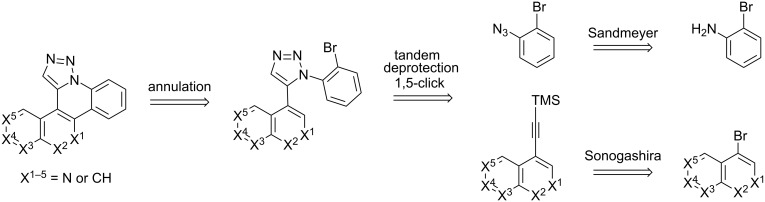
Overview of the synthetic scheme employed by this study.

The goal of this study was to determine whether a modular click/annulation synthetic approach could be successfully used to expand beyond the phenanthridine ring itself to also include benzophenanthridine, dibenzonaphthyridine, and benzophenanthroline heterocycles of previously unreported identity (Figure 2). In addition to studying how naphthalene, quinolone, and isoquinoline incorporation impact click and annulation efficiency, characterizing the physical and biological properties of such polycyclic aromatic heterocycles would serve as an initial evaluation of their potential use in chemical, material, and therapeutic applications.

## Results and Discussion

The two-step approach used to prepare the target pentacyclic aromatic heterocycles **13**–**18** via tandem deprotection/click chemistry followed by Pd-catalyzed annulation is summarized in Table 1. The alkyne-substituted analogs **1**–**6** [48–52] used in this study were prepared from commercially available aryl halides using microwave-promoted Sonogashira coupling (Table S1, Supporting Information File 1). Reaction of each TMS-protected alkyne with *ortho*-bromoazidobenzene produced 1,5-diaryl-1,2,3-triazole products **7**–**12**, each possessing an *ortho-*bromoaryl reactive site necessary for the annulation step.

**Table 1:** Synthesis of pentacyclic aromatic heterocycles from varying alkynes.^a^

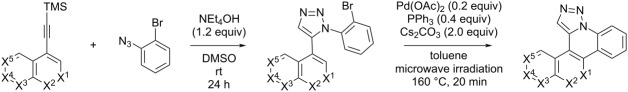		

alkyne^b^	product step 1 (cycloaddition)^c^	product step 2 (annulation)^d^

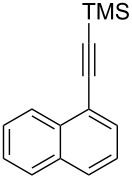 **1**	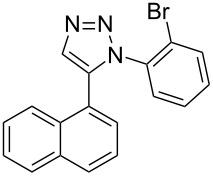 **7** (85%)	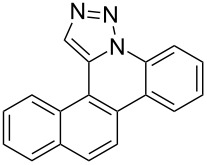 **13** (90%)
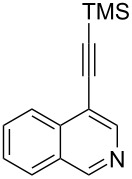 **2**	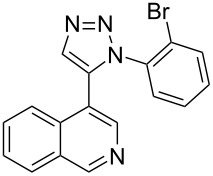 **8** (84%)	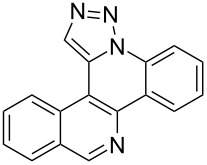 **14** (43%)
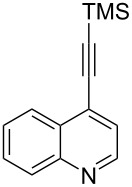 **3**	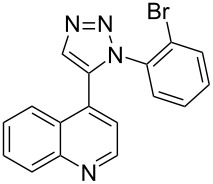 **9** (74%)	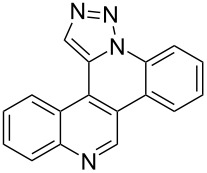 **15** (72%)
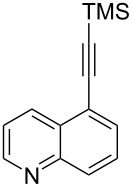 **4**	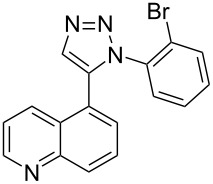 **10** (43%)	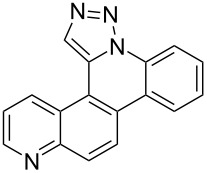 **16** (31%)
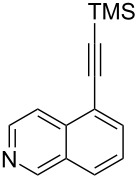 **5**	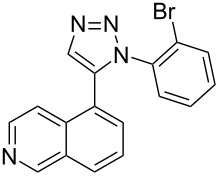 **11** (77%)	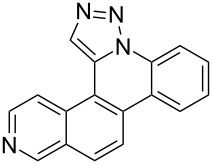 **17** (49%)
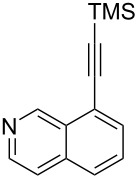 **6**	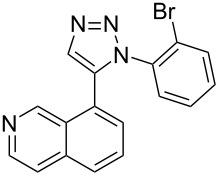 **12** (79%)	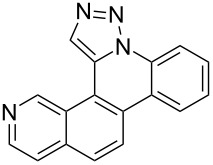 **18** (46%)

^a^Isolated yields shown; ^b^see Supporting Information File 1 for synthetic details; ^c^reactions run at 100 mM concentration; ^d^reactions run at 62.5 mM concentration.

**Figure 3:**
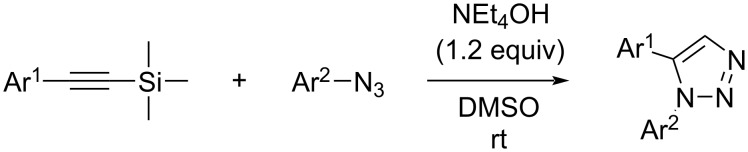
Base-catalyzed [53] tandem deprotection/cycloaddition reaction conditions used to prepare 1,5-diaryl-1,2,3-triazole compounds.

Regioselective formation of 1,5-diaryl-1,2,3-triazoles **7**–**12** was achieved using a modification of the base-catalyzed conditions reported by Kwok [53], where a stoichiometric plus additional catalytic amount of tetraethylammonium hydroxide base in DMSO solvent was used to promote tandem trimethylsilyl deprotection and cycloaddition in one preparation (Figure 3). Isolated yields of this tandem approach preparing **7**–**12** ranged from 43–85%, which were similar to running the deprotection and cycloaddition reactions sequentially.

Pd-catalyzed annulation using a modification of previously reported reaction conditions [46] under microwave irradiation instead of thermal heating converted 1,5-diaryl-1,2,3-triazoles **7**–**12** into target pentacyclic aromatic heterocycles **13**–**18**. Yields of annulation reactions for naphthalene-containing analog **13** (90%) was appreciably higher than for quinoline and isoquinoline derivatives **14**–**18** (31–72%). Due to triazole subunit connectivity to the napththalene, quinolone, or isoquinoline subunit of each analog paired with the *ortho*-bromophenyl group, only a single pentacyclic regioisomer was possible upon intramolecular annulation [46].

A similar two-step approach as that used to prepare **13**–**18** was used to prepare the target pentacyclic aromatic heterocycles **31**–**36** where the attachment of alkyne and azide functional groups was reversed, as summarized in Table 2. The azide analogs **19**–**24** [25,28,31,43] used in this study were prepared from commercially available amines using the Sandmeyer reaction (Table S2, Supporting Information File 1). Reaction of each azide with *ortho*-bromo(trimethylsilylethynyl)benzene produced 1,5-diaryl-1,2,3-triazole products **25**–**30**, each possessing an *ortho-*bromoaryl reactive site necessary for the annulation step. The tandem deprotection/click reaction was used to successfully prepare 1,5-diaryl-1,2,3-triazoles **25**–**30** in yields ranging from 72–83%.

**Table 2:** Synthesis of pentacyclic aromatic heterocycles from varying azides.^a^

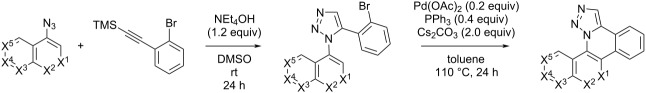		

azide^b^	product step 1 (cycloaddition)^c^	product step 2 (annulation)^d^

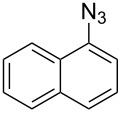 **19**	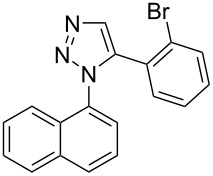 **25** (72%)	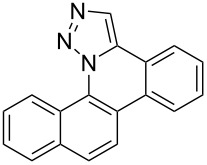 **31** (65%)
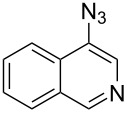 **20**	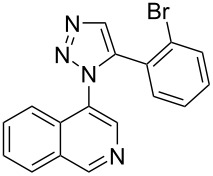 **26** (83%)	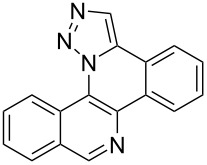 **32** (34%)
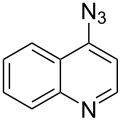 **21**	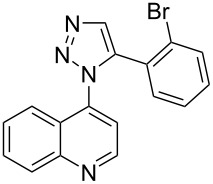 **27** (79%)	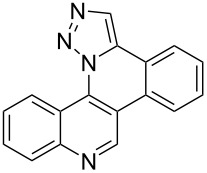 **33** (42%)
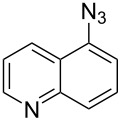 **22**	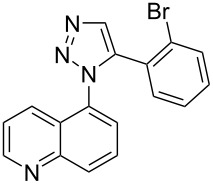 **28** (74%)	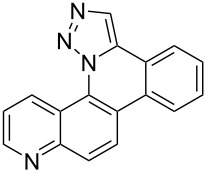 **34** (31%)
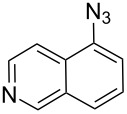 **23**	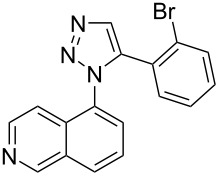 **29** (81%)	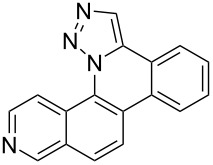 **35** (49%)
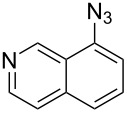 **24**	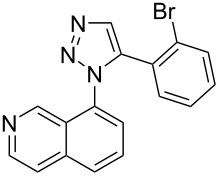 **30** (73%)	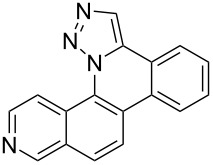 **36** (51%)

^a^Isolated yields shown; ^b^see Supporting Information File 1 for synthetic details; ^c^reactions run at 100 mM concentration; ^d^reactions run at 20 mM concentration.

Interestingly, Pd-catalyzed annulation using the microwave irradiation conditions successful for preparing **13**–**18** where the triazole connectivity of subunits was reversed failed to promote annulation needed to form **31**–**36**. Fortunately, previously reported Pd-catalyzed annulation under thermal heating conditions [46] was successful for preparing these compounds. Yields of annulation reactions for the naphthalene-containing analog **31** (65%) was once again appreciably higher than that of the quinoline and isoquinoline derivatives **32**–**36** (31–51%), but there appeared to be no significant impact on yield due to inverting the 1,2,3-triazole connectivity.

**Figure 4:**
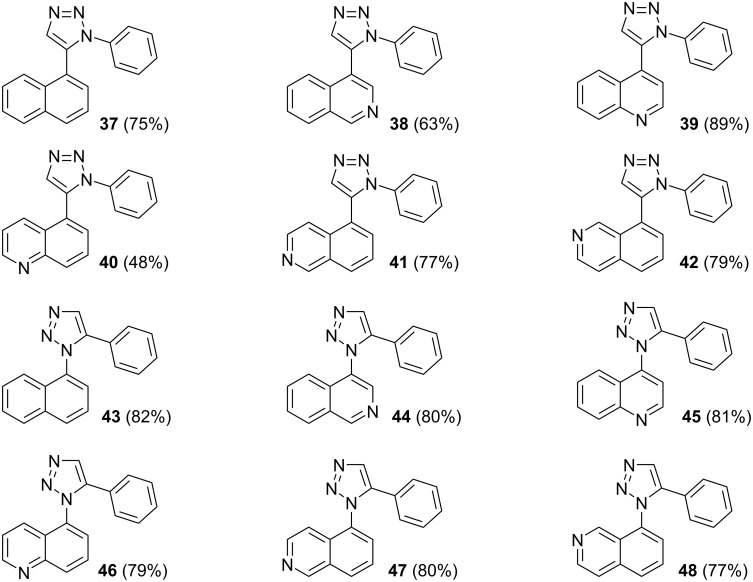
Identity of 1,5-diaryl-1,2,3-triazole control compounds prepared from tandem deprotection/click conditions (**37**–**42**) or standard base-catalyzed [53] click conditions (**43**–**48**) with isolated yields shown.

With the goal of defining the physical and biological properties of these annulated pentacyclic aromatic heterocycles relative to their individual arene components, a “non-annulated” counterpart for each pentacyclic ring system was also prepared using non-brominated azide and alkyne reactants (Figure 4). Although 1,4-disubstituted-1,2,3-triazoles possessing quinoline and isoquinoline subunits are known [25–44], no prior examples of analogous 1,5-regioisomers have been reported. 1,5-Diaryl-1,2,3-triazole control compounds **37**–**42** were prepared via tandem deprotection/click reactions of TMS-protected alkynes with phenyl azide in yields similar to bromophenyl annulation precursors **7**–**12** and **25**–**30**. Compounds **43**–**48**, inverting the diaryltriazole connectivity, were prepared via base-catalyzed click reaction [53] of aryl azides with commercially available phenylacetylene. Due to the free rotation of the single bonds connecting each arene subunit to the bridging triazole ring, these 1,5-diaryl-1,2,3-triazole compounds can adopt significantly different overall molecular shape differences by allowing such subunits to rotate out of coplanarity due to steric strain, diminishing conjugation between subunits.

**Figure 5:**
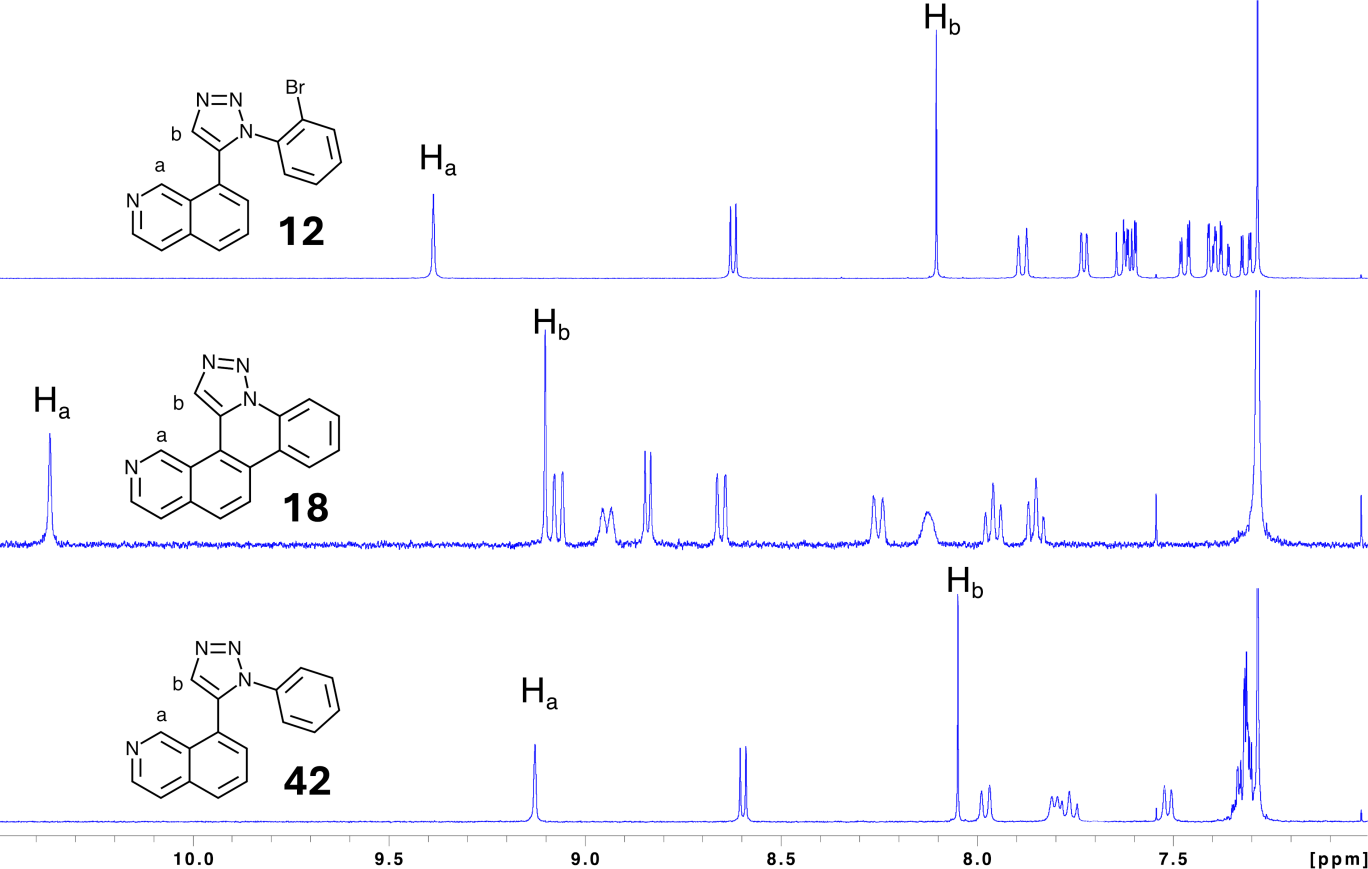
Exemplary comparison of ^1^H NMR aromatic signal shifts for annulated and non-annulated compounds (CDCl_3_ solvent).

Formation of pentacyclic ring systems via annulation led to several expected spectroscopic signatures indicating the formation of an expanded aromatic π-system. ^1^H NMR signals in the aromatic region shift downfield significantly following annulation relative to both the bromoaryl synthetic precursor and control compound. Figure 5 illustrates this general trend by comparing annulated **18** with both its precursor **12** and its non-annulated control **42**. Overall, each signal in the aromatic region shifts between 0.5–1.0 ppm downfield upon annulation. More extensive shifts such as the two singlets corresponding to the hydrogens attached at triazole C4 and isoquinoline C1 ring locations of **18** reflect their more sterically crowded location of neighboring subunits within the pentacyclic ring system. A general downfield shifting of the entire set of signals by no less than 0.5 ppm supports the expansion of the overall aromatic π-system upon annulation. Aromatic signal symmetry for non-annulated compounds **12** and **42** show a lack of distinct rotational isomers at room temperature on the NMR timescale. These trends were observed similarly for the other analogs of this study (see Supporting Information File 1).

**Figure 6:**
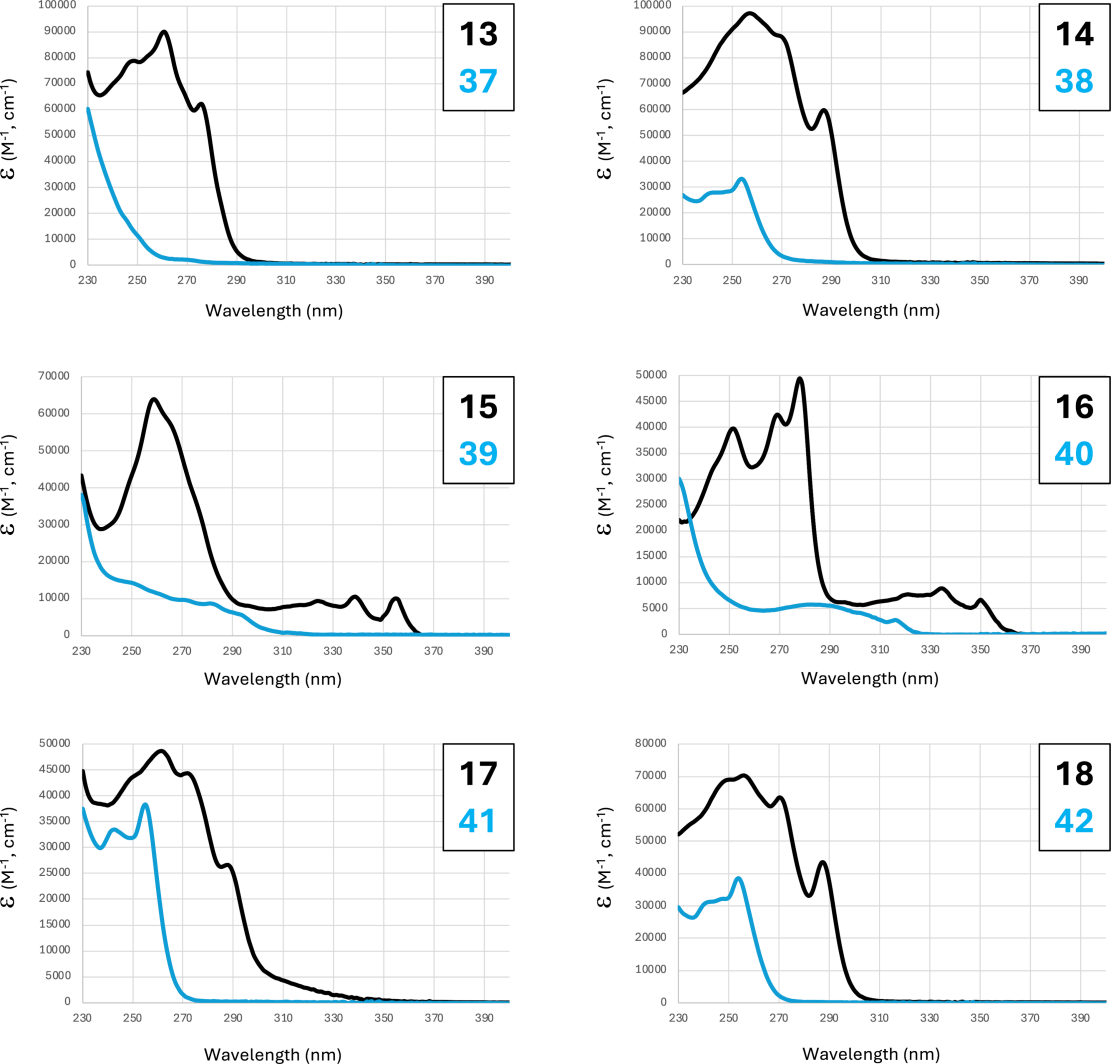
UV–visible absorbance spectra of annulated **13**–**18** (black lines) compared with their non-annulated control compounds **37**–**42** (blue lines) in acetonitrile solvent.

Optoelectronic properties of each annulated product and its respective control compound were examined via UV–visible absorption and emission spectroscopy. Figure 6 shows the general red-shifting of UV absorbance signals for the annulated pentacycles presented in Table 1 relative to their non-annulated counterparts, further indicating an aromatic π-system expansion upon annulation. Similar trends were observed for those compounds shown in Table 2 (Figure S1, Supporting Information File 1).

Table 3 summarizes the observed λ_max_ values for absorption and emission bands for each compound in this study. Due to their structural rigidity, annulated compounds comprised of quinoline or isoquinoline subunits generated emission signals with greater intensity and smaller Stokes shifts compared to their rotationally flexible non-annulated counterparts (Supporting Information File 1, Figures S2 and S3). Non-annulated compounds with naphthalene subunits largely reflect the emission properties of their individual naphthalene subunit. Non-annulated compounds with quinoline or isoquinoline subunits connected at the triazole C5 position were non-emissive under the conditions utilized, while the majority of the N1-connected analogs showed weak but observable signals. Such influence of triazole connectivity on the emission intensity of attached arenes has been previously reported [54]. Within the pentacyclic series itself, minor differences in emission energy were observed as the naphthalene, quinolone, and isoquinoline subunits were varied, and compounds **31**–**36** with N1-triazole subunit connectivity displayed generally sharper emission signals than their C5-triazole subunit connected counterparts **13**–**18**.

**Table 3:** Summary of UV–vis absorbance/emission signals.

compound	absorbance (nm)	emission (nm)^a^

annulated ring analogs		

**13**	249, 261, 276	368
**14**	257, 270 (sh), 287	373 (sh), 385
**15**	259, 266 (sh), 324, 339, 355	365, 380
**16**	251, 269, 278, 321, 334, 350	387
**17**	251 (sh), 261, 272, 289	372, 387
**18**	251, 256, 270, 287	372, 385
**31**	250 (sh), 257, 272, 282, 295 (sh), 326, 341, 353	357, 374, 394, 410
**32**	246, 254, 265 (sh), 277, 301, 324, 340, 356	369, 384, 402 (sh)
**33**	248 (sh), 255, 270, 281, 338, 354	364, 379, 398 (sh)
**34**	250 (sh), 256, 271, 281, 322, 336, 352	370 (sh), 381
**35**	250 (sh), 257, 278, 328	379, 395
**36**	248 (sh), 254, 268, 278, 323, 339, 356	366, 382, 400 (sh)

non-annulated diaryl control analogs		

**37**	<230, 270 (sh)	377
**38**	243, 255	n.s.
**39**	270, 281	n.s.
**40**	<230, 286, 316	n.s.
**41**	<230, 244, 255	n.s.
**42**	242 (sh), 248, 254	n.s.
**43**	<230, 270, 281	385
**44**	<230, 308, 320	435
**45**	231, 303, 317	n.s.
**46**	<230, 248 (sh), 302, 315	n.s.
**47**	<230, 309, 322	422
**48**	<230, 308, 314, 320	417

^a^10 μM solutions in CH_3_CN solvent, excitation λ = λ_max_ of each compound 230–300 nm; sh = shoulder; n.s. = no signal.

Because fused ring heterocycles are common components of bioactive molecules, a preliminary evaluation of toxicity for this newly defined class of compounds was completed. Antimicrobial potency against Gram-positive bacteria *Bacillus subtilis* and *Staphylococcus epidermidis*, Gram-negative bacteria *Escherichia coli* and *Klebsiella aerogenes,* and yeast *Candida albicans* and *Saccharomyces cerevisiae* was determined using minimum inhibitory concentration (MIC) assays [55–56]. As summarized in Table 4, only five of the twenty-four compounds tested displayed an ability to suppress microbial growth under the conditions utilized. The observed MIC values of these compounds against Gram-positive bacteria and yeast were similar to benzalkonium chloride, a common commercial disinfectant.

**Table 4:** Minimum inhibitory concentration assay results.^a^

compound	antimicrobial potency (μM)					
	Gram(+) bacteria^b^		Gram(−) bacteria^b^		yeast^c^	
	*B. subtilis*	*S. epidermidis*	*E. coli.*	*K. aerogenes*	*C. albicans*	*S. cerevisiae*

**13**–**16**, **31**–**33**, **37**–**48**	>250	>250	>250	>250	>250	>250
**17**	16	16	>250	>250	62	16
**18**	8	8	>250	>250	250	8
**34**	8	8	>250	>250	>250	>250
**35**	8	4	>250	>250	>250	16
**36**	4	2	>250	>250	>250	8
BAC^d^	8	8	125	125	62	8

^a^See Supporting Information File 1 for experimental details; ^b^Mueller–Hinton broth growth media used; ^c^YM broth growth media used; ^d^benzalkonium chloride.

Interestingly, while each of the twelve annulated compounds shared the same rigid isosteric pentacyclic ring orientation, the distribution of nitrogen atom centers within the ring system appears essential for eliciting bioactivity. Orientation of isoquinoline and N3-triazole subunit nitrogen atoms is identical between **17** and **35** as well as between **18** and **36**. In contrast, **34** showed toxicity towards Gram-positive bacteria while its triazole-connected counterpart **16** did not. Removal of the non-triazole N center (**13** and **31**) or its relocation elsewhere (**14**–**16** and **32**–**33**) results in a total loss of bioactivity against all organisms under the concentrations studied. None of the non-annulated control compounds **37**–**48** were measurably bioactive, including those serving as controls for the five bioactive derivatives, highlighting the additional importance of structural rigidity on bioactivity within this series. Elucidation of the mechanism of action for these bioactive pentacyclic compounds will be the focus of future investigation.

## Conclusion

A series of previously unreported pentacyclic aromatic heterocycles representing expanded phenanthridine, naphthyridine, and phenanthroline ring systems were prepared via Pd-catalyzed annulation reactions of 1,5-diaryl-1,2,3-triazoles with varying naphthalene, quinolone, and isoquinoline subunits. Heterocycle subunit identity and triazole C/N connectivity influenced the annulation reaction efficiency. Aromatic π-system expansion resulting from annulation was characterized by NMR, absorption and emission spectroscopy. Five benzotriazolophenanthroline regioisomers sharing structural similarity showed significant antimicrobial potency towards Gram-positive bacteria and yeast relative to their non-annulated control analogs as well as to the other annulated pentacycles in this study, warranting a future investigation into their possible mechanism of action. Studies focusing on the reactivity of this family of pentacyclic aromatic heterocycles towards N-benzylation and the antimicrobial properties of such resulting quaternary ammonium compounds are ongoing.

## Supporting Information

File 1 Description of materials, experimental methods, synthetic procedures, analytical characterization and copies of NMR spectra for novel compounds.

## Data Availability

All data that supports the findings of this study is available in the published article and/or the supporting information of this article.
