# Supplementary material for: Pentacyclic aromatic heterocycles from Pd-catalyzed annulation of 1,5-diaryl-1,2,3-triazoles
Source: Beilstein J Org Chem. 2025 Nov 13;21:2524–34. doi: 10.3762/bjoc.21.194 (PMC12621637; doi:10.3762/bjoc.21.194)
Supplement: File 1 — Description of materials, experimental methods, synthetic procedures, analytical characterization and copies of NMR spectra for novel compounds. [file Beilstein_J_Org_Chem-21-2524-s001.pdf]

BEILSTEIN JOURNAL OF ORGANIC CHEMISTRY

Supporting Information

for

Pentacyclic aromatic heterocycles from Pd-catalyzed annulation of 1,5-diaryl-1,2,3-triazoles

Kaylen D. Lathrum, Emily M. Hanneken, Katelyn R. Grzelak and James T. Fletcher

Beilstein J. Org. Chem. **2025,** *21,* 2524–2534. doi:10.3762/bjoc.21.194

Description of materials, experimental methods, synthetic procedures, analytical characterization and copies of NMR spectra for novel compounds

License and Terms: This is a supporting information file under the terms of the Creative Commons Attribution License (https://creativecommons.org/licenses/by/4.0). Please note that the reuse, redistribution and reproduction in particular requires that the author(s) and source are credited and that individual graphics may be subject to special legal provisions.
The license is subject to the *Beilstein Journal of Organic Chemistry* terms and conditions: (https://www.beilstein-journals.org/bjoc/terms)

Table of contents

S3	Experimental methods, description of materials
S4-6	General synthetic procedures
S7	Antimicrobial assay procedure
S8	**Table S1.** Preparation of alkyne reactants
S9	**Table S2.** Preparation of azide reactants
S10	**Figure S1.** Absorbance spectra of **31**–**36** and **43**–**48**
S11	**Figure S2.** Emission spectra of **13**–**18** and **37**–**42**
S12	**Figure S3.** Emission spectra of **31**–**36** and **43**–**48**
S13-S27	Preparation and characterization of **7**–**18, 25**–**48**
S28	^{1}H and ^{13}C NMR characterization of **7**
S29	^{1}H and ^{13}C NMR characterization of **8**
S30	^{1}H and ^{13}C NMR characterization of **9**
S31	^{1}H and ^{13}C NMR characterization of **10**
S32	^{1}H and ^{13}C NMR characterization of **11**
S33	^{1}H and ^{13}C NMR characterization of **12**
S34	^{1}H and ^{13}C NMR characterization of **13**
S35	^{1}H and ^{13}C NMR characterization of **14**
S36	^{1}H and ^{13}C NMR characterization of **15**
S37	^{1}H and ^{13}C NMR characterization of **16**
S38	^{1}H and ^{13}C NMR characterization of **17**
S39	^{1}H and ^{13}C NMR characterization of **18**
S40	^{1}H and ^{13}C NMR characterization of **25**
S41	^{1}H and ^{13}C NMR characterization of **26**
S42	^{1}H and ^{13}C NMR characterization of **27**
S43	^{1}H and ^{13}C NMR characterization of **28**
S44	^{1}H and ^{13}C NMR characterization of **29**
S45	^{1}H and ^{13}C NMR characterization of **30**
S46	^{1}H and ^{13}C NMR characterization of **31**
S47	^{1}H and ^{13}C NMR characterization of **32**
S48	^{1}H and ^{13}C NMR characterization of **33**
S49	^{1}H and ^{13}C NMR characterization of **34**
S50	^{1}H and ^{13}C NMR characterization of **35**
S51	^{1}H and ^{13}C NMR characterization of **36**
S52	^{1}H and ^{13}C NMR characterization of **37**
S53	^{1}H and ^{13}C NMR characterization of **38**
S54	^{1}H and ^{13}C NMR characterization of **39**
S55	^{1}H and ^{13}C NMR characterization of **40**
S56	^{1}H and ^{13}C NMR characterization of **41**
S57	^{1}H and ^{13}C NMR characterization of **42**
S58	^{1}H and ^{13}C NMR characterization of **43**
S59	^{1}H and ^{13}C NMR characterization of **44**
S60	^{1}H and ^{13}C NMR characterization of **45**
S61	^{1}H and ^{13}C NMR characterization of **46**
S62	^{1}H and ^{13}C NMR characterization of **47**
S63	^{1}H and ^{13}C NMR characterization of **48**
S64-S65	References

Experimental methods

NMR analyses were obtained on a 400 MHz Bruker Ascend spectrometer. HRMS analyses were acquired on a Bruker micrOTOF-Q III system using an elution of 0.1% formic acid in acetonitrile. UV–visible absorbance measurements were acquired on an Agilent 8453 spectrophotometer, and data are reported as λ_{max} = nm (log ε). UV–visible emission measurements were acquired on a Varian Cary Eclipse fluorescence spectrophotometer. Melting points were acquired using a Stanford Research Systems Optimelt apparatus. Reactions utilizing microwave heating were run using a CEM Discover SP microwave reactor.

Materials

All chemical reactants and solvents were used as purchased without further purification. Characterization of prepared 1-trimethylsilylethynylnaphthalene(**1**)[1], 4-trimethylsilylethynylquinoline(**2**) [2], 4-trimethylsilylethynylisoquinoline(**3**) [3], 5-trimethylsilylethynylquinoline (**4**) [4], 5-trimethylsilylethynylisoquinoline (**5**) [5], 8-trimethylsilylethynylisoquinoline (**6**) [6], 1-azidonaphthalene (**19**) [7], 4-azidoquinoline (**20**) [8], 4-azidoisoquinoline (**21**) [9], 5-azidoquinoline (**22**) [10], 5-azidoisoquinoline (**23**) [11], 2-bromoazidobenzene [12] and 1-bromo-2-trimethylsilylethynylbenzene [13] resembled that previously described.

Reaction products were purified using an ISCO CombiFlash Nextgen 100 system eluted with a gradient of methylene chloride and ethyl acetate (or 4:1 ethyl acetate/methanol when necessary) using pre-packed 12 g silica columns. Microorganisms used in bioassays were prepared from freeze-dried samples purchased from ATCC (*Bacillus subtilus* (ATCC 6051), *Staphylococcus epidermidis* (ATCC 14990), *Escherichia coli* (ATCC 25922), *Klebsiella aerogenes* (ATCC 13048),

Candida albicans (ATCC 90028), *Saccharomyces cerevisiae* (ATCC 9763)). Mueller–Hinton broth (for bacteria) and YM broth (for yeast) were purchased from Fisher Scientific and prepared as instructed.

Sonogashira coupling reaction

Aryl bromide (10 mmol), trimethylsilylacetylene (11 mmol), $Pd(PPh_3)_4$ 0.05 mmol, CuI (0.1 mmol), triethylamine (8 mL) and acetonitrile (8 mL) were added to a 35 mL size microwave reaction tube after purging solids and liquids separately with argon gas before combining. Reaction tube was sealed to pressurize and heated at 120 °C for 30 minutes using microwave irradiation. After cooling reactions were extracted between methylene chloride and 1.0 M ammonium hydroxide (aq). Organic layer was separated and dried using magnesium sulfate. Drying agent was removed using gravity filtration and solvent was removed via rotary evaporation. The resulting residue was purified using silica gel flash chromatography eluted with methylene chloride.

Sandmeyer azide substitution reaction

Aryl amine (10 mmol) was added to 20 mL water and 5 mL 12 M HCl and then cooled to 0 °C. Sodium nitrite (12.5 mmol) was dissolved in 20 mL water and added to the aryl amine solution dropwise with stirring over 10 minutes. Sodium azide (11.25 mmol) was dissolved in 20 mL water and added to the reaction solution dropwise with stirring over 10 minutes, evolving nitrogen gas bubbles. The reaction was slowly allowed to warm to room temperature by allowing the ice bath to melt and stirred for 20 h. The reaction mixture was extracted with methylene chloride, and the organic layer was separated and dried using magnesium sulfate. Drying agent was removed using gravity filtration and solvent was removed via rotary evaporation. The

resulting residue was purified using silica gel flash chromatography eluted with methylene chloride.

Base-catalyzed tandem deprotection/click reaction

TMS-protected alkyne (1.0 mmol) and azide (1.0 mmol) reactants were dissolved in 10 mL DMSO and with stirring an aqueous solution of 40% tetraethylammonium hydroxide (1.2 mmol) was added. After stirring 24 h at room temperature, reaction was diluted into ethyl acetate and washed successively with aqueous ammonium chloride (1 M) and two portions of deionized water, using saturated aqueous sodium chloride to break emulsions as needed. Organic layer was separated and dried using magnesium sulfate. Drying agent was removed using gravity filtration and solvent was removed via rotary evaporation. The resulting residue was purified using automated silica gel flash chromatography.

Base-catalyzed click reaction

Phenylacetylene (1.0 mmol) and azide (1.0 mmol) reactants were dissolved in 10 mL DMSO and with stirring an aqueous solution of 40% tetraethylammonium hydroxide (0.2 mmol) was added. After stirring 24 h at room temperature, reaction was diluted into ethyl acetate and washed successively with aqueous ammonium chloride (1 M) and two portions of deionized water, using saturated aqueous sodium chloride to break emulsions as needed. Organic layer was separated and dried using magnesium sulfate. Drying agent was removed using gravity filtration and solvent was removed via rotary evaporation. The resulting residue was purified using automated silica gel flash chromatography.

Annulation reaction, thermal conditions

1,5-Diaryl-1,2,3-triazole reactant (0.2 mmol), palladium(II) acetate (0.04 mmol), triphenylphosphine (0.08 mmol) and cesium carbonate (0.4 mmol) were added to a 50 mL reaction tube and deoxygenated using an argon flow. Toluene (10 mL) was deoxygenated using an argon flow in a separate reaction tube and was combined with reactants via cannula transfer. The reaction was kept under positive argon pressure and heated to 110 °C for 24 hours. After cooling to room temperature, reaction contents were extracted between methylene chloride and 5% aqueous ammonium hydroxide. Organic layer was separated and dried using magnesium sulfate. Drying agent was removed using gravity filtration and solvent was removed via rotary evaporation. The resulting residue was purified using automated silica gel flash chromatography.

Annulation reaction, microwave conditions

1,5-Diaryl-1,2,3-triazole reactant (0.2 mmol), palladium(II) acetate (0.04 mmol), triphenylphosphine (0.08 mmol) and cesium carbonate (0.4 mmol) were added to a 10 mL microwave reaction tube and deoxygenated using an argon flow. Toluene (3 mL) was deoxygenated using an argon flow in a separate reaction tube and was combined with reactants via cannula transfer. The reaction was microwave-irradiated to maintain a temperature of 160 °C for 20 minutes while sealed under pressure. After completion of the microwave cycle, reaction contents were extracted between methylene chloride and 5% aqueous ammonium hydroxide. Organic layer was separated and dried using magnesium sulfate. Drying agent was removed using gravity filtration and solvent was removed via rotary evaporation. The resulting residue was purified using automated silica gel flash chromatography.

MIC assay

Inoculum were prepared following standard microdilution assay procedures [14,15] using Mueller–Hinton broth for all bacteria and YM broth for yeast. Each compound was prepared as a 10 mM solution in DMSO. 10 µL of each DMSO stock solution was diluted into 190 µL broth and a 1:1 serial dilution was performed in a 96-well plate. Addition of 100 µL inoculum to each well resulted in a range of 250, 120, 62, 31, 16, 8, 4 and 2 µM concentrations for each MIC assay. Plates were incubated for 20 h (bacteria) and 24 h (yeast) at 37 °C then examined by eye for cloudiness indicating microbial growth. The most dilute member within a serial dilution that remained transparent after 24 h was defined as the minimum inhibitory concentration (MIC) value for that compound/organism combination. Assays were performed in triplicate. Benzalkonium chloride was used as an internal control for each assay plate.

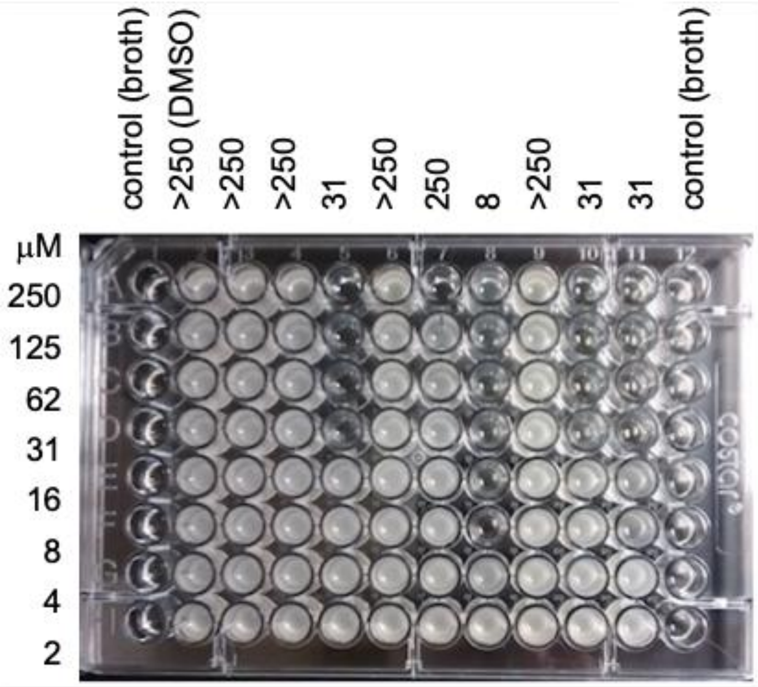

Example of assay plate with *E. coli* growth. Concentration gradient from serial dilution shown on left side, MIC values identified from eye read for growth cloudiness shown above each column.

Table S1: Synthesis of alkyne reactants[a]

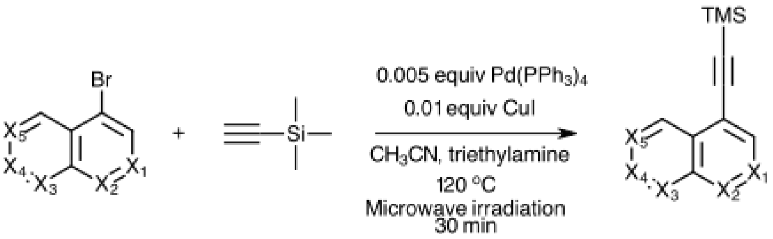

Aryl halide	Product step 1 (cycloaddition)
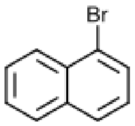	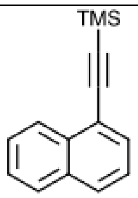 **1** (91%)
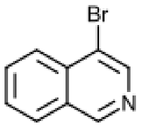	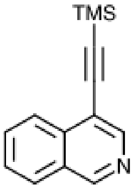 **2** (85%)
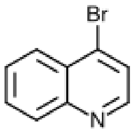	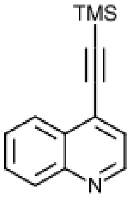 **3** (67%)
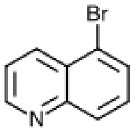	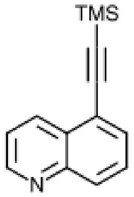 **4** (48%)
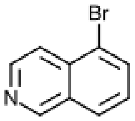	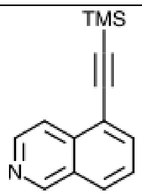 **5** (60%)
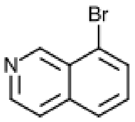	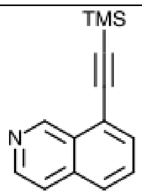 **6** (88%)

[a]Isolated yields shown

Table S2: Synthesis of azide reactants[a]

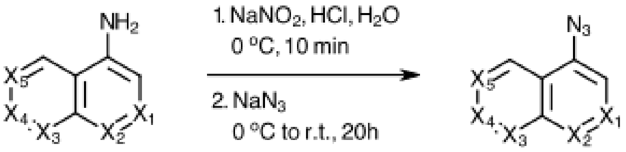

Aryl Halide	Product Step 1 (Cycloaddition)
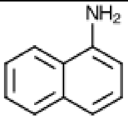	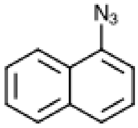 **19** (94%)
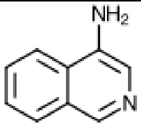	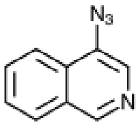 **20** (90%)
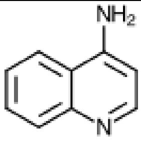	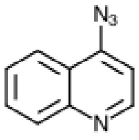 **21** (55%)
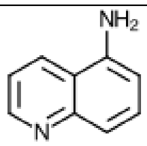	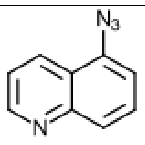 **22** (82%)
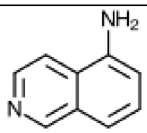	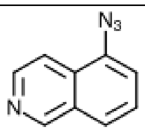 **23** (77%)
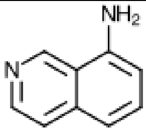	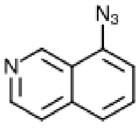 **24** (32%)

[a]Isolated yields shown.

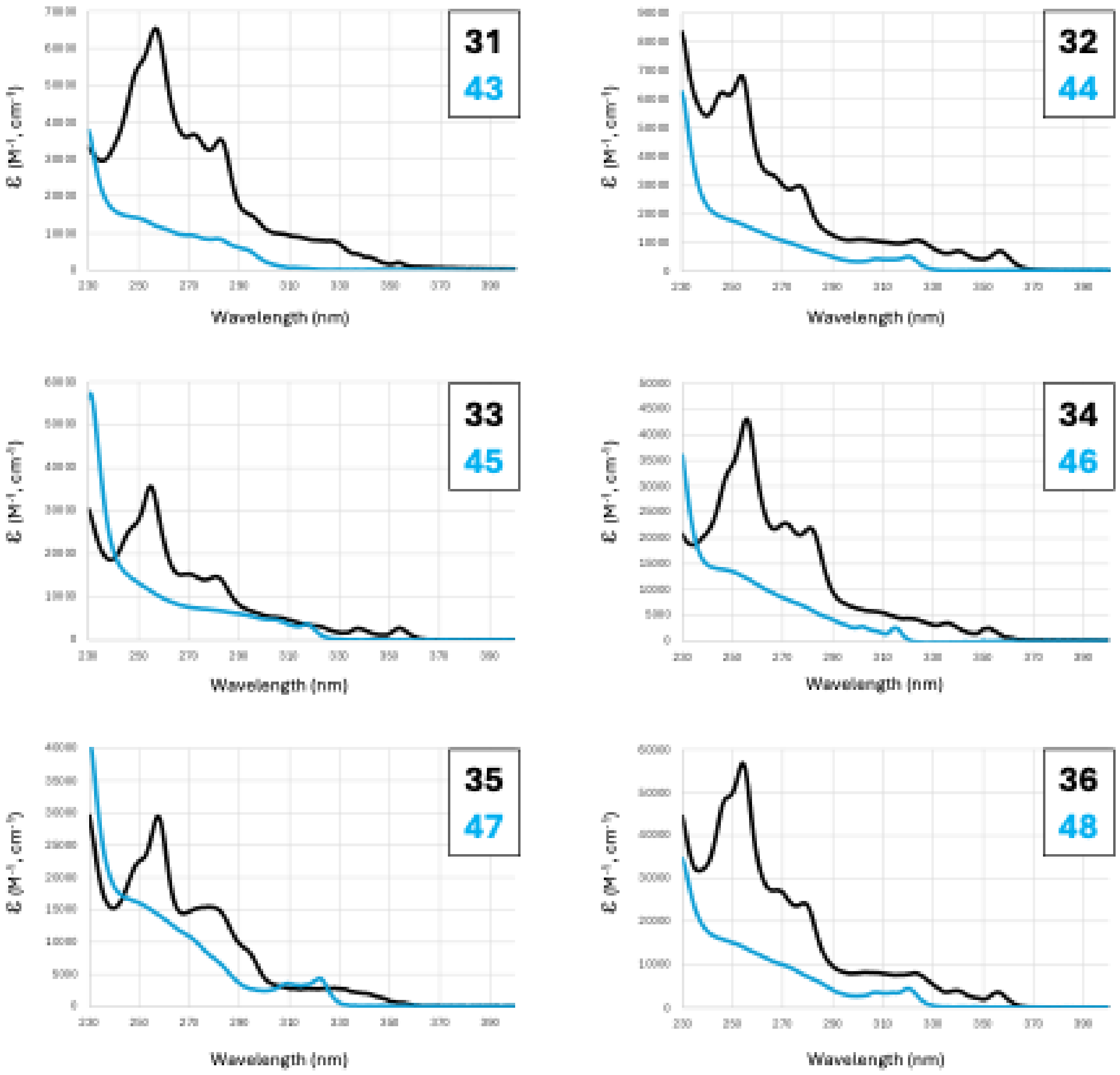

Figure S1. UV–visible absorbance spectra of annulated **31**–**36** (black lines) compared with their non-annulated control compounds **43**–**48** (blue lines) in acetonitrile solvent.

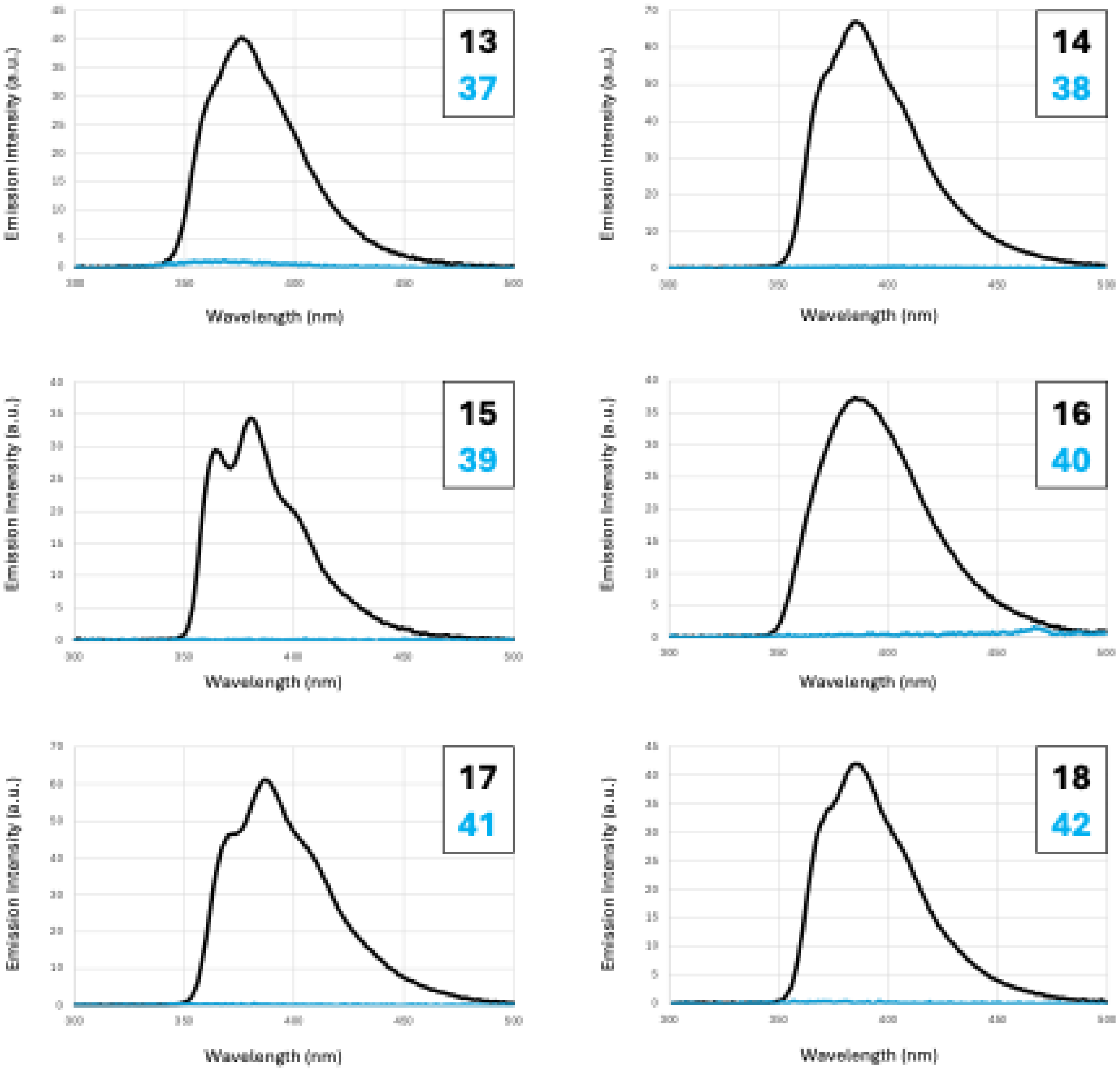

Figure S2. Emission spectra of annulated **13–18** (black lines) compared with their non-annulated control compounds **37–42** (blue lines), 1 mM in acetonitrile solvent. Excitation $\lambda = \lambda_{max}$ of each compound 230–300 nm.

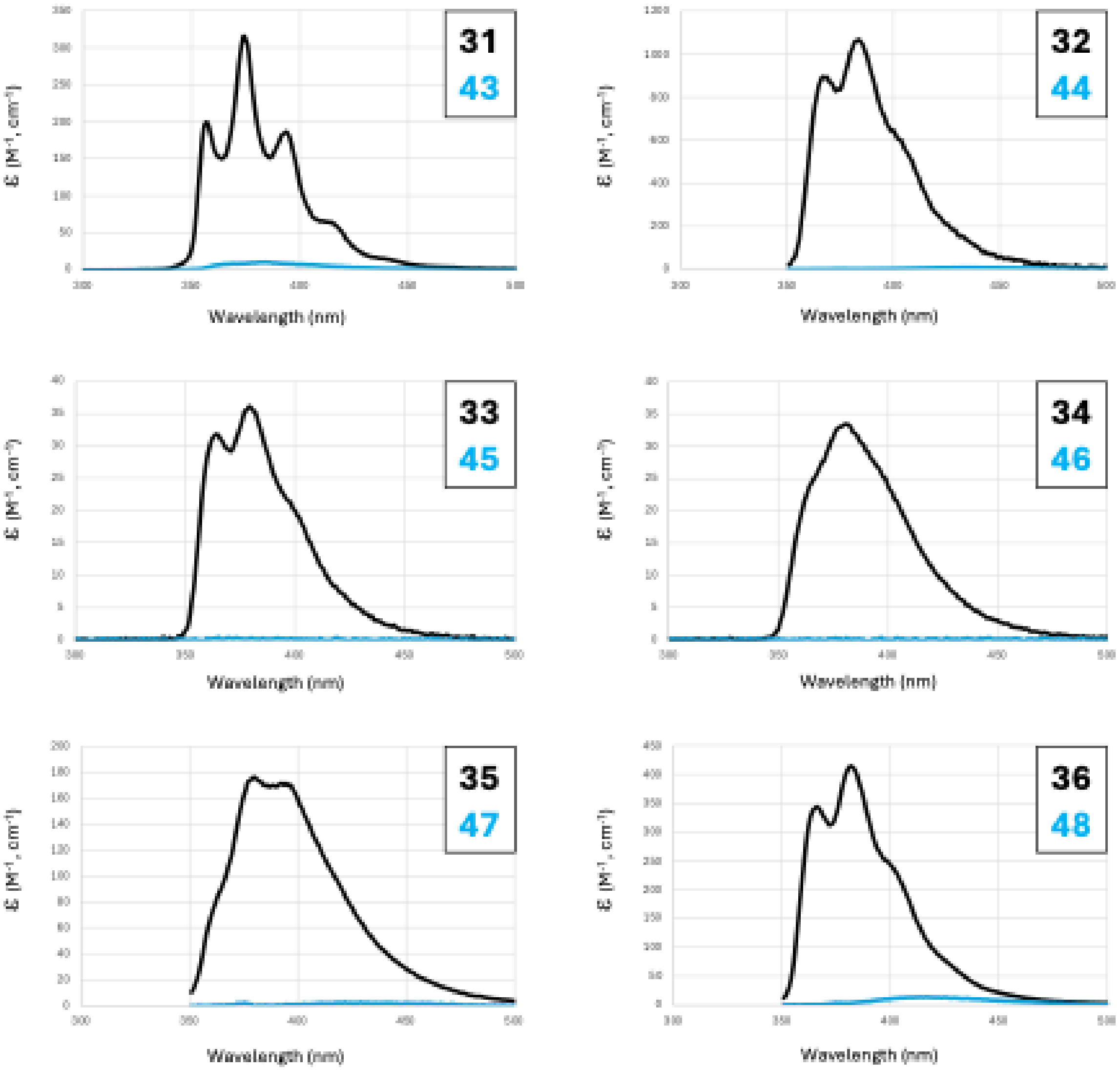

Figure S3. Emission spectra of annulated **31–36** (black lines) compared with their non-annulated control compounds **43–48** (blue lines), 1 mM in acetonitrile solvent. Excitation $\lambda = \lambda_{max}$ of each compound 230–300 nm.

Compound preparation and characterization

1-(2-Bromophenyl)-5-(1-naphthalenyl)-1*H*-1,2,3-triazole (**7**)

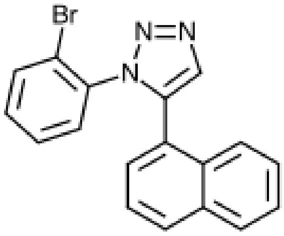

Prepared via base-catalyzed tandem deprotection/click reaction. Orange solid, 85% yield, mp 106-109 °C; ^{1}H NMR (400 MHz, $CDCl_3$): δ 8.04 (s, 1H), 7.92 (m, 1H), 7.88 (m, 2H), 7.61 (m, 1H), 7.55 (m, 2H), 7.37 (m, 2H), 7.29 (3H); ^{13}C NMR (400 MHz, $CDCl_3$): δ 137.4, 136.0, 134.7, 133.9, 133.7, 131.9, 131.5, 130.3, 129.5, 128.8, 128.7, 128.2, 127.3, 126.7, 125.1, 125.0, 123.9, 121.8; HRMS (ESI) *m/z*: Calcd for $C_{18}H_{13}BrN_3^+$ [M+H]$^+$ 350.0287, found 350.0283.

1-(2-Bromophenyl)-5-(4-isoquinolinyl)-1*H*-1,2,3-triazole (**8**)

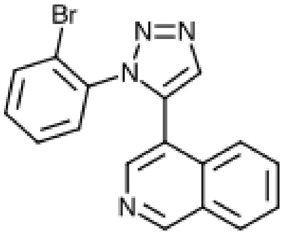

Prepared via base-catalyzed tandem deprotection/click reaction. Orange solid, 84% yield, mp 150-152 °C; ^{1}H NMR (400 MHz, $CDCl_3$): δ 9.25 (s, 1H), 8.31 (s, 1H), 8.11 (s, 1H), 8.06 (d, *J*= 8.1 Hz, 1H), 7.96 (d, *J*= 8.4 Hz, 1H), 7.80 (m, 1H), 7.71 (m, 1H), 7.6 (m, 1H), 7.45 (m, 1H), 7.37 (m, 1H), 7.31 (m, 1H); ^{13}C NMR (400 MHz, $CDCl_3$): δ 154.2, 144.1, 135.6, 135.3, 134.5, 134.4, 134.0, 131.9, 131.8, 129.6, 128.50, 128.46, 128.34, 128.27, 124.1, 121.7, 118.4; HRMS (ESI) *m/z*: Calcd for $C_{17}H_{12}BrN_4^+$ [M+H]$^+$ 351.0240, found 351.0249.

1-(2-Bromophenyl)-5-(4-quinolinyl)-1*H*-1,2,3-triazole (**9**)

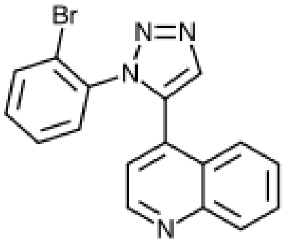

Prepared via base-catalyzed tandem deprotection/click reaction. Yellow crystalline solid, 74% yield, mp 150-152 °C; ^{1}H NMR (400 MHz, $CDCl_3$): δ 8.83 (d, *J* = 4.4 Hz, 1H), 8.18 (d, *J* = 8.1 Hz, 1H), 8.12 (s, 1H), 7.99 (m, 1H), 7.80 (m, 1H), 7.64 (m,

2H), 7.39 (m, 3H), 7.13 (d, *J* = 4.4 Hz, 1H); ^{13}C NMR (400 MHz, $CDCl_3$): δ 149.6, 148.7, 135.6, 135.1, 135.0, 134.1, 132.9, 132.0, 130.41, 130.35, 129.5, 128.6, 128.0, 126.3, 125.0, 122.1, 121.7; HRMS (ESI) *m/z*: Calcd for $C_{17}H_{12}BrN_4^+$ $[M+H]^+$ 351.0240, found 351.0223.

1-(2-Bromophenyl)-5-(5-quinolinyl)-1*H*-1,2,3-triazole (**10**)

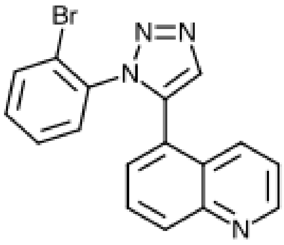

Prepared via base-catalyzed tandem deprotection/click reaction. Yellow crystalline solid, 43% yield, mp 144-146 °C; ^{1}H NMR (400 MHz, $CDCl_3$): δ 8.98 (m, 1H), 8.30 (d, *J* = 8.4 Hz, 1H), 8.18 (d, *J* = 8.5 Hz, 1H), 8.03 (s, 1H), 7.62 (m, 2H), 7.49 (m, 1H), 7.38 (m, 3H), 7.31 (m, 1H); ^{13}C NMR (400 MHz, $CDCl_3$): δ 151.2, 148.3, 136.2, 135.8, 134.8, 134.0, 133.5, 131.8, 131.7, 129.5, 129.2, 128.7, 128.4, 127.2, 124.2, 122.2, 121.7; HRMS (ESI) *m/z*: Calcd for $C_{17}H_{12}BrN_4^+$ $[M+H]^+$ 351.0240, found 351.0246.

1-(2-Bromophenyl)-5-(5-isoquinolinyl)-1*H*-1,2,3-triazole (**11**)

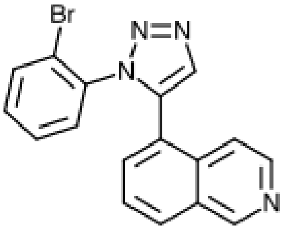

Prepared via base-catalyzed tandem deprotection/click reaction. Orange crystalline solid, 77% yield, mp 159-161 °C; ^{1}H NMR (400 MHz, $CDCl_3$): δ 9.33 (s, 1H), 8.60 (d, *J*= 6.0 Hz, 1H), 8.07 (s, 1H), 8.06 (d, *J*= 8.9 Hz, 1H), 7.79 (d, *J*= 6.0 Hz, 1H), 7.61 (m, 1H), 7.55 (m, 2H), 7.40 (m, 1H), 7.36 (m, 1H), 7.32 (m, 1H); ^{13}C NMR (400 MHz, $CDCl_3$): δ 153.1, 144.4, 135.9, 135.7, 134.7, 134.5, 134.0, 132.7, 131.7, 129.8, 129.5, 128.7, 128.4, 126.6, 123.3, 121.6, 117.7; HRMS (ESI) *m/z*: Calcd for $C_{17}H_{12}BrN_4^+$ $[M+H]^+$ 351.0240, found 351.0230.

1-(2-Bromophenyl)-5-(8-isoquinolinyl)-1*H*-1,2,3-triazole (**12**)

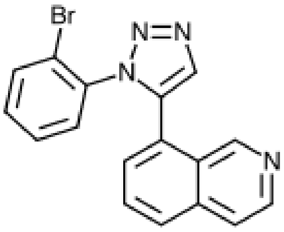

Prepared via base-catalyzed tandem deprotection/click reaction. Yellow solid, 79% yield, mp 126-129.°C; ^{1}H NMR (400 MHz, $CDCl_3$): δ 9.39 (s, 1H), 8.62 (d, *J*= 5.7 Hz, 1H), 8.10 (s, 1H), 7.88 (d, *J*= 8.4 Hz, 1H), 7.73 (d, *J*= 5.7 Hz, 1H), 7.62 (m, 2H), 7.47 (m, 1H), 7.38 (m, 2H), 7.32 (m, 1H); ^{13}C NMR (400 MHz, $CDCl_3$): δ 149.9, 143.5, 136.3, 135.7, 135.6, 135.2, 134.0, 131.8, 130.1, 129.8, 129.7, 128.8, 128.5, 126.7, 124.8, 121.7, 121.0; HRMS (ESI) *m/z*: Calcd for $C_{17}H_{12}BrN_4^+$ [M+H]$^+$ 351.0240, found 351.0250.

Benzo[*i*][1,2,3]triazolo[1,5-*f*]phenanthridine (**13**)

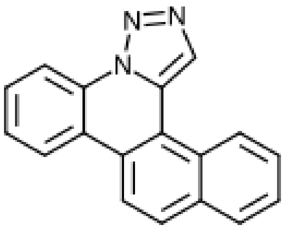

Prepared via microwave annulation reaction. Brown powder, 90% yield, mp 239-241 °C; ^{1}H NMR (400 MHz, $CDCl_3$): δ 9.04 (s, 1H), 9.01 (m, 1H), 8.87 (d, *J*= 8.3 Hz, 1H), 8.65 (d, *J*= 7.7 Hz, 1H), 8.58 (d, *J*= 9.0 Hz, 1H), 8.20 (d, *J*= 9.0 Hz, 1H), 8.10 (d, *J*= 8.3 Hz, 1H), 7.88 (m, 2H), 7.77 (m, 2H); ^{13}C NMR (400 MHz, $CDCl_3$): δ 133.3, 131.2, 130.6, 130.4, 130.1, 129.9, 129.4, 129.3, 128.5, 127.7, 127.4, 127.1, 125.2, 124.3, 122.5, 120.4, 119.1, 117.4; HRMS (ESI) *m/z*: Calcd for $C_{18}H_{12}N_3^+$ [M+H]$^+$ 270.1026, found 270.1019. UV-vis (CH_3CN): λ = 249(4.90), 261(4.95), 276(4.79).

Dibenzo[*c,h*][1,2,3]triazolo[1,5-*a*][2,5]naphthyridine (**14**)

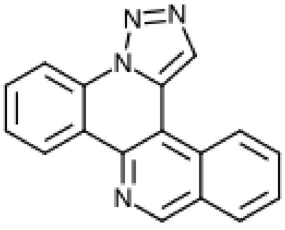

Prepared via microwave annulation reaction. White powder, 43% yield, mp 257-259 °C; ^{1}H NMR (400 MHz, $CDCl_3$): δ 9.64 (s, 1H), 9.38 (d, *J*= 8.3 Hz, 1H), 9.03 (s, 1H), 8.97 (d, *J*= 7.2 Hz, 1H), 8.82 (d, *J*= 9.1 Hz, 1H), 8.32 (d, *J*= 9.0 Hz, 1H) 8.16 (t, *J*=

7.8 Hz, 1H), 7.94 (m, 2H), 7.86 (m, 1H); ^{13}C NMR (400 MHz, $CDCl_3$): δ 154.4, 140.9, 132.8, 132.3, 131.5, 131.2, 130.4, 129.8, 129.7, 128.4, 128.0, 127.9, 126.6, 124.3, 123.8, 116.6, 112.8; HRMS (ESI) *m/z*: Calcd for $C_{17}H_{11}N_4^+$ $[M+H]^+$ 271.0978, found 271.0973. UV-vis (CH_3CN): λ = 257(4.99), 270(sh)(4.95), 287(4.78).

Dibenzo[*c,h*][1,2,3]triazolo[1,5-*a*][2,6]naphthyridine (**15**)

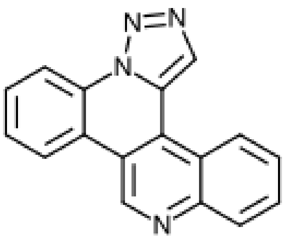

Prepared via microwave annulation reaction. White powder, 72% yield, mp >265 °C; ^{1}H NMR (400 MHz, $CDCl_3$): δ 10.11 (s, 1H), 9.11 (s, 1H), 9.05 (m, 1H), 8.80 (m, 2H), 8.39 (m, 1H), 7.95 (m, 3H), 7.86 (m, 1H); ^{13}C NMR (400 MHz, $CDCl_3$): δ 147.2, 146.4, 131.7, 131.6, 131.2, 130.9, 130.4, 129.1, 128.8, 128.5, 124.8, 124.5, 123.6, 122.7, 121.0, 120.2, 117.7; HRMS (ESI) *m/z*: Calcd for $C_{17}H_{11}N_4^+$ $[M+H]^+$ 271.0978, found 271.0976. UV-vis (CH_3CN): λ = 259(4.81), 266(sh)(4.75), 324(3.97), 339(4.02), 355(4.00).

Benzo[*c*][1,2,3]triazolo[1,5-*a*][2,7]phenanthroline (**16**)

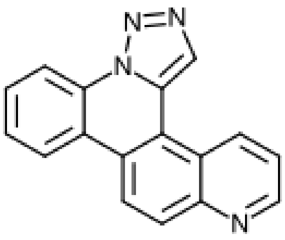

Prepared via microwave annulation reaction. Brown crystalline solid, 31% yield, mp 253 °C (decomp.); ^{1}H NMR (400 MHz, $CDCl_3$): δ 9.28 (d, *J* = 9.6 Hz, 1H), 9.16 (m, 1H), 9.04 (m, 1H), 8.97 (s, 1H), 8.88 (d, *J* = 9.4, 1H), 8.67 (d, *J* = 8.2, 1H), 7.88 (m, 3H); ^{1}H NMR (400 MHz, *d*$_6$-DMSO): δ 9.45 (m, 1H), 9.45 (s, 1H), 9.15 (m, 2H), 8.99 (m, 1H), 8.88 (m,1H), 8.39 (m, 1H), 7.99 (m, 1H), 7.89 (m, 2H); ^{13}C NMR (insufficiently soluble); HRMS (ESI) *m/s*: Calcd for $C_{17}H_{11}N_4^+$ $[M+H]^+$ 271.0978, found 271.0970. UV-vis (CH_3CN): λ = 251(4.60), 269(4.63), 278(4.69), 321(3.89), 334(3.95), 350(3.83).

Benzo[*c*][1,2,3]triazolo[1,5-*a*][2,8]phenanthroline (**17**)

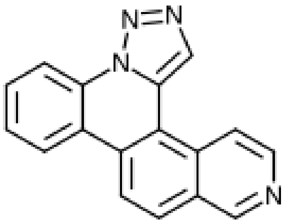

Prepared via microwave annulation reaction. Yellow powder, 49% yield, mp >260 °C; ^{1}H NMR (400 MHz, $CDCl_3$): δ 9.56 (s, 1H), 9.07 (d, *J*= 8.9 Hz, 1H), 9.03 (s, 1H), 8.93 (d, *J*= 6.4, 1H), 8.83 (m, 2H), 8.70 (d, *J*= 7.7 Hz, 1H), 8.41 (d, *J*= 8.9 Hz, 1H), 7.99 (t, *J*= 7.8 Hz, 1H), 7.87 (*J*= 7.4 Hz, 1H); ^{13}C NMR (400 MHz, $CDCl_3$): δ 153.4, 146.1, 132.7, 131.9, 131.3, 130.3, 129.8, 129.7, 129.5, 128.1, 127.9, 124.8, 122.2, 121.8, 118.0, 117.8, 117.7; HRMS (ESI) *m/z*: Calcd for $C_{17}H_{11}N_4^+$ $[M+H]^+$ 271.0978, found 271.0979. UV-vis (CH_3CN): λ = 251(sh)(4.64), 261(4.69), 272(4.65), 288(4.42).

Benzo[*c*][1,2,3]triazolo[1,5-*a*][2,9]phenanthroline (**18**)

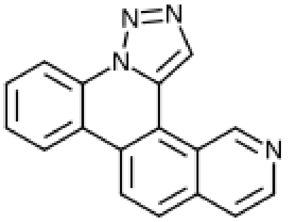

Prepared via microwave annulation reaction. White powder, 46%, yield mp 256-258 °C; ^{1}H NMR (400 MHz, $CDCl_3$): δ 10.36 (s, 1H), 9.10 (s, 1H), 9.07 (d, *J*= 8.3 Hz, 1H), 8.94 (d, *J*= 9.2 Hz, 1H), 8.84 (d, *J*= 5.7 Hz, 1H), 8.65 (d, *J*= 8.3 Hz, 1H), 8.25 (d, *J*= 9.6 Hz, 1H), 8.13 (m, 1H), 7.69 (t, *J*= 7.7 Hz, 1H), 7.85 (t, *J*= 7.3 Hz, 1H); ^{13}C NMR (400 MHz, $CDCl_3$): δ 149.2, 145.0, 136.3, 131.6, 130.8, 130.6, 129.4, 128.9, 128.0, 127.9, 125.2, 124.6, 124.3, 121.9, 121.5, 119.0, 117.7; HRMS (ESI) *m/z*: Calcd for $C_{17}H_{11}N_4^+$ $[M+H]^+$ 271.0978, found 271.0981. UV-vis (CH_3CN): λ = 251(4.53), 256(4.85), 270(4.80), 287(4.64).

5-(2-Bromophenyl)-1-(1-naphthalenyl)-1*H*-1,2,3-triazole (**25**)

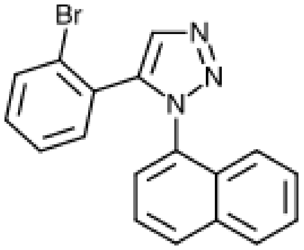

Prepared via base-catalyzed tandem deprotection/click reaction. Orange crystalline solid, 72% yield, mp 129-131 °C; ^{1}H NMR (400 MHz, $CDCl_3$): δ 8.10 (s, 1H), 7.95 (m, 1H), 7.91

(m, 1H), 7.56 (m, 4H), 7.46 (m, 2H), 7.14 (m, 1H), 7.09 (m, 1H), 7.04 (m, 1H); ^{13}C NMR (400 MHz, $CDCl_3$): δ 138.4, 134.6, 134.2, 133.4, 132.6, 131.7, 131.1, 130.6, 129.7, 128.4, 128.3, 127.8, 127.4, 127.1, 125.3, 124.9, 124.2, 123.0; HRMS (ESI) *m/z*: Calcd for $C_{18}H_{13}BrN_3^+$ $[M+H]^+$ 350.0287, found 350.0271.

5-(2-Bromophenyl)-1-(4-isoquinolinyl)-1*H*-1,2,3-triazole (**26**)

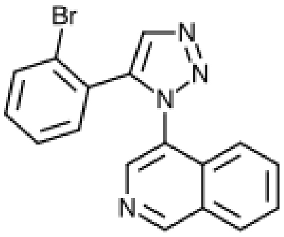

Prepared via base-catalyzed tandem deprotection/click reaction. Yellow solid, 83% yield, mp 184-187 °C; ^{1}H NMR (400 MHz, $CDCl_3$): δ 9.33 (s, 1H), 8.41 (s, 1H), 8.11 (d, *J*= 8.2 Hz, 1H), 8.08 (s, 1H), 7.82 (m, 2H), 7.75 (m, 1H), 7.57 (m, 1H), 7.20 (m, 3H); ^{13}C NMR (400 MHz, $CDCl_3$): δ 154.5, 141.0, 138.7, 134.8, 133.6, 132.3, 132.2, 132.0, 131.5, 129.1, 128.6, 128.2, 127.98, 127.95, 127.7, 124.2, 122.5; HRMS (ESI) *m/z*: Calcd for $C_{17}H_{12}BrN_4^+$ $[M+H]^+$ 351.0240, found 351.0250.

5-(2-Bromophenyl)-1-(4-quinolinyl)-1*H*-1,2,3-triazole (**27**)

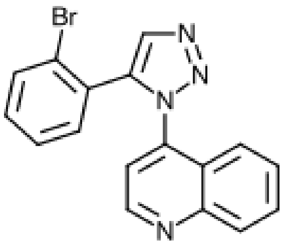

Prepared via base-catalyzed tandem deprotection/click reaction. Brown crystalline solid, 79% yield, mp 133-134 °C; ^{1}H NMR (400 MHz, $CDCl_3$): δ 8.93 (d, *J* = 4.6 Hz, 1H), 8.20 (m, 1H), 8.08 (s, 1H), 7.82 (m, 2H), 7.61 (m, 2H), 7.22 (m, 3H), 7.12 (m, 1H); ^{13}C NMR (400 MHz, $CDCl_3$): δ 150.0, 149.8, 140.5, 138.3, 134.9, 133.7, 131.8, 131.6, 130.8, 129.9, 128.4, 127.8, 127.7, 124.1, 123.8, 123.6, 118.3; HRMS (ESI) *m/z*: Calcd for $C_{17}H_{12}BrN_4^+$ $[M+H]^+$ 351.0240, found 351.0223.

5-(2-Bromophenyl)-1-(5-quinolinyl)-1*H*-1,2,3-triazole (**28**)

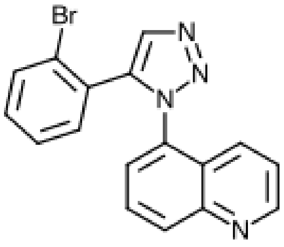

Prepared via base-catalyzed tandem deprotection/click reaction. Orange crystalline solid, 74% yield, mp 122-123 °C; ^{1}H NMR (400 MHz, $CDCl_3$): δ 8.99 (m, 1H), 8.25 (d, *J* = 8.6 Hz, 1H), 8.07 (s, 1H), 8.06 (d, *J* = 8.2 Hz, 1H), 7.70 (m, 1H), 7.58 (m, 1H), 7.47 (m, 2H), 7.19 (m, 2H), 7.09 (m, 1H); ^{13}C NMR (400 MHz, $CDCl_3$): δ 151.6, 148.6, 138.5, 134.7, 133.6, 132.4, 132.1, 131.91, 131.88, 131.4, 128.4, 128.1, 127.6, 125.4, 125.0, 124.2, 122.6; HRMS (ESI) *m/z*: Calcd for $C_{17}H_{12}BrN_4^+$ $[M+H]^+$ 351.0240, found 351.0232.

5-(2-Bromophenyl)-1-(5-isoquinolinyl)-1*H*-1,2,3-triazole (**29**)

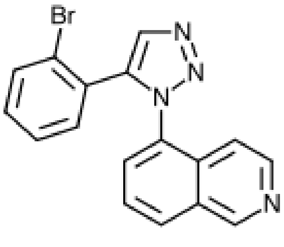

Prepared via base-catalyzed tandem deprotection/click reaction. Brown solid, 81% yield, mp 161-163 °C; ^{1}H NMR (400 MHz, $CDCl_3$): δ 9.35 (s, 1H), 8.59 (d, *J*= 6.0 Hz, 1H), 8.11 (m, 1H), 8.08 (s, 1H), 7.62 (m, 2H), 7.58 (m, 1H), 7.51 (d, *J*= 6.0 Hz, 1H), 7.19 (m, 2H), 7.10 (m, 1H); ^{13}C NMR (400 MHz, $CDCl_3$): δ 152.8, 144.8, 138.4, 134.8, 133.6, 132.2, 131.83, 131.82, 131.4, 130.2, 129.12, 129.09, 128.0, 127.6, 126.5, 124.2, 115.9; HRMS (ESI) *m/z*: Calcd for $C_{17}H_{12}BrN_4^+$ $[M+H]^+$ 351.0240, found 351.0222.

5-(2-Bromophenyl)-1-(8-isoquinolinyl)-1*H*-1,2,3-triazole (**30**)

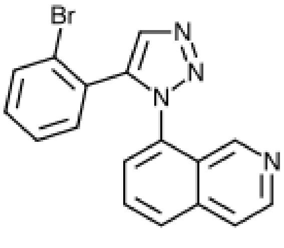

Prepared via base-catalyzed tandem deprotection/click reaction. Orange solid, 73% yield, mp 179-182 °C; ^{1}H NMR (400 MHz, $CDCl_3$): δ 9.20 (s, 1H), 8.65 (d, *J*= 5.8 Hz, 1H), 8.08 (s, 1H), 7.98 (d, *J*= 8.4 Hz, 1H), 7.82 (d, *J*= 5.8 Hz, 1H), 7.73 (m, 1H), 7.57 (m, 1H), 7.50 (m, 1H) 7.21 (m, 3H); ^{13}C NMR (400 MHz, $CDCl_3$): δ

148.6, 144.3, 138.7, 136.7, 134.8, 133.6, 133.2, 132.0, 131.4, 129.6, 129.2, 128.1, 127.6, 126.2, 124.4, 124.3, 120.3; HRMS (ESI) *m/z*: Calcd for $C_{17}H_{12}BrN_4^+$ $[M+H]^+$ 351.0240, found 351.0233.

Benzo[*c*][1,2,3]triazolo[1,5-*f*]phenanthridine (**31**)

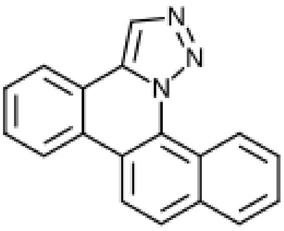

Prepared via thermal annulation reaction. Brown solid, 65% yield, mp 161 °C (decomp.); ^{1}H NMR (400 MHz, $CDCl_3$): δ 10.67 (d, *J* = 8.3 Hz, 1H), 8.66 (s, 1H), 8.63 (t, *J* = 8.8 Hz, 2H), 8.33 (m, 1H), 8.16 (d, *J* = 8.9 Hz, 1H), 8.10 (d, *J* = 8.6 Hz, 1H), 7.92 (m, 1H), 7.80 (m, 3H); ^{13}C NMR (400 MHz, $CDCl_3$): δ 134.1, 133.2, 129.40, 129.39, 128.764, 128.758, 128.6, 128.41, 128.40, 128.3, 127.7, 125.9, 124.8, 124.3, 123.5, 121.9, 120.5, 120.2; HRMS (ESI) *m/z*: Calcd for $C_{18}H_{12}N_3^+$ $[M+H]^+$ 270.1026, found 270.1023. UV-vis (CH_3CN): λ = 250(sh)(4.74) 257(4.82), 272(4.56), 282(4.55), 295(sh)(4.17), 326(3.9), 341(3.56), 353(3.31).

Dibenzo[*c,h*][1,2,3]triazolo[1,5-*a*][1,5]naphthyridine (**32**)

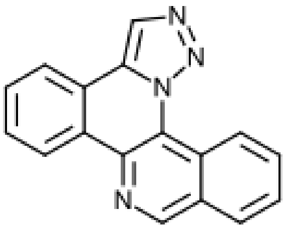

Prepared via thermal annulation reaction. Yellow powder, 34% yield, mp 188-190 °C; ^{1}H NMR (400 MHz, $CDCl_3$): δ 10.47 (d, *J*= 8.8 Hz, 1H), 9.55 (s, 1H), 9.29 (m, 1H), 8.67 (s, 1H), 8.30 (m, 2H), 8.14 (m, 1H), 7.93 (m, 1H), 7.87 (m, 2H); ^{13}C NMR (400 MHz, $CDCl_3$): δ 152.8, 135.0, 133.1, 132.7, 130.0. 129.8, 129.6, 129.1, 128.9, 128.6, 127.7, 127.2, 126.1, 126.0, 123.9, 122.9, 122.4; HRMS (ESI) *m/z*: Calcd for $C_{17}H_{11}N_4^+$ $[M+H]^+$ 271.0978, found 271.0966. UV-vis (CH_3CN): λ = 246(4.79), 254(4.83), 265(sh)(4.53), 277(4.48), 301(4.05), 324(4.04), 340(3.86), 356(3.85).

Dibenzo[*c,h*][1,2,3]triazolo[1,5-*a*][1,6]naphthyridine (**33**)

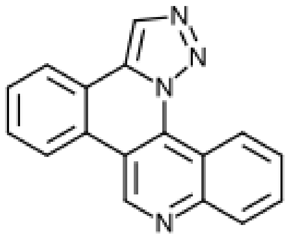

Prepared via thermal annulation reaction. White powder, 42% yield, mp 236 °C (decomp.); ^{1}H NMR (400 MHz, $CDCl_3$): δ 10.47 (m, 1H), 10.14 (s, 1H), 8.80 (m, 1H), 8.68 (s, 1H), 8.37 (m, 1H), 8.35 (m, 1H), 7.96 (m, 2H), 7.87 (m, 2H); ^{13}C NMR (400 MHz, $CDCl_3$): δ 148.5, 146.7, 134.3, 132.4, 130.9, 130.33, 130.26, 129.7, 129.0, 128.3, 126.4, 124.9, 123.0, 122.5, 118.5, 114.3; HRMS (ESI) *m/z*: Calcd for $C_{17}H_{11}N_4^+$ $[M+H]^+$ 271.0978, found 271.0979. UV-vis (CH_3CN): λ = 248(sh)(4.42), 255(4.55), 270(4.19), 281(4.17), 338(3.43), 354(3.45).

Benzo[*c*][1,2,3]triazolo[1,5-*a*][1,7]phenanthroline (**34**)

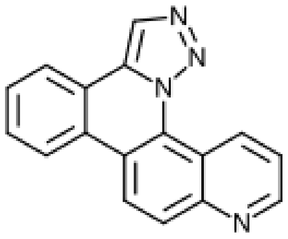

Prepared via thermal annulation reaction. Orange solid, 31% yield, mp 163 °C (decomp.); ^{1}H NMR (400 MHz, $CDCl_3$): δ 10.99 (d, *J* = 8.6 Hz, 1H), 9.15 (m, 1H), 8.87 (d, *J* = 9.2 Hz, 1H), 8.68 (m, 1H), 8.66 (s, 1H), 8.44 (d, *J* = 9.2 Hz, 1H), 8.34 (m, 1H), 7.84 (m, 3H); ^{13}C NMR (400 MHz, $CDCl_3$): δ 151.3, 148.8, 137.1, 133.5, 130.3, 129.9, 129.5, 127.6, 127.2, 126.3, 124.7, 124.3, 123.9, 122.9, 122.3, 120.91, 120.86; HRMS (ESI) *m/z*: Calcd for $C_{17}H_{11}N_4^+$ $[M+H]^+$ 271.0978, found 271.0971. UV-vis (CH_3CN): λ = 250(sh)(4.53), 256(4.63), 271(4.36), 281(4.34), 322(3.64), 336(3.54), 352(3.39).

Benzo[*c*][1,2,3]triazolo[1,5-*a*][1,8]phenanthroline (**35**)

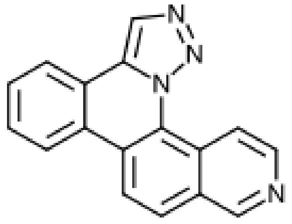

Prepared via thermal annulation reaction. Yellow powder, 49% yield, mp 227-229 °C; ^{1}H NMR (400 MHz, $CDCl_3$): δ 10.60 (m, 1H), 9.58 (s, 1H), 8.94 (d, *J*= 6.5 Hz, 1H), 8.88 (d, *J*= 8.8 Hz, 1H), 8.70 (m, 1H), 8.69 (s, 1H), 8.38 (m, 2H), 7.91 (m, 2H); ^{13}C

NMR (400 MHz, $CDCl_3$): δ 152.5, 146.2, 133.3, 130.2, 130.0, 128.6, 128.2, 127.9, 127.3, 126.5, 126.3, 124.9, 124.3, 123.9, 122.9, 122.2, 120.7; HRMS (ESI) *m/z*: Calcd for $C_{17}H_{11}N_4^+$ $[M+H]^+$ 271.0978, found 271.0988. UV-vis (CH_3CN): λ = 250(sh)(4.62), 257(4.47), 278(4.19), 328(3.45).

Benzo[*c*][1,2,3]triazolo[1,5-*a*][1,9]phenanthroline (**36**)

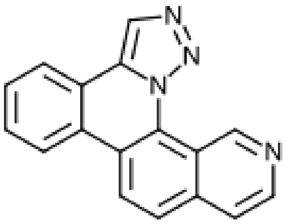

Prepared via thermal annulation reaction. Yellow crystalline solid, 51% yield, mp 201-203 °C; ^1H NMR (400 MHz, $CDCl_3$): δ 11.93 (s, 1H), 8.89 (d, *J*= 5.6 Hz, 2H), 8.68 (s, 1H), 8.65 (m, 1H), 8.36 (m, 1H), 8.16 (d, *J*= 8.2 Hz, 1H), 7.98 (m, 1H), 7.86 (m, 2H); ^{13}C NMR (400 MHz, $CDCl_3$): δ 152.8, 145.0, 137.0, 133.5, 130.0, 129.7, 127.6, 127.4, 127.3, 126.3, 125.3, 124.8, 123.8, 122.4, 121.5, 120.6, 120.5; HRMS (ESI) *m/z*: Calcd for $C_{17}H_{11}N_4^+$ $[M+H]^+$ 271.0978, found 271.0989. UV-vis (CH_3CN): λ = 248(sh)(4.69), 254(4.75), 268(4.44), 278(4.38), 323(3.90), 339(3.59), 356(3.55).

5-(1-Naphthalenyl)-1-phenyl-1*H*-1,2,3-triazole (**37**)

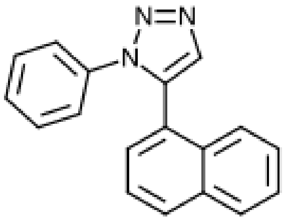

Prepared via base-catalyzed tandem deprotection/click reaction. Orange solid, 75% yield, mp 104-106 °C; ^1H NMR (400 MHz, $CDCl_3$): δ 7.97 (s, 1H), 7.95 (d, *J*= 8.4 Hz, 1H), 7.92 (d, *J*= 8.5 Hz, 1H), 7.66 (d, *J*= 8.4 Hz, 1H), 7.54 (m, 1H), 7.47 (t, *J*= 7.7 Hz, 2H), 7.30 (m, 6H); ^{13}C NMR (400 MHz, $CDCl_3$): δ 136.8, 135.9, 135.6, 133.7, 131.9, 130.3, 129.3, 129.1, 129.0, 128.7, 127.4, 126.7, 125.3, 124.9, 124.7, 124.2; HRMS (ESI) *m/z*: Calcd for $C_{18}H_{14}N_3^+$ $[M+H]^+$ 272.1182, found 272.1177. UV-vis (CH_3CN): λ = <230(4.78), 270(sh)(3.34).

5-(4-Isoquinolinyl)-1-phenyl-1*H*-1,2,3-triazole (**38**)

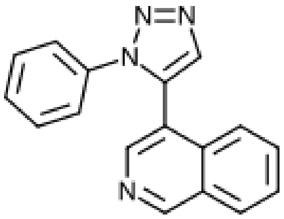

Prepared via base-catalyzed tandem deprotection/click reaction. Orange solid, 63% yield, mp 159-161 °C; ^{1}H NMR (400 MHz, $CDCl_3$): δ 9.33 (s, 1H), 8.40 (s, 1H), 8.08 (m, 1H), 8.04 (s, 1H), 7.70 (m, 2H), 7.65 (m, 1H), 7.31 (m, 5H); ^{13}C NMR (400 MHz, $CDCl_3$): δ 154.3, 144.5, 136.4, 136.0, 134.3, 133.0, 132.0, 129.6, 129.4, 128.5, 128.31, 128.29, 124.4, 123.9, 119.1; HRMS (ESI) *m/z*: Calcd for $C_{17}H_{13}N_4^+$ $[M+H]^+$ 273.1135, found 273.1138. UV-vis (CH_3CN): λ = 243(4.45), 255(4.51).

1-Phenyl-5-(4-quinolinyl)-1*H*-1,2,3-triazole (**39**)

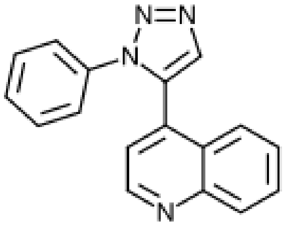

Prepared via base-catalyzed tandem deprotection/click reaction. Orange crystalline solid, 89% yield, mp 145-147 °C; ^{1}H NMR (400 MHz, $CDCl_3$): δ 8.91 (d, *J* = 4.4 Hz, 1H), 8.22 (d, *J* = 8.5 Hz, 1H), 8.06 (s, 1H), 7.80 (m, 1H), 7.76 (m, 1H), 7.58 (m, 1H), 7.34 (m, 5H), 7.21 (d, *J* = 4.4 Hz, 1H); ^{13}C NMR (400 MHz, $CDCl_3$): δ 149.8, 148.8, 136.3, 135.8, 133.7, 133.5, 130.5, 130.4, 129.7, 129.6, 128.1, 126.4, 124.8, 124.4, 122.8; HRMS (ESI) *m/z*: Calcd for $C_{17}H_{13}N_4^+$ $[M+H]^+$ 273.1135, found 273.1133. UV-vis (CH_3CN): λ = 270(3.99), 281(3.94).

1-Phenyl-5-(5-quinolinyl)-1*H*-1,2,3-triazole (**40**)

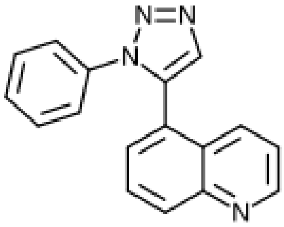

Prepared via base-catalyzed tandem deprotection/click reaction. Orange crystalline solid, 48% yield, mp 153-155 °C; ^{1}H NMR (400 MHz, $CDCl_3$): δ 8.97 (m, 1H), 8.26 (d, *J* = 8.7 Hz, 1H), 8.01 (d, *J* = 8.3 Hz, 1H), 7.99 (s, 1H), 7.75 (m, 1H), 7.47 (m, 1H), 7.40 (m, 1H), 7.30 (m, 5H); ^{13}C NMR (400 MHz, $CDCl_3$): δ 151.2, 148.4,

136.5, 135.7, 134.7, 133.1, 131.8, 129.6, 129.5, 129.3, 129.0, 127.1, 125.0, 124.2, 122.2; HRMS (ESI) *m/z*: Calcd for $C_{17}H_{13}N_4^+$ $[M+H]^+$ 273.1135, found 273.1127. UV-vis (CH_3CN): λ = <230(4.48), 286(3.76), 316(3.45).

5-(5-Isoquinolinyl)-1-phenyl-1*H*-1,2,3-triazole (**41**)

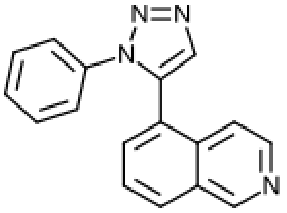

Prepared via base-catalyzed tandem deprotection/click reaction. Brown crystalline solid, 77% yield, mp 169-171 °C; ^{1}H NMR (400 MHz, $CDCl_3$): δ 9.35 (s, 1H), 8.52 (d, *J*= 6.0 Hz, 1H), 8.11 (d, *J*= 8.1 Hz, 1H), 8.01 (s, 1H), 7.63 (m, 2H), 7.48 (d, *J*= 6.0 Hz, 1H), 7.32 (m, 5H); ^{13}C NMR (400 MHz, $CDCl_3$): δ 153.1, 144.4, 136.4, 135.6, 134.5, 134.4, 133.2, 129.9, 129.5, 129.3, 128.7, 126.9, 124.3, 124.1; HRMS (ESI) *m/z*: Calcd for $C_{17}H_{13}N_4^+$ $[M+H]^+$ 273.1135, found 273.1122. UV-vis (CH_3CN): λ = <230((4.57), 244(4.52), 255(4.58).

5-(8-Isoquinolinyl)-1-phenyl-1*H*-1,2,3-triazole (**42**)

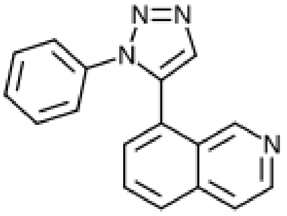

Prepared via base-catalyzed tandem deprotection/click reaction. Brown solid, 79% yield, mp 139-141 °C; ^{1}H NMR (400 MHz, $CDCl_3$): δ 9.13 (s, 1H), 8.60 (d, *J*= 5.8 Hz, 1H), 8.05 (s, 1H), 7.98 (d, *J*= 8.4 Hz, 1H), 7.80 (d, *J*= 5.6 Hz, 1H), 7.76 (t, *J*= 7.7 Hz, 1H), 7.51 (d, *J*= 7.1 Hz, 1H), 7.31(m, 5H); ^{13}C NMR (400 MHz, $CDCl_3$): δ 149.7, 143.5, 136.42, 136.39, 135.9, 134.1, 130.5, 130.1, 129.6, 129.4, 128.9, 126.7, 125.6, 124.5, 121.0; HRMS (ESI) *m/z*: Calcd for $C_{17}H_{13}N_4^+$ $[M+H]^+$ 273.1135, found 273.1132. UV-vis (CH_3CN): λ = 242(sh)(4.49), 248(4.51), 254(4.58).

1-(1-Naphthalenyl)-5-phenyl-1*H*-1,2,3-triazole (**43**)

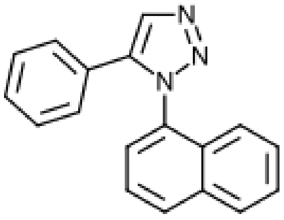

Prepared via base-catalyzed click reaction. Orange crystalline solid, 82% yield, mp 134-136 °C. ^{1}H NMR (400 MHz, $CDCl_3$): δ 8.07 (s, 1H), 8.04 (d, *J* = 8.3 Hz, 1H), 7.97 (d, *J* = 8.2 Hz, 1H), 7.57 (m, 2H), 7.51 (m, 1H), 7.47 (m, 1H), 7.39 (m, 1H), 7.25 (m, 1H), 7.21 (m, 2H), 7.15 (m, 2H); ^{13}C NMR (400 MHz, $CDCl_3$): δ 139.9, 134.3, 133.3, 132.6, 130.8, 130.0, 129.3, 128.9, 128.4, 128.1, 128.0, 127.2, 126.6, 125.6, 125.2, 122.7; HRMS (ESI) *m/z*: Calcd for $C_{18}H_{14}N_3^+$ $[M+H]^+$ 272.1182, found 272.1177. UV-vis (CH_3CN): λ = <230(4.58), 270(3.99), 281(3.94).

1-(4-Isoquinolinyl)-5-phenyl-1*H*-1,2,3-triazole (**44**)

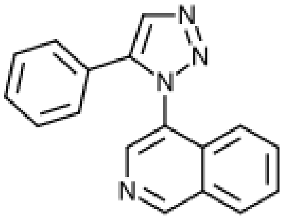

Prepared via base-catalyzed click reaction. Orange solid, 80% yield, mp 156-159 °C; ^{1}H NMR (400 MHz, $CDCl_3$): δ 9.43 (s, 1H), 8.50 (s, 1H), 8.17 (m, 1H), 8.08 (s, 1H), 7.79 (m, 2H), 7.57 (m, 1H), 7.30 (m, 1H), 7.25 (m, 2H), 7.16 (m, 2H); ^{13}C NMR (400 MHz, $CDCl_3$): δ 154.7, 141.7, 140.3, 133.0, 132.54, 132.50, 129.7. 129.2, 129.1, 128.8, 128.7, 128.2, 128.1, 126.2, 122.0; HRMS (ESI) *m/z*: Calcd for $C_{17}H_{13}N_4^+$ $[M+H]^+$ 273.1135, found 273.1145. UV-vis (CH_3CN): λ = <230(4.79), 308(3.64), 320(3.72).

5-Phenyl-1-(4-quinolinyl)-1*H*-1,2,3-triazole (**45**)

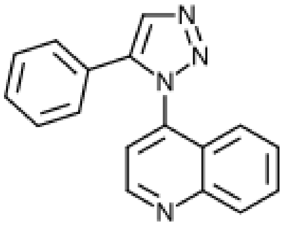

Prepared via base-catalyzed click reaction. Orange crystalline solid, 81% yield, 140-141 °C; ^{1}H NMR (400 MHz, $CDCl_3$): δ 8.14 (d, *J* = 8.5 Hz, 1H), 7.93 (s, 1H), 7.70 (m, 1H), 7.48 (m, 2H), 7.19 (m, 2H), 7.12 (m, 2H), 7.01 (m, 2H); ^{13}C NMR (400 MHz, $CDCl_3$): δ 150.3, 149.9, 141.1, 139.9, 133.0, 130.9, 130.1, 129.8, 129.2, 128.6,

128.1, 126.0, 124.2, 123.1, 119.1; HRMS (ESI) *m/z*: Calcd for $C_{17}H_{13}N_4^+$ $[M+H]^+$ 273.1135, found 273.1129. UV-vis (CH_3CN): λ = 231(4.76), 303(3.68), 317(3.56).

5-Phenyl-1-(5-quinolinyl)- 1*H*-1,2,3-triazole (**46**)

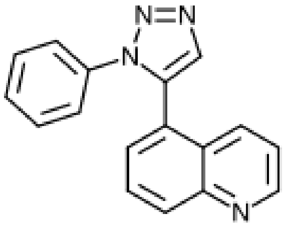

Prepared via base-catalyzed click reaction. Orange crystalline solid, 79% yield, mp 106-107 °C; ^{1}H NMR (400 MHz, $CDCl_3$): δ 9.01 (m, 1H), 8.33 (d, *J* = 8.6 Hz, 1H), 8.07 (s, 1H), 7.81 (m, 2H), 7.54 (m, 1H), 7.44 (m, 1H), 7.30 (m, 1H), 7.24 (m, 2H), 7.13 (m, 2H); ^{13}C NMR (400 MHz, $CDCl_3$): δ 151.7, 148.7, 140.0, 132.9, 132.8, 132.3, 131.4, 129.6, 129.1, 128.7, 128.1, 126.3, 126.0, 125.2, 122.8; HRMS (ESI) *m/z*: Calcd for $C_{17}H_{13}N_4^+$ $[M+H]^+$ 273.1135, found 273.1123. UV-vis (CH_3CN): λ = <230(4.56), 248(sh)(4.14), 302(3.45), 315(3.41).

1-(5-Isoquinolinyl)-5-phenyl-1*H*-1,2,3-triazole (**47**)

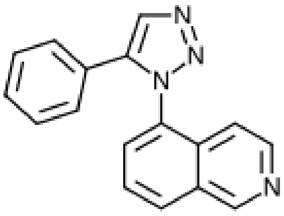

Prepared via base-catalyzed click reaction. Brown crystalline solid, 80% yield, mp 115-118 °C; ^{1}H NMR (400 MHz, $CDCl_3$): δ 9.41 (s, 1H), 8.55 (d, *J*= 6.0 Hz, 1H) 8.21 (m, 1H), 8.07 (s, 1H), 7.72 (m, 2H), 7.31 (m, 2H), 7.24 (m, 2H), 7.13 (m, 2H); ^{13}C NMR (400 MHz, $CDCl_3$): δ 152.8, 144.9, 139.9, 132.9, 132.5, 132.4, 130.4, 129.8, 129.7, 129.18, 129.16, 128.1, 126.9, 126.3, 115.6; HRMS (ESI) *m/z*: Calcd for $C_{17}H_{13}N_4^+$ $[M+H]^+$ 273.1135, found 273.1125. UV-vis (CH_3CN): λ = <230(4.62), 309(3.55), 322(3.64).

1-(8-Isoquinolinyl)-5-phenyl-1*H*-1,2,3-triazole (**48**)

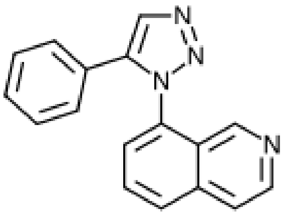

Prepared via base-catalyzed click reaction. Brown crystalline solid, 77% yield, mp 165-167 °C; ^{1}H NMR (400 MHz, $CDCl_3$): δ 8.95 (s, 1H), 8.65 (d, *J*= 5.8 Hz, 1H), 8.08 (s, 1H), 8.05 (d, *J*= 8.4 Hz, 1H), 7.82 (m, 2H), 7.60 (m, 1H), 7.30 (m, 1H), 7.24 (m, 2H), 7.16 (m, 2H); ^{13}C NMR (400 MHz, $CDCl_3$): δ 148.3, 144.5, 140.2, 136.8, 133.7, 133.0, 129.9, 129.7, 129.4, 129.2, 128.2, 126.8, 126.2, 124.5, 120.4; HRMS (ESI) *m/z*: Calcd for $C_{17}H_{13}N_4^+$ $[M+H]^+$ 273.1135, found 273.1123. UV-vis (CH_3CN): λ = <230(4.54), 308(3.54), 314(3.54), 320(3.64).

1-(2-Bromophenyl)-5-(1-naphthalenyl)-1*H*-1,2,3-triazole (**7**)

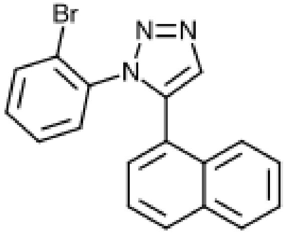

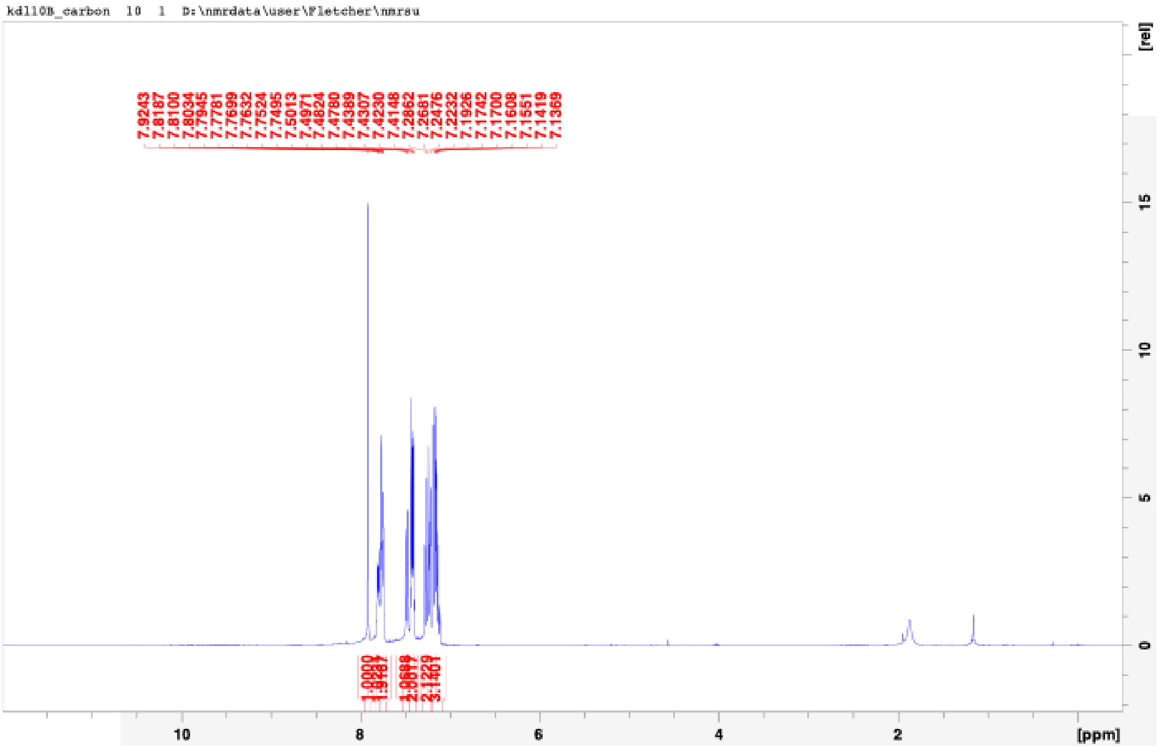

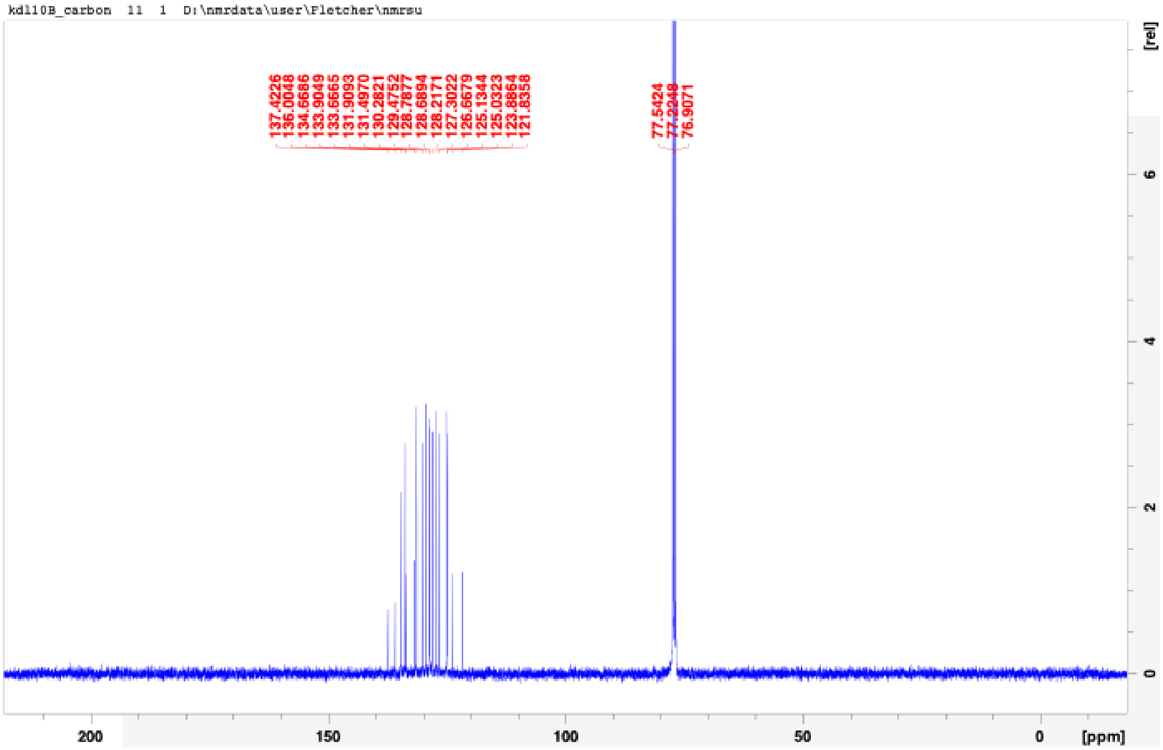

1-(2-Bromophenyl)-5-(4-isoquinolinyl)-1*H*-1,2,3-triazole (**8**)

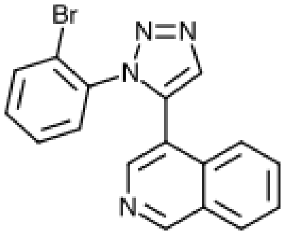

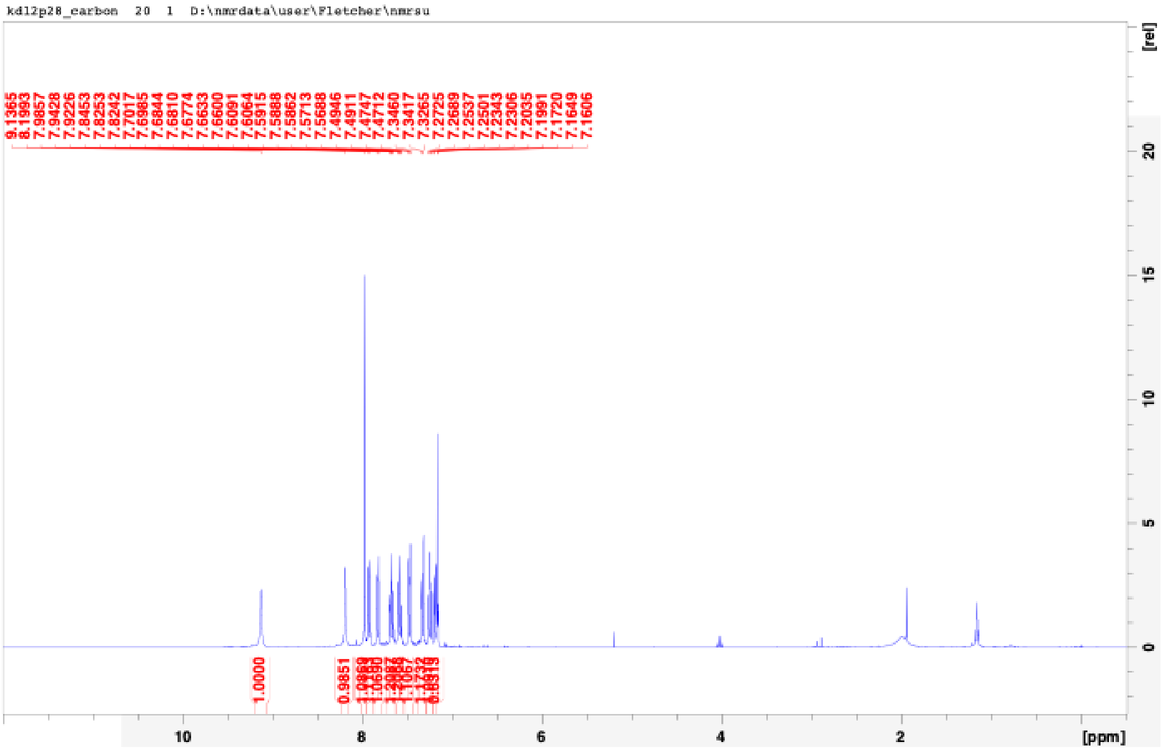

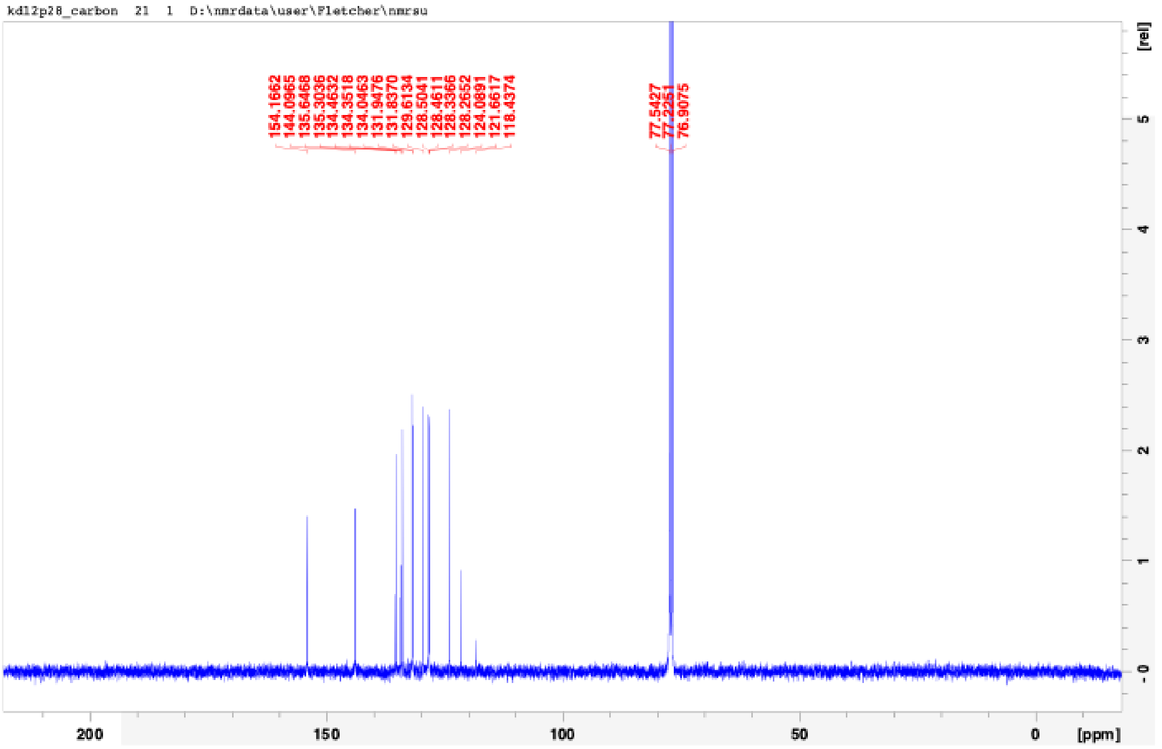

1-(2-Bromophenyl)-5-(4-quinolinyl)-1*H*-1,2,3-triazole (**9**)

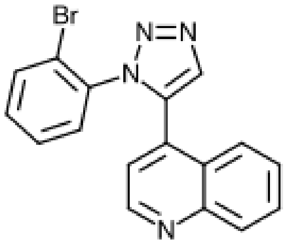

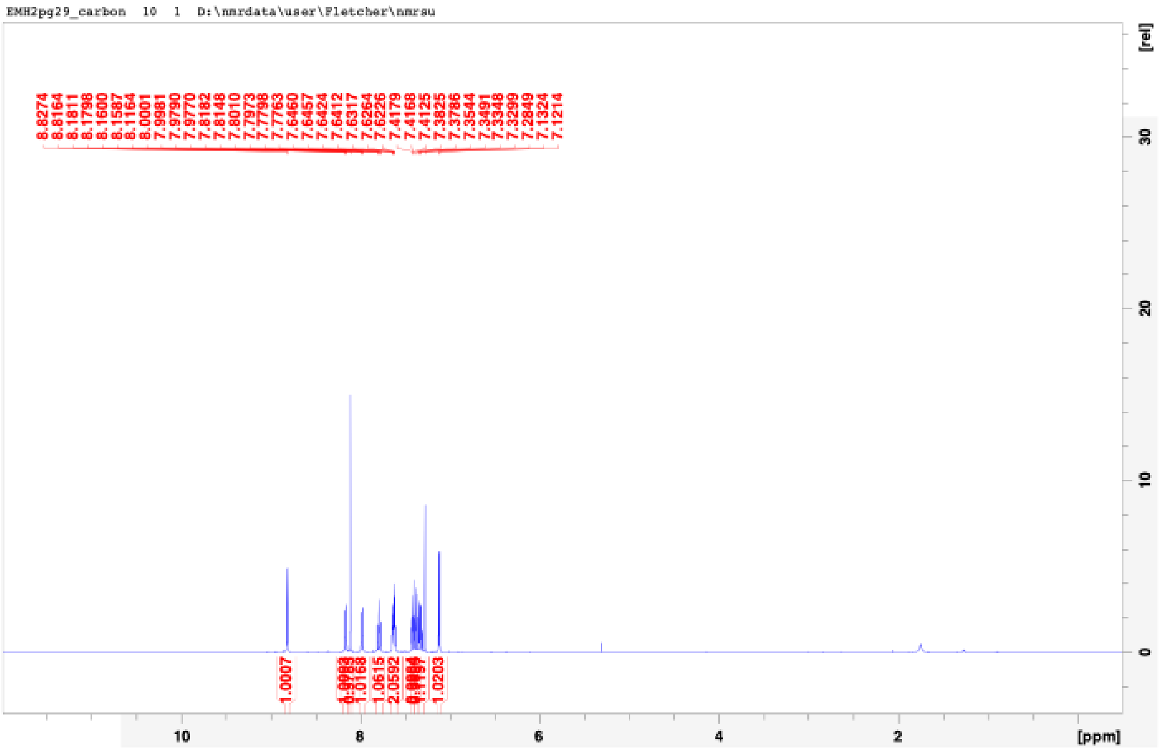

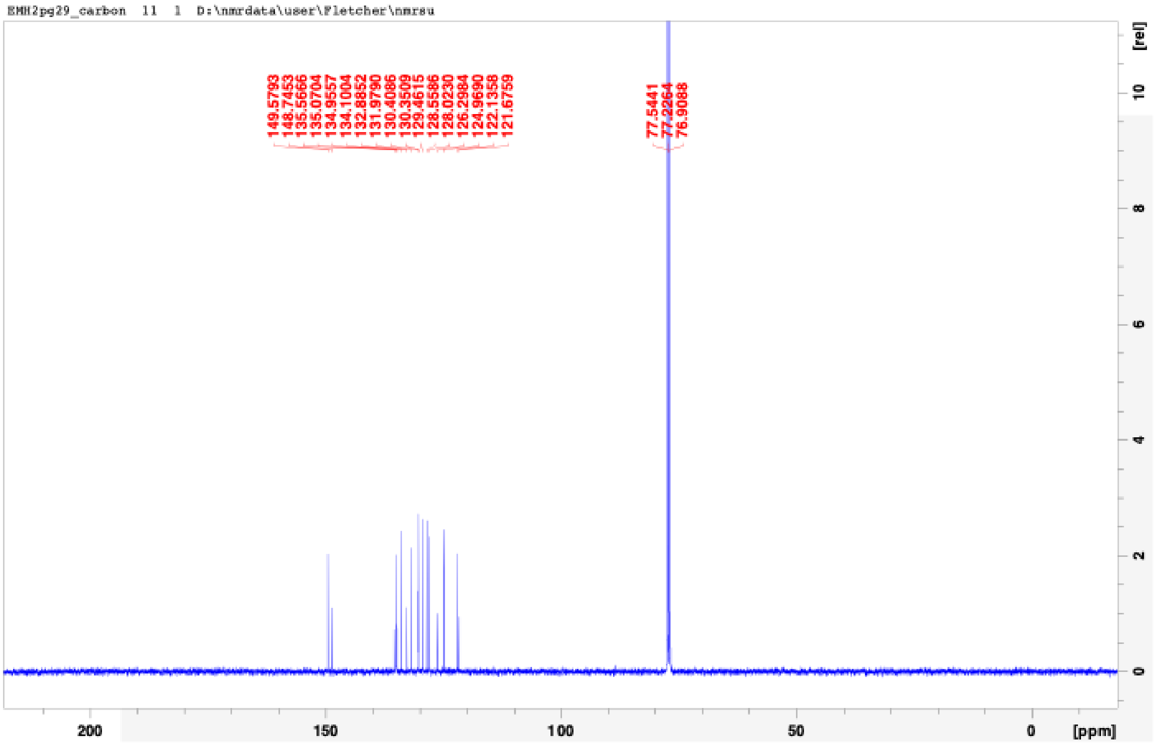

1-(2-Bromophenyl)-5-(5-quinolinyl)-1*H*-1,2,3-triazole (**10**)

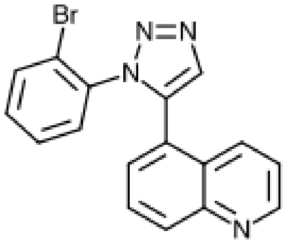

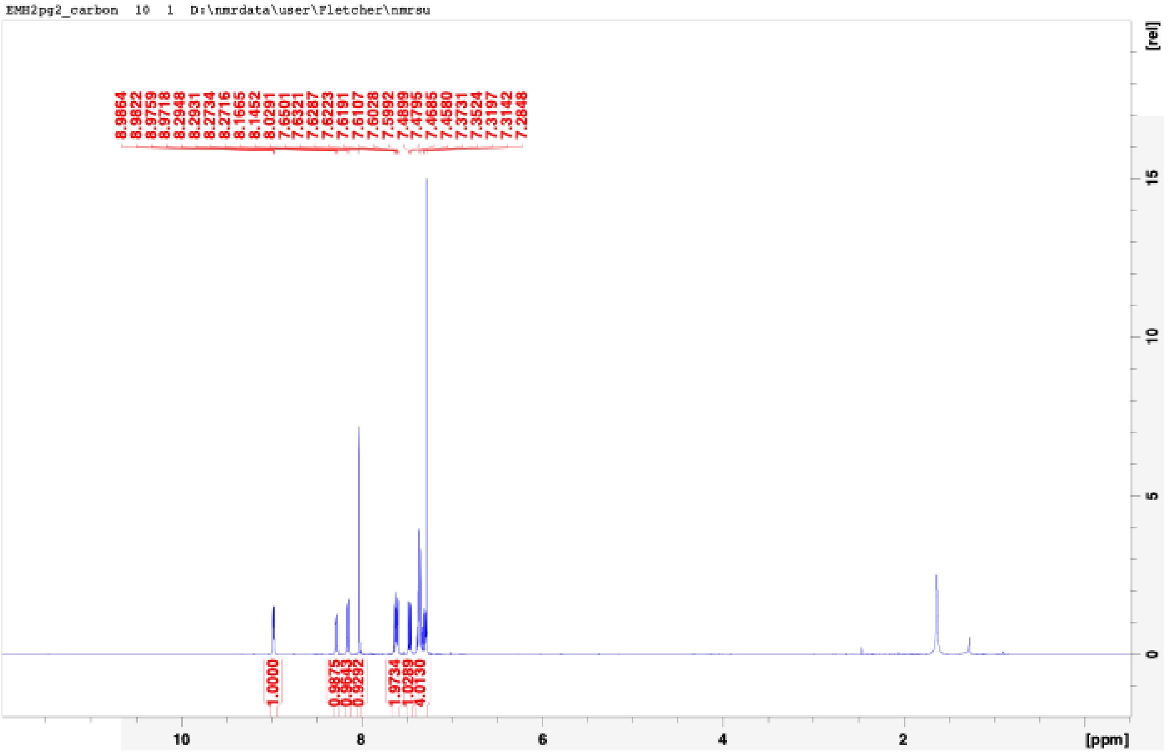

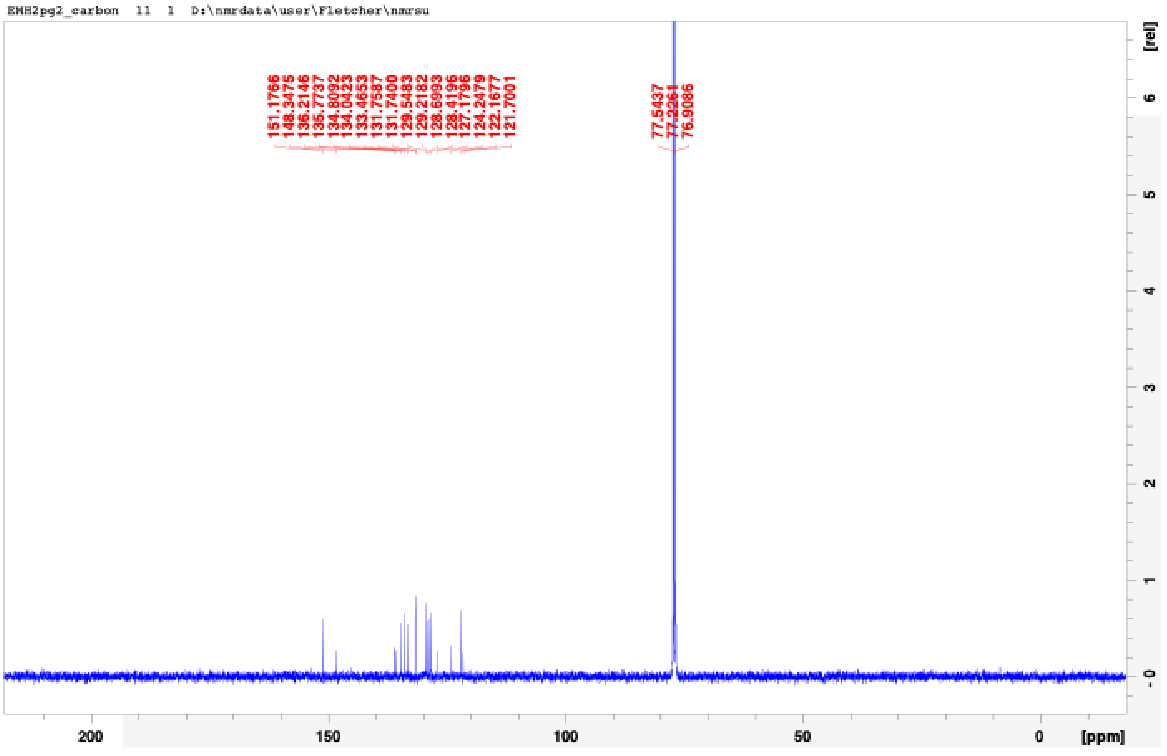

1-(2-Bromophenyl)-5-(5-isoquinolinyl)-1*H*-1,2,3-triazole (**11**)

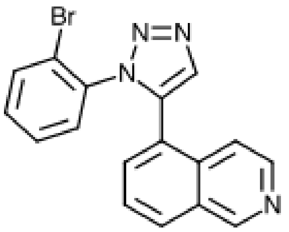

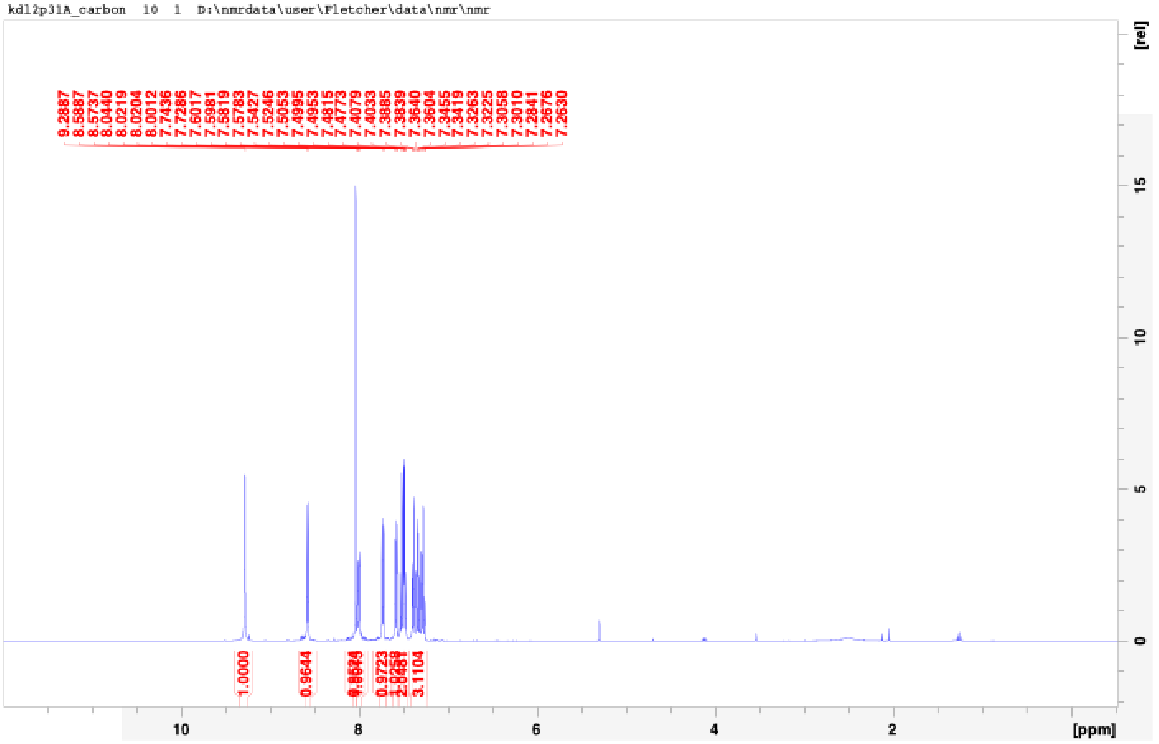

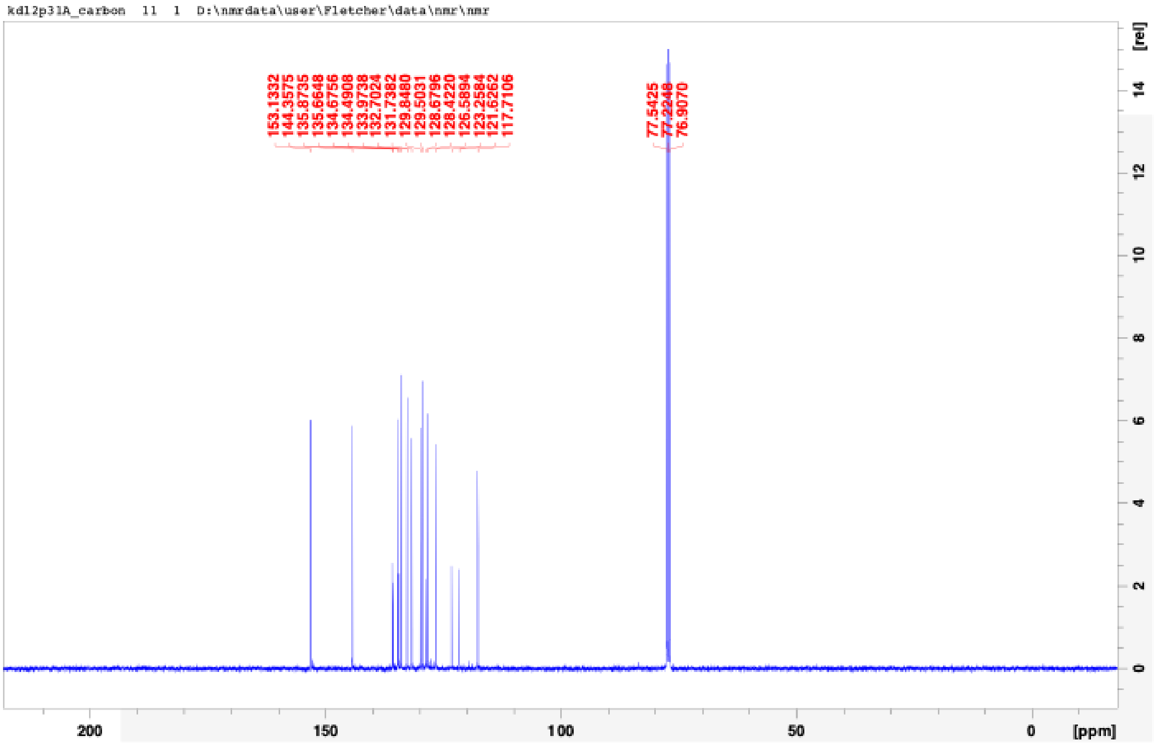

1-(2-Bromophenyl)-5-(8-isoquinolinyl)-1*H*-1,2,3-triazole (**12**)

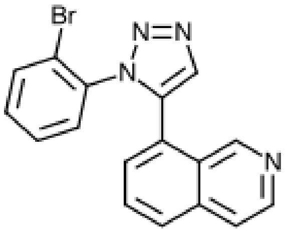

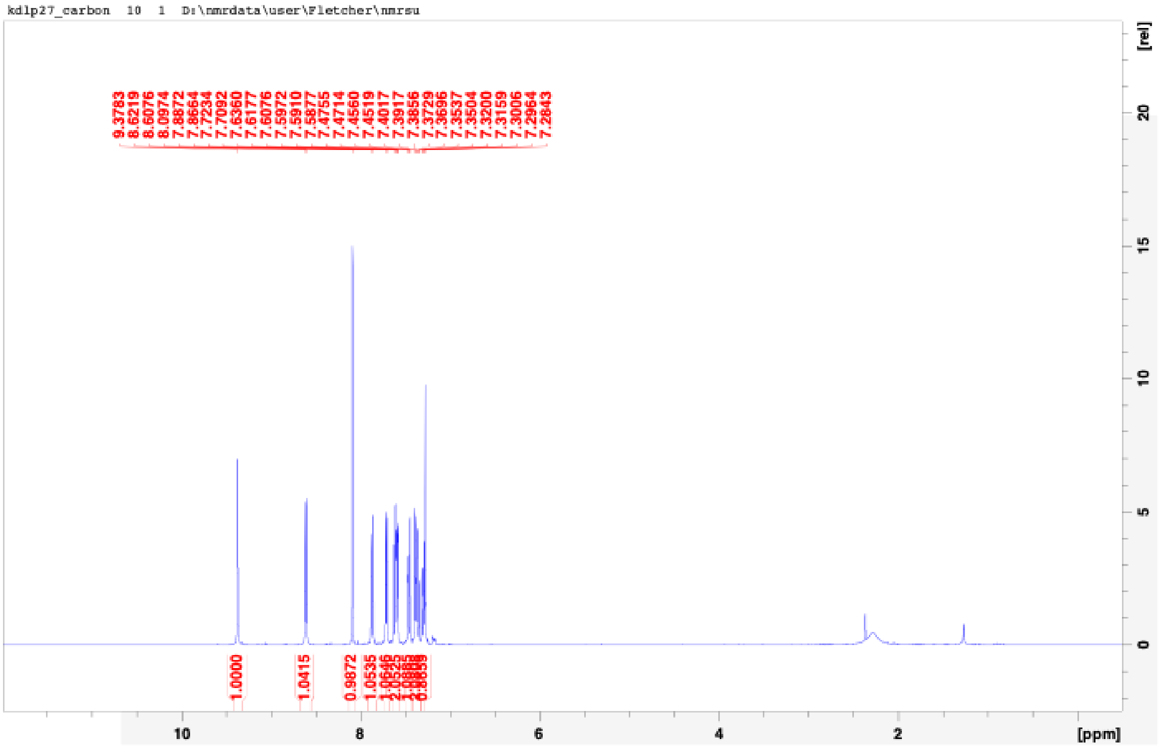

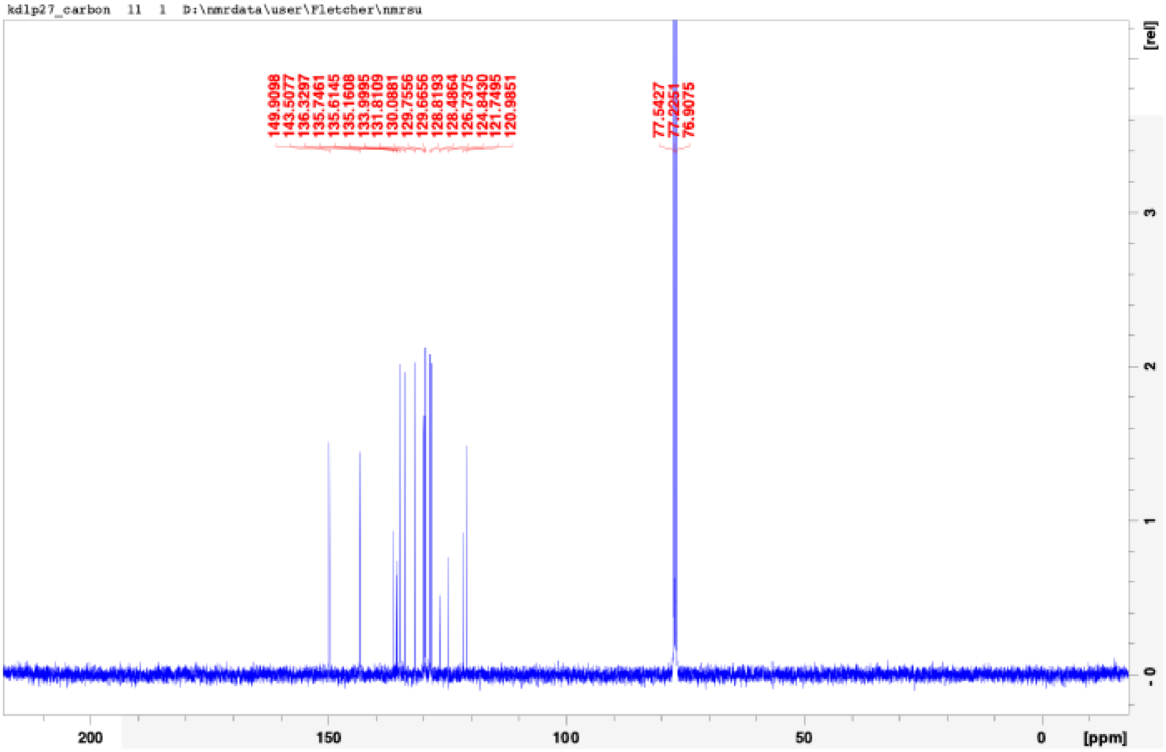

Benzo[*i*][1,2,3]triazolo[1,5-*f*]phenanthridine (**13**)

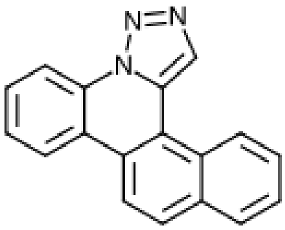

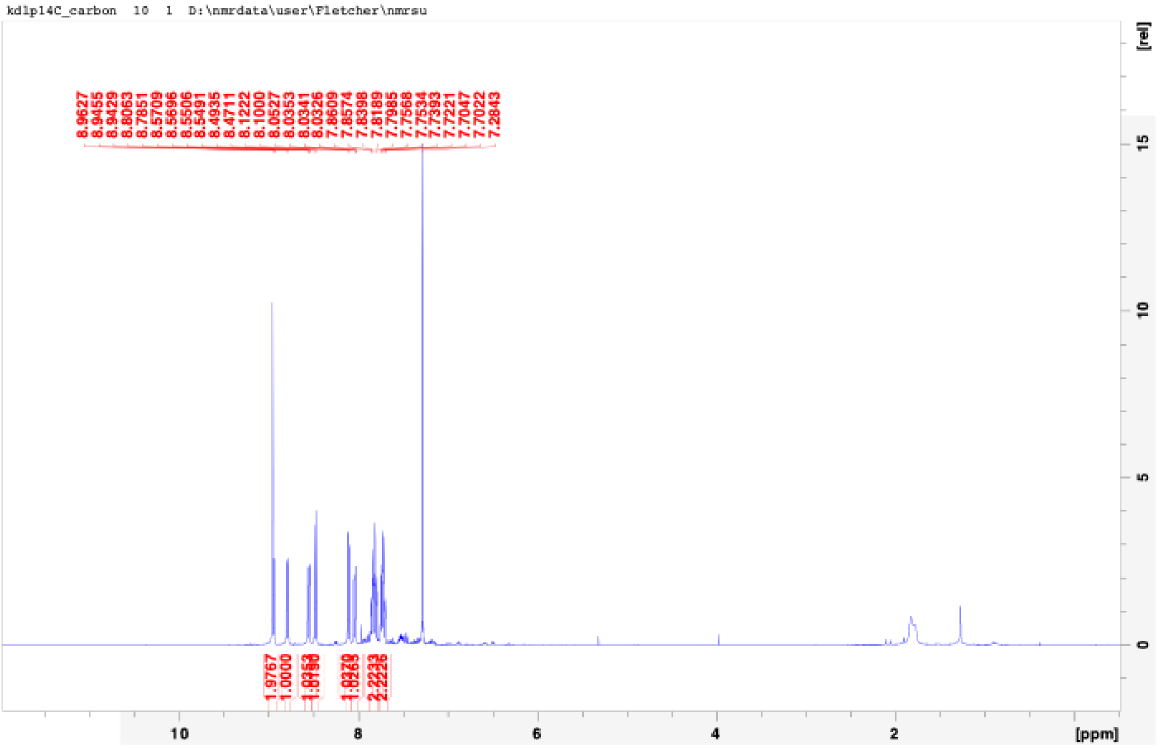

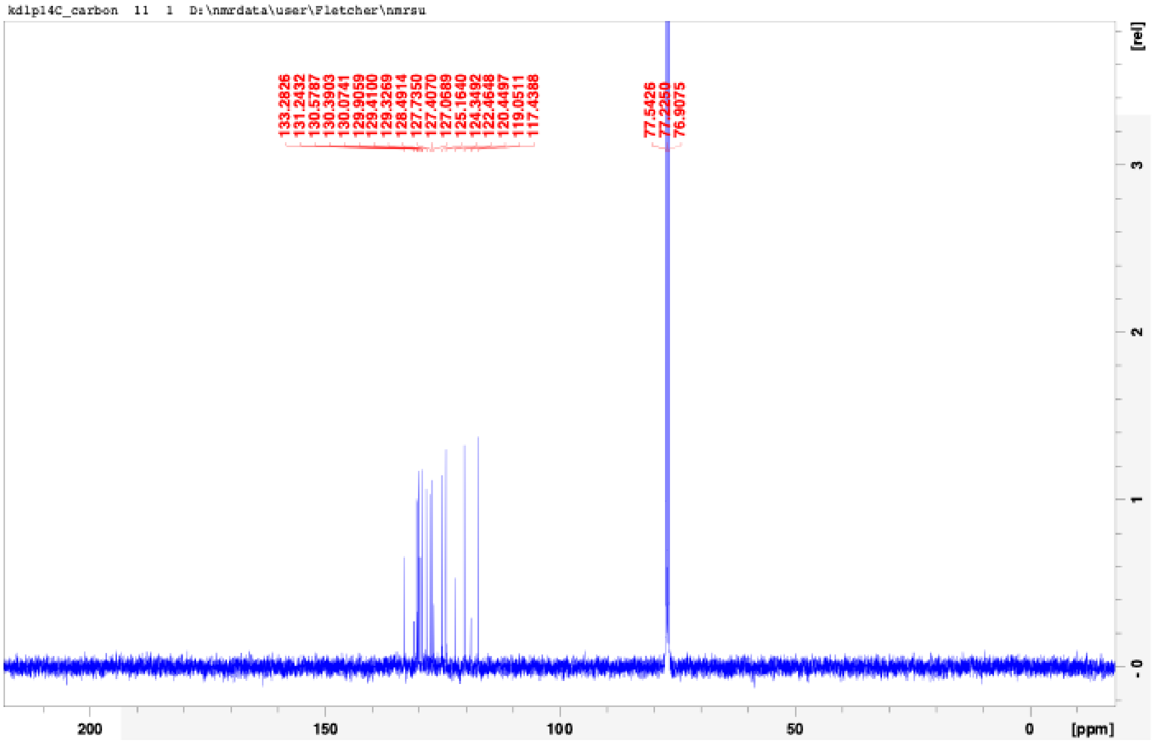

Dibenzo[*c*,*h*][1,2,3]triazolo[1,5-*a*][2,5]naphthyridine (**14**)

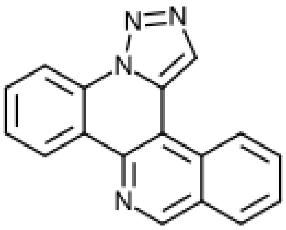

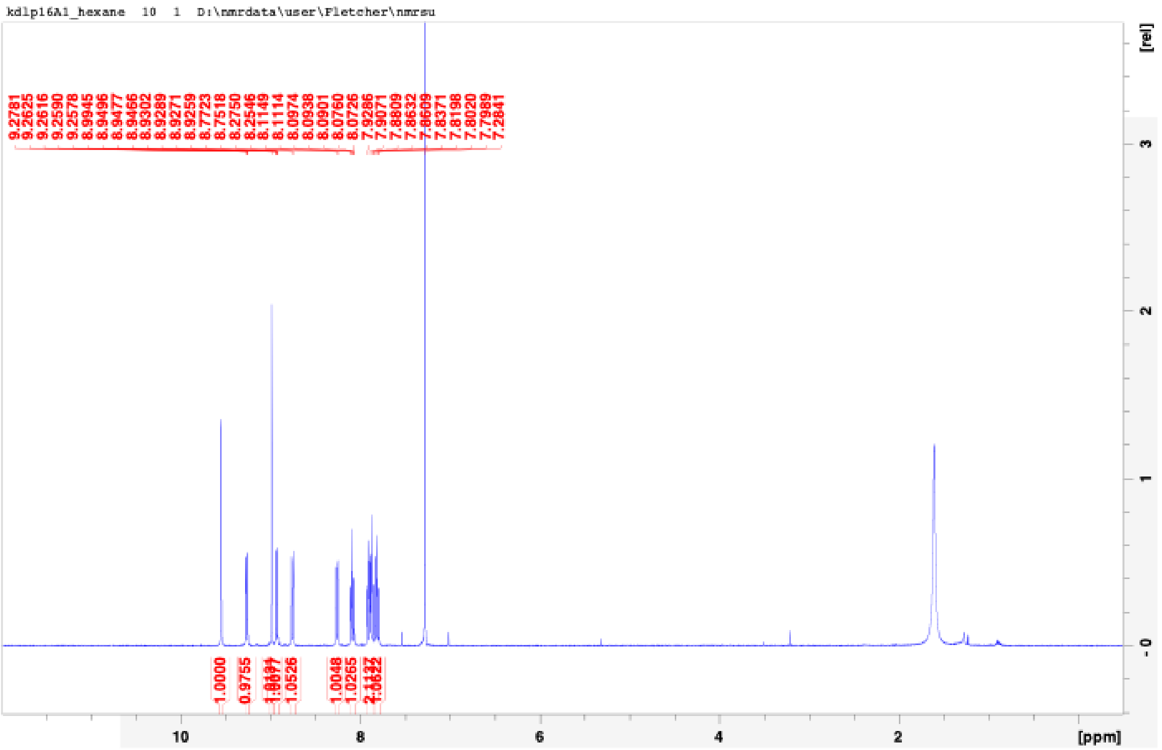

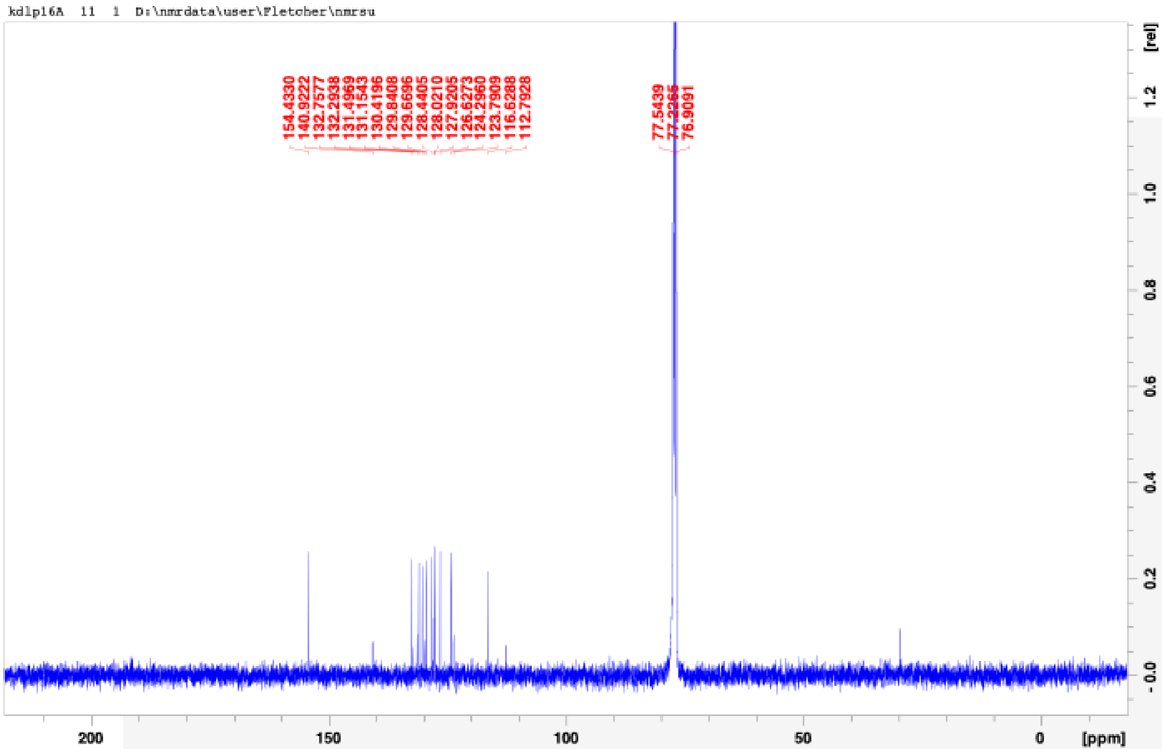

Dibenzo[*c*,*h*][1,2,3]triazolo[1,5-*a*][2,6]naphthyridine (**15**)

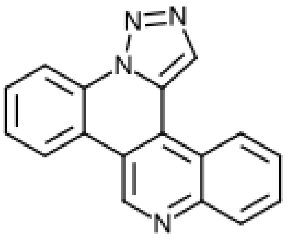

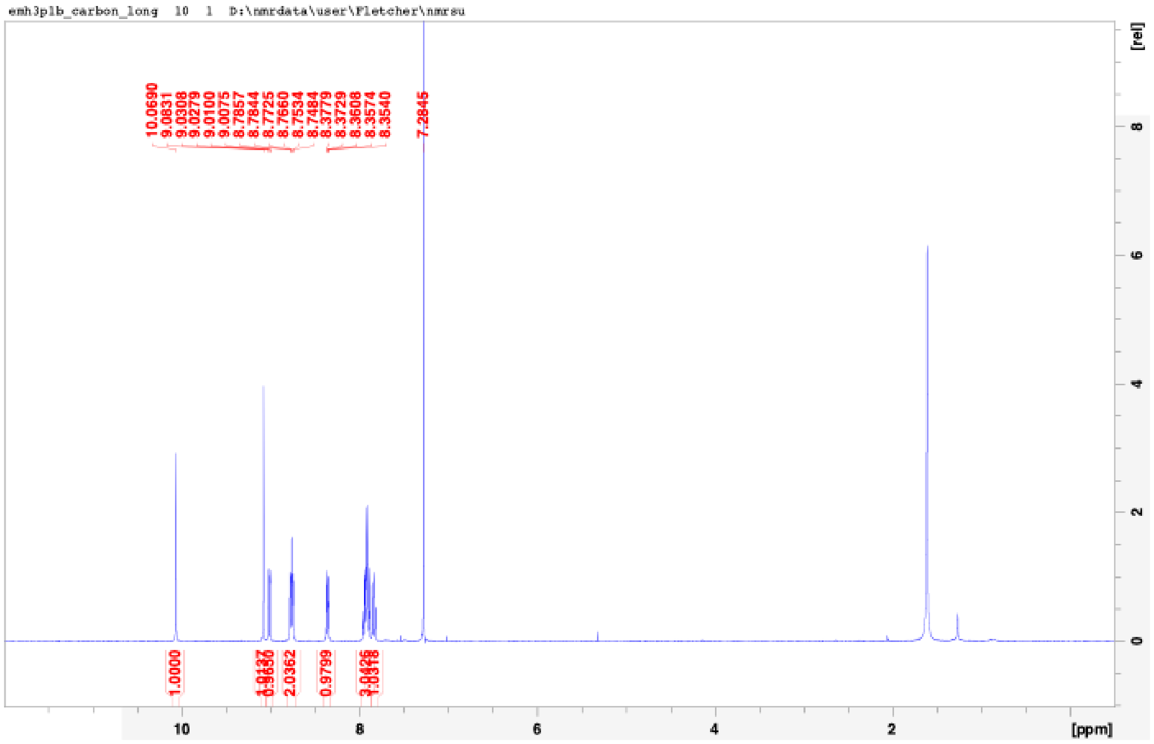

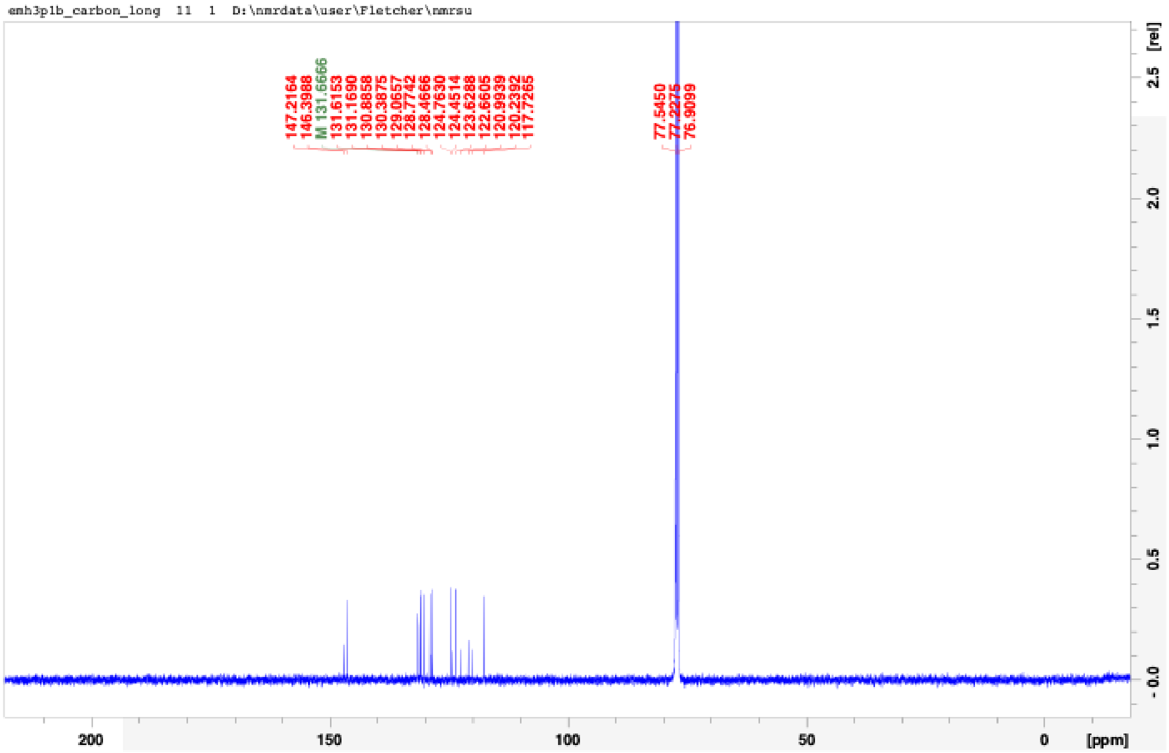

Benzo[*c*][1,2,3]triazolo[1,5-*a*][2,7]phenanthroline (**16**)

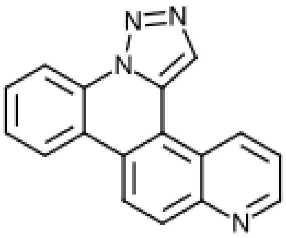

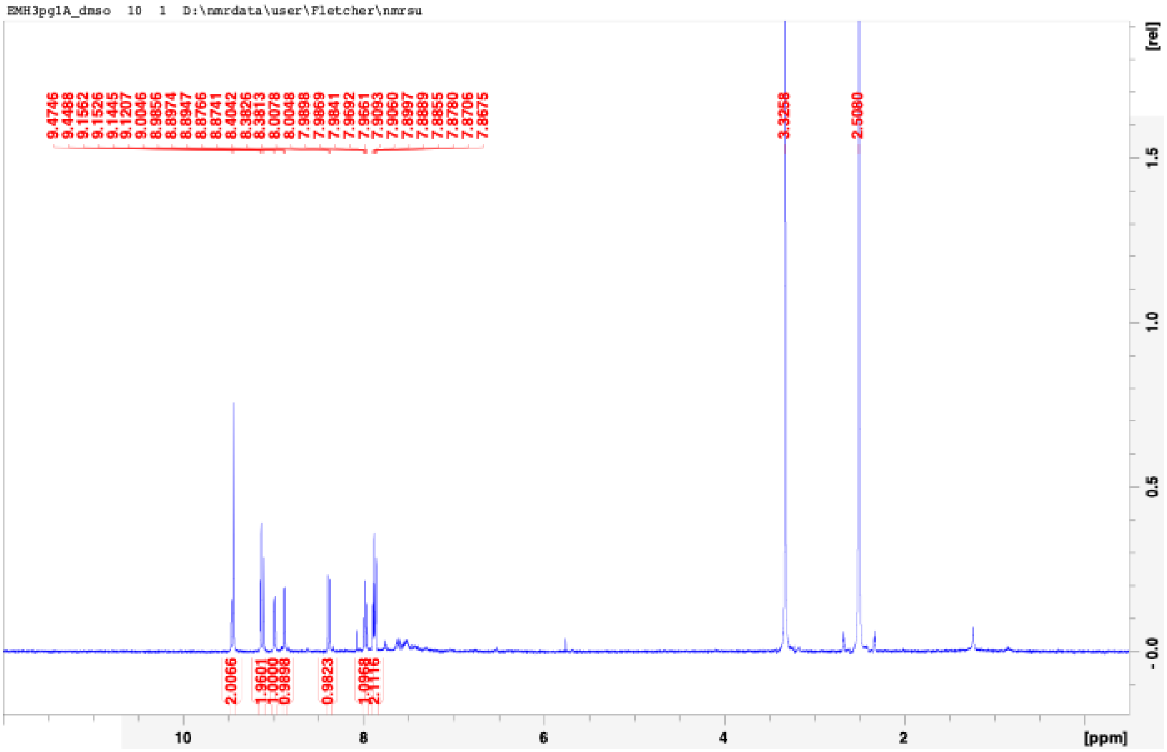

^{13}C NMR: Insufficiently soluble

Benzo[*c*][1,2,3]triazolo[1,5-*a*][2,8]phenanthroline (**17**)

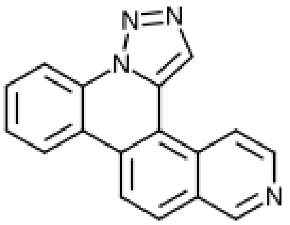

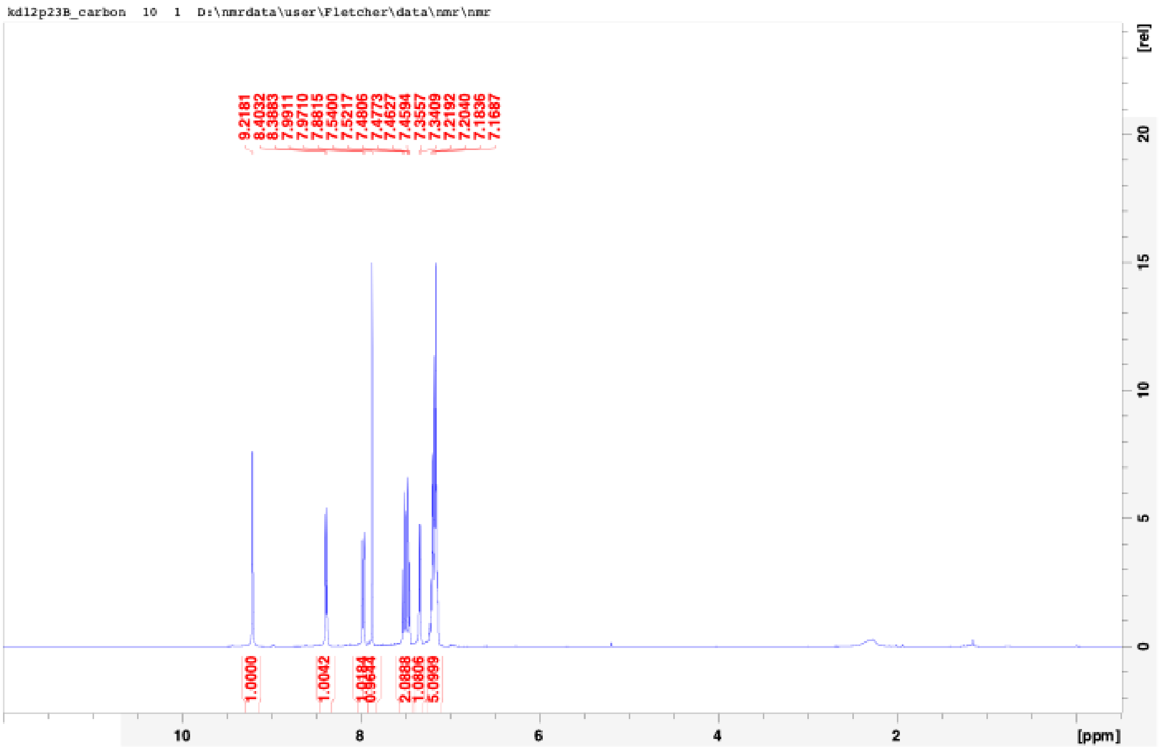

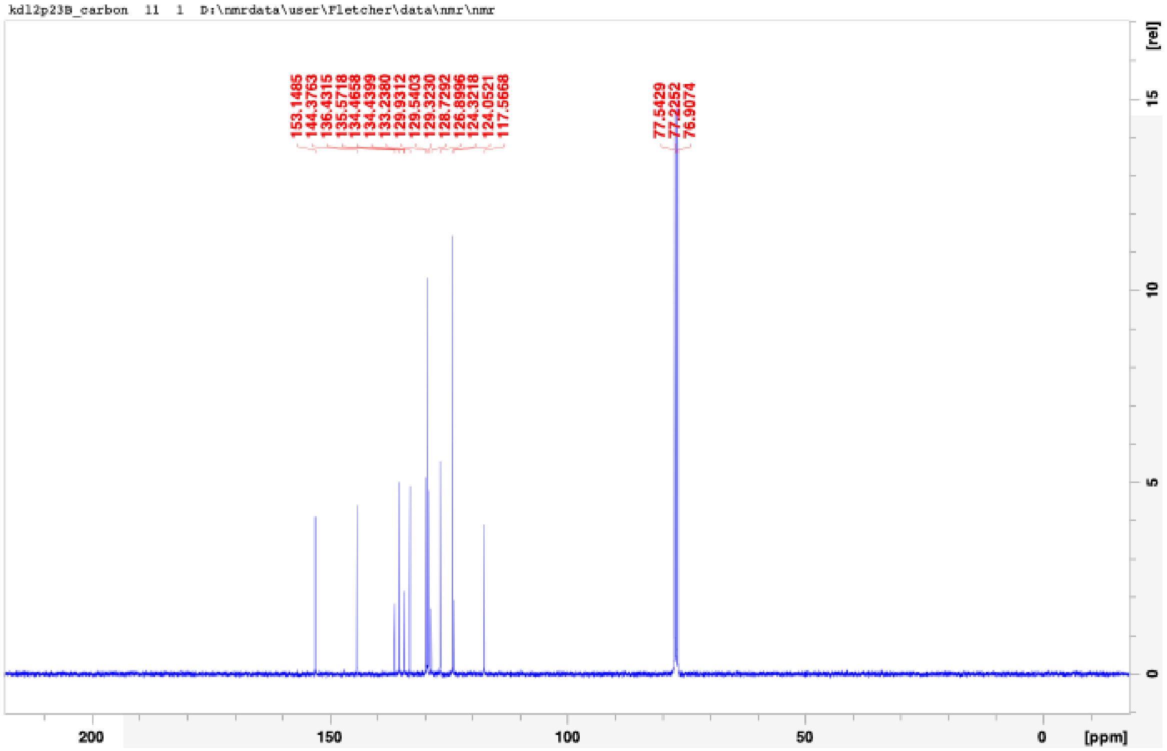

Benzo[*c*][1,2,3]triazolo[1,5-*a*][2,9]phenanthroline (**18**)

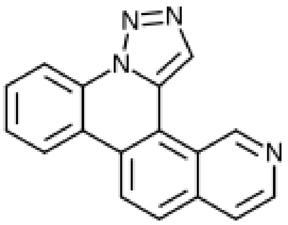

(Marginally soluble in $CDCl_3$; CNMR = 12000 scans)

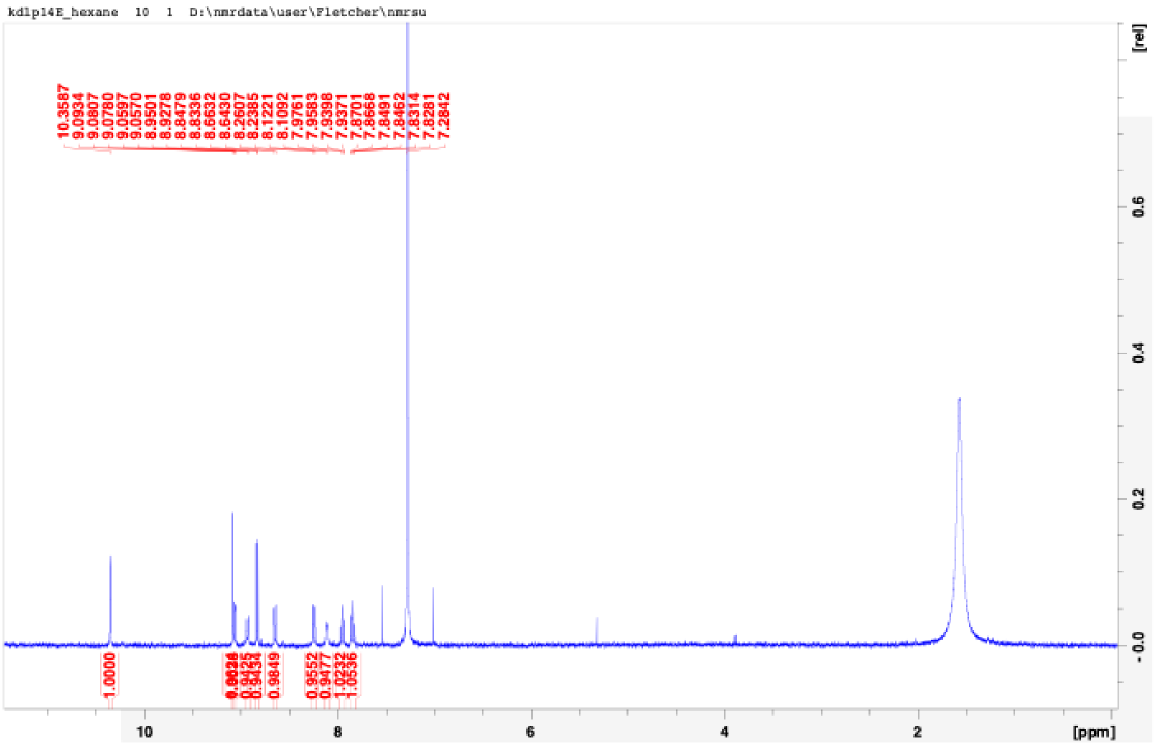

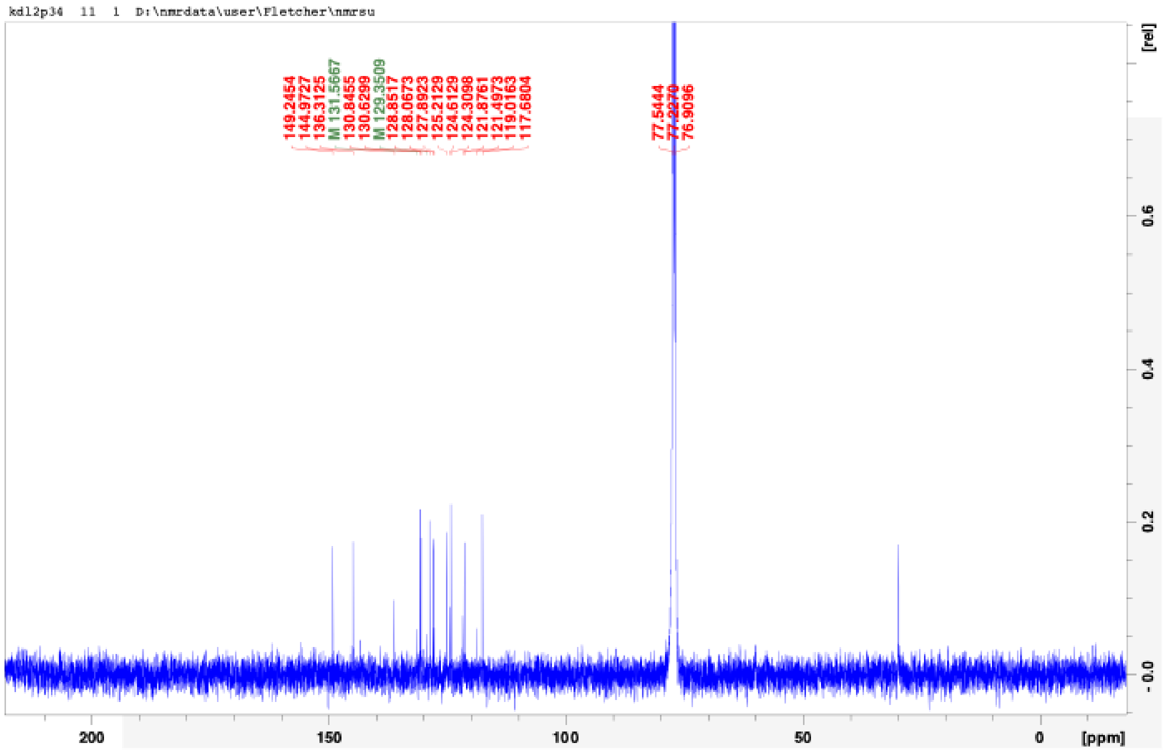

5-(2-Bromophenyl)-1-(1-naphthalenyl)-1*H*-1,2,3-triazole (**25**)

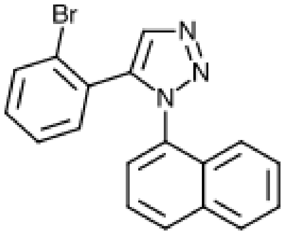

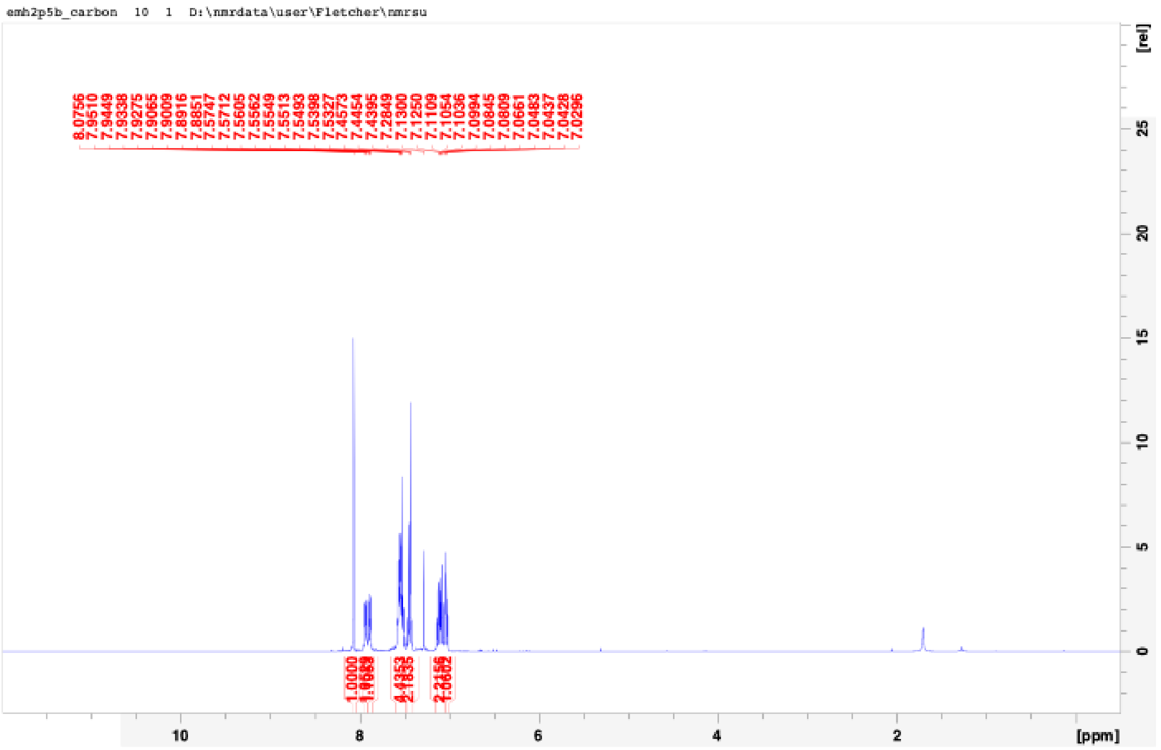

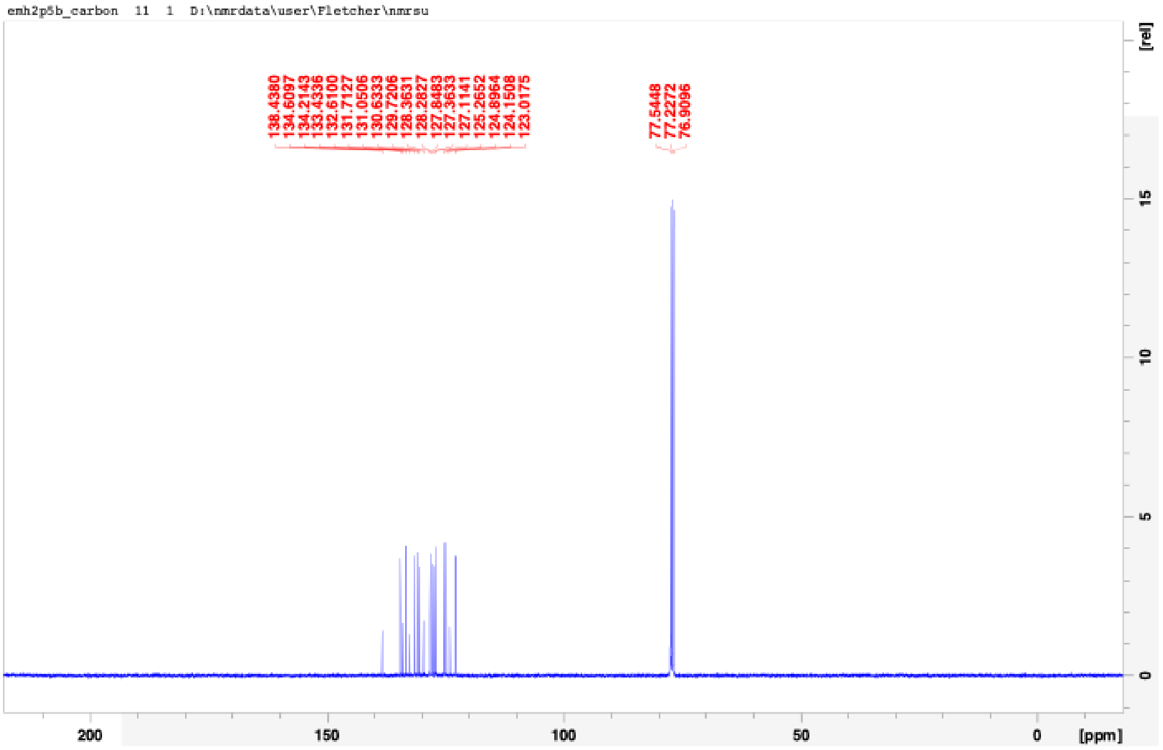

5-(2-Bromophenyl)-1-(4-isoquinolinyl)-1*H*-1,2,3-triazole (**26**)

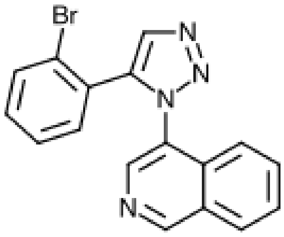

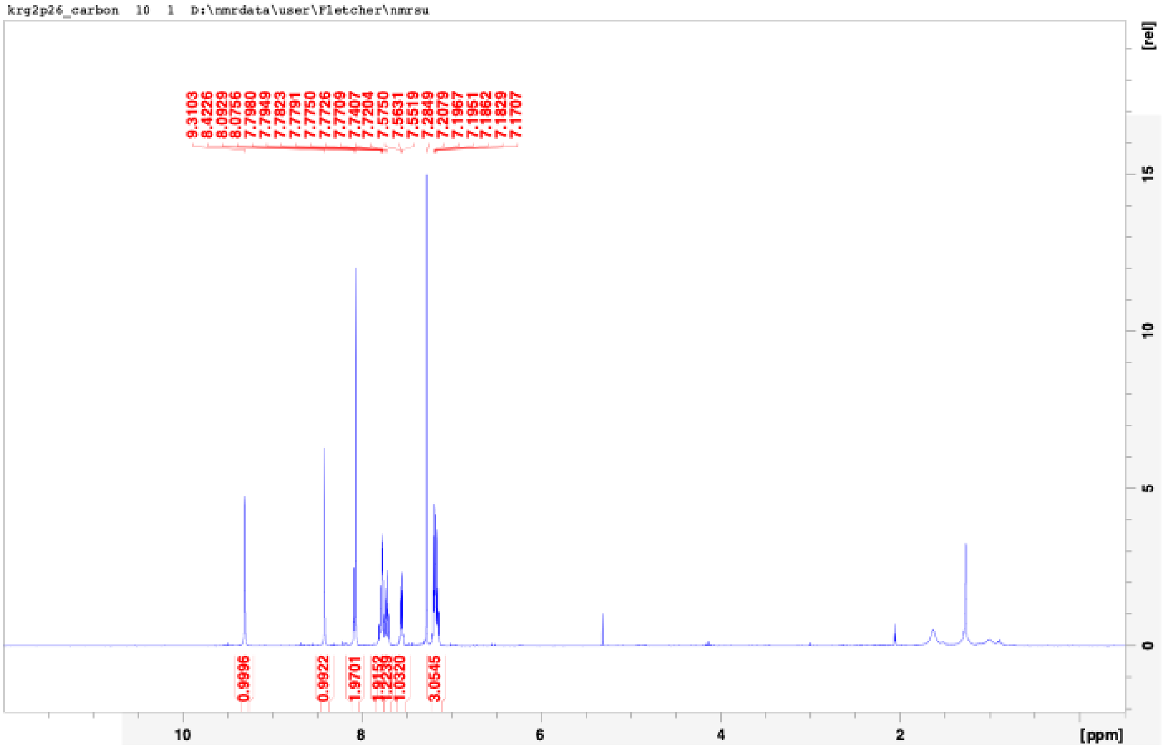

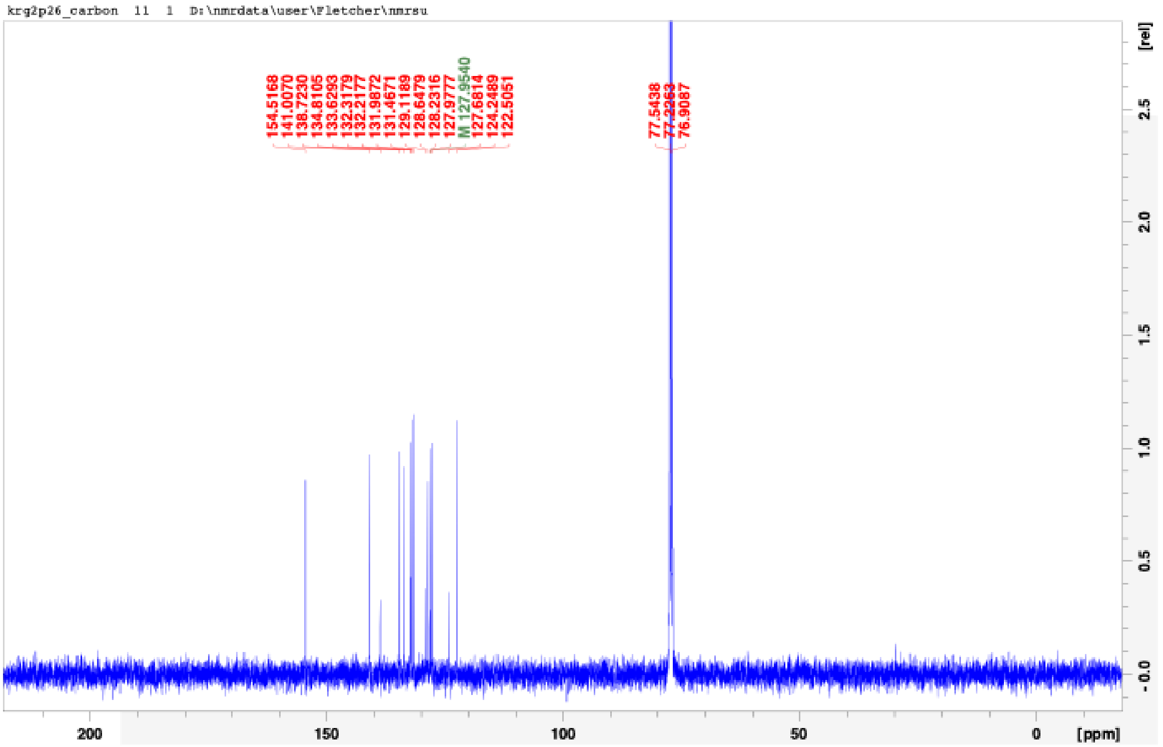

5-(2-Bromophenyl)-1-(4-quinolinyl)-1*H*-1,2,3-triazole (**27**)

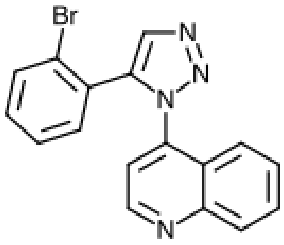

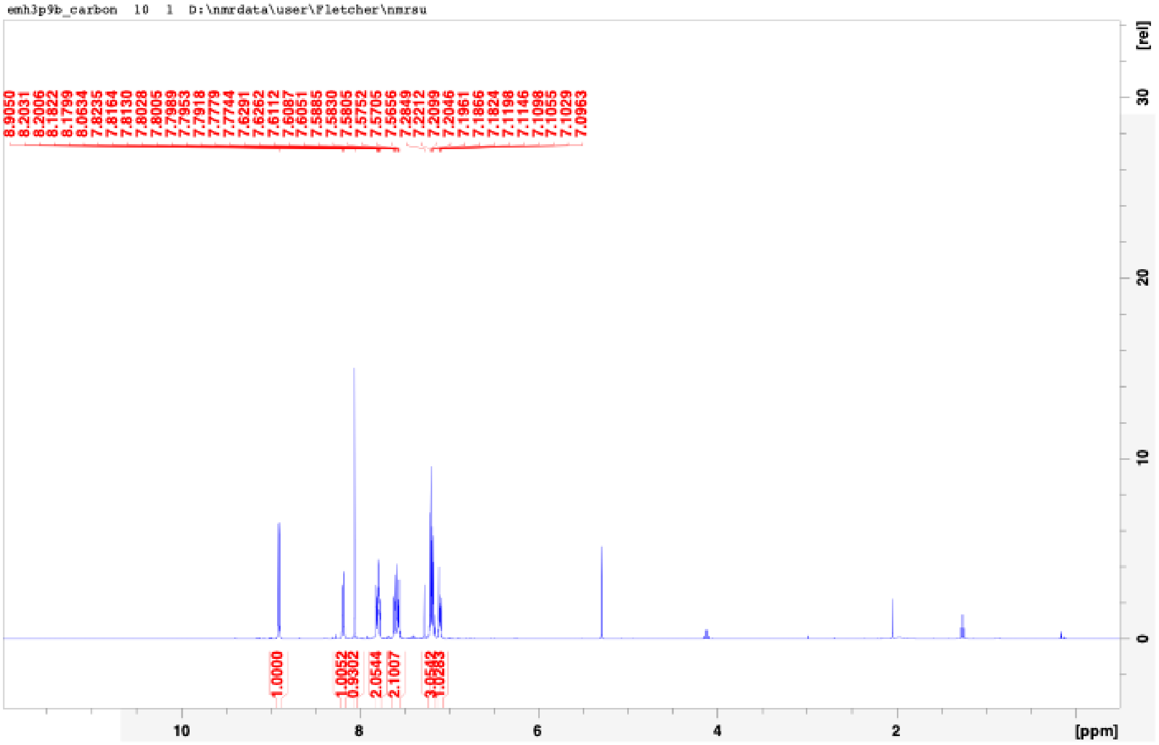

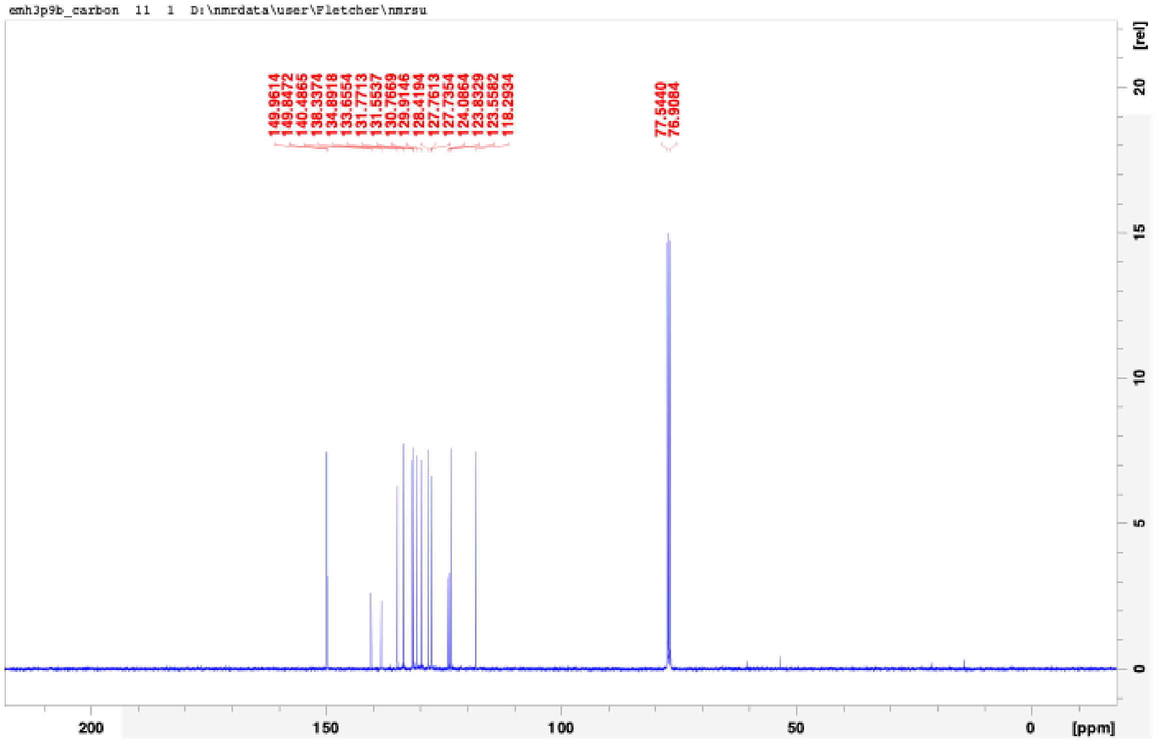

5-(2-Bromophenyl)-1-(5-quinolinyl)-1*H*-1,2,3-triazole (**28**)

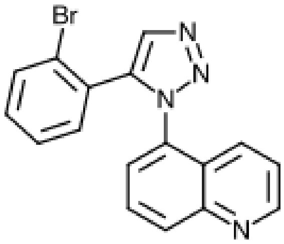

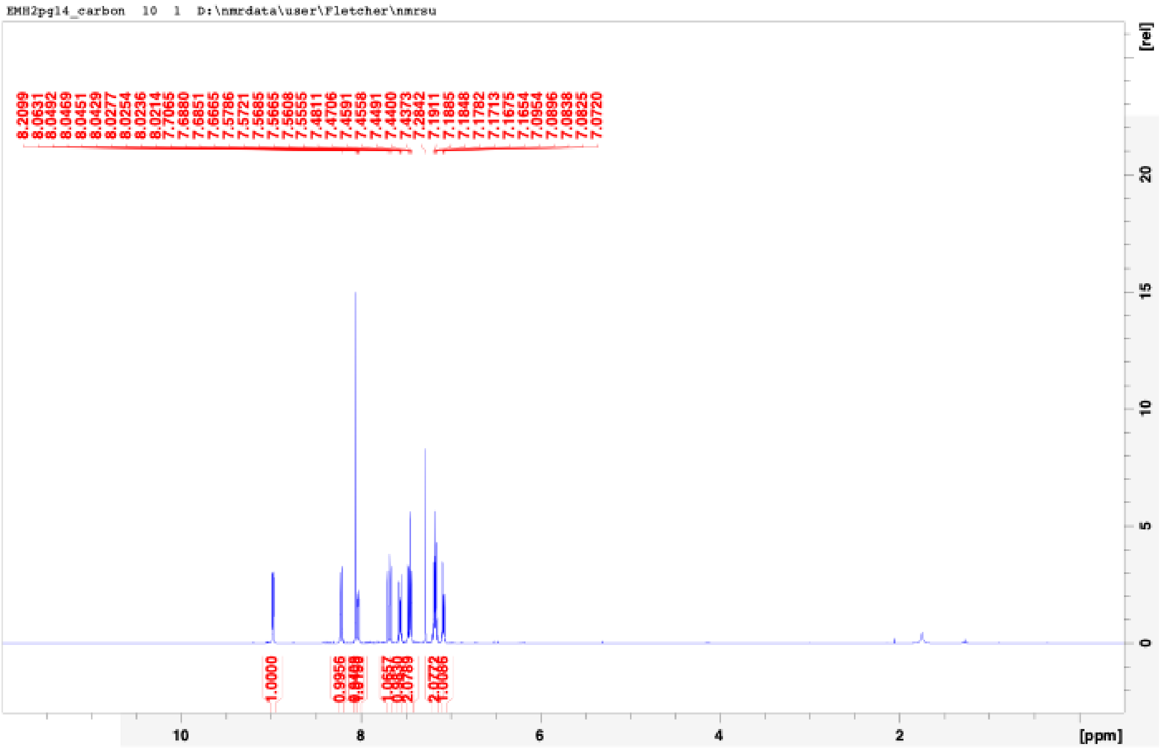

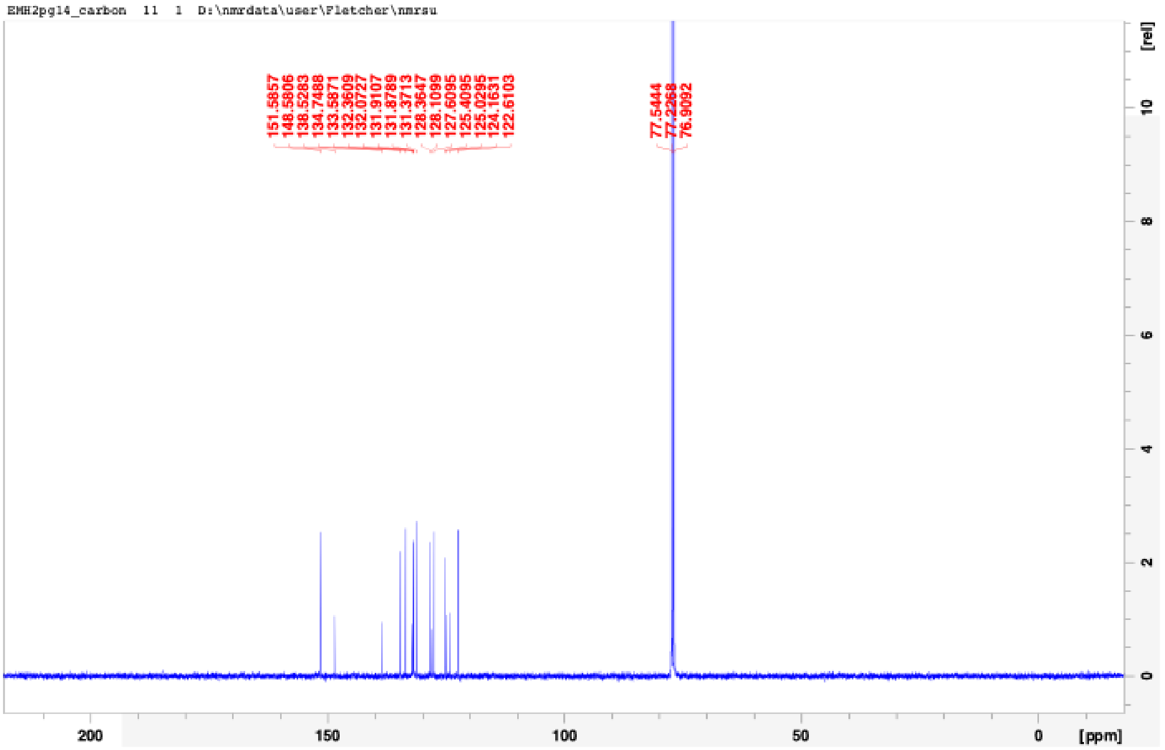

5-(2-Bromophenyl)-1-(5-isoquinolinyl)-1*H*-1,2,3-triazole (**29**)

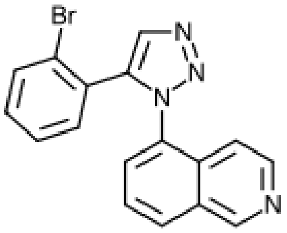

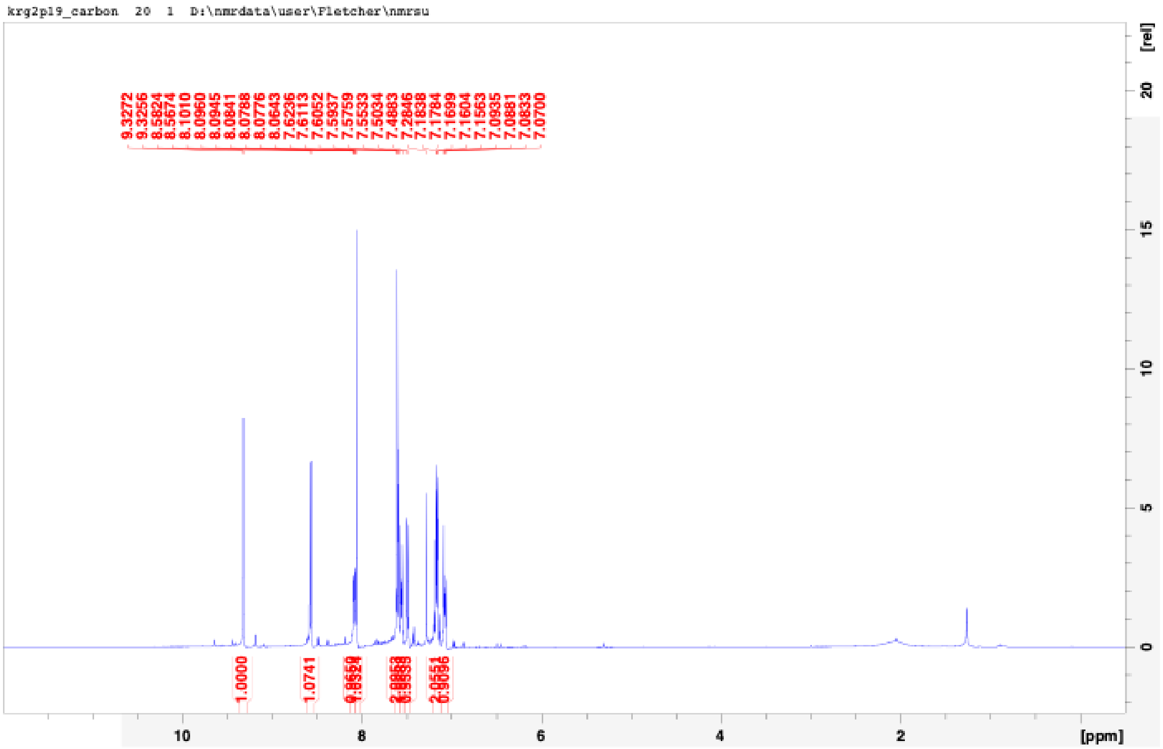

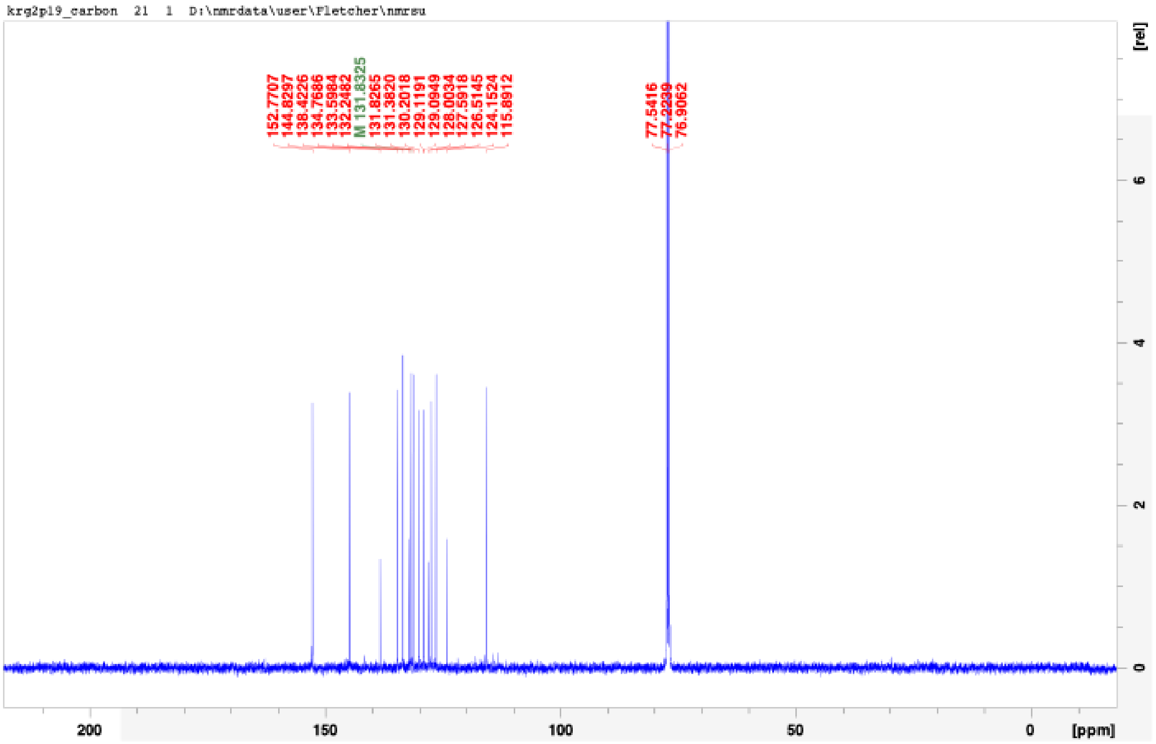

5-(2-Bromophenyl)-1-(8-isoquinolinyl)-1*H*-1,2,3-triazole (**30**)

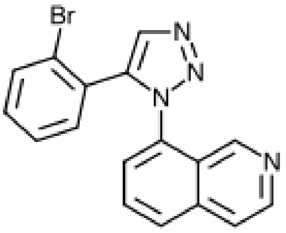

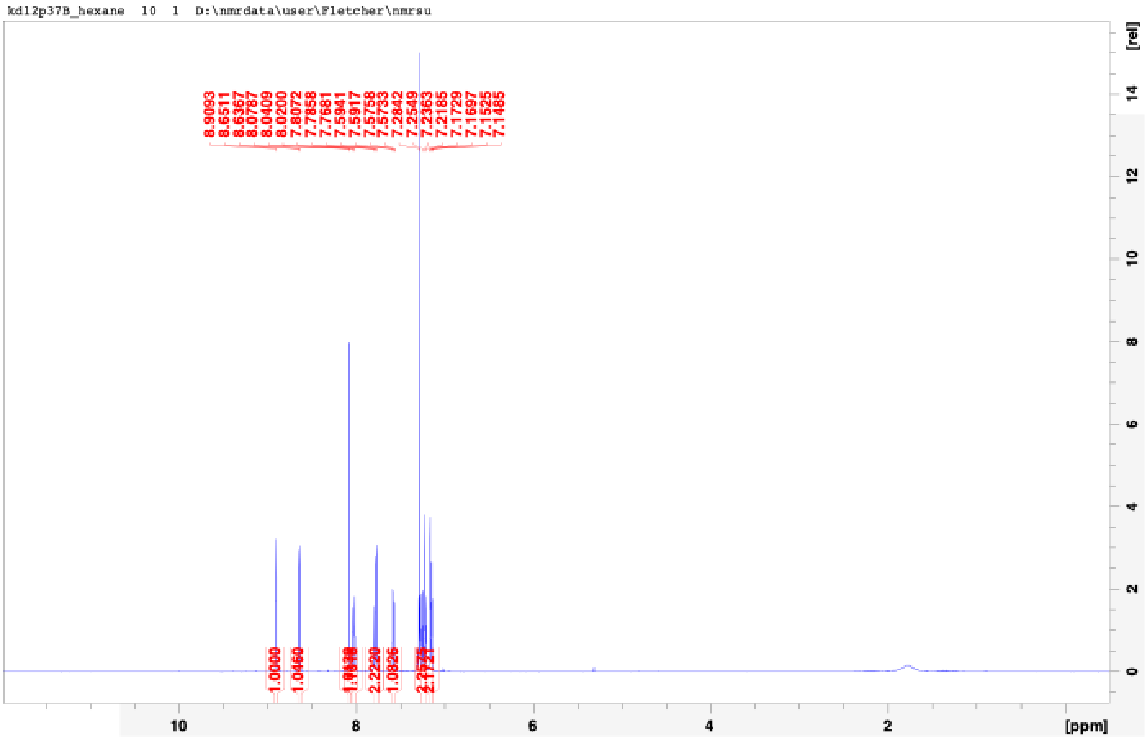

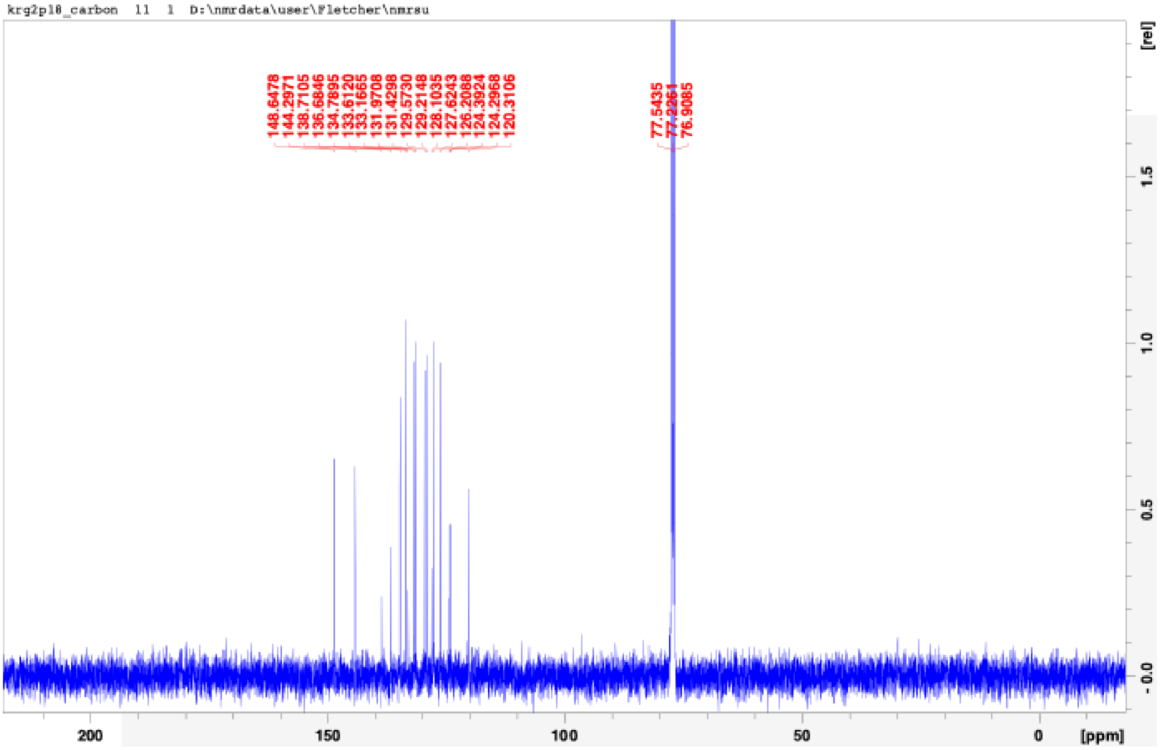

Benzo[*c*][1,2,3]triazolo[1,5-*f*]phenanthridine (**31**)

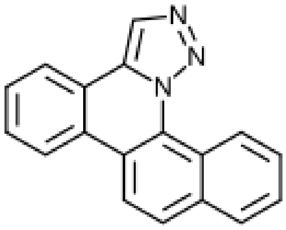

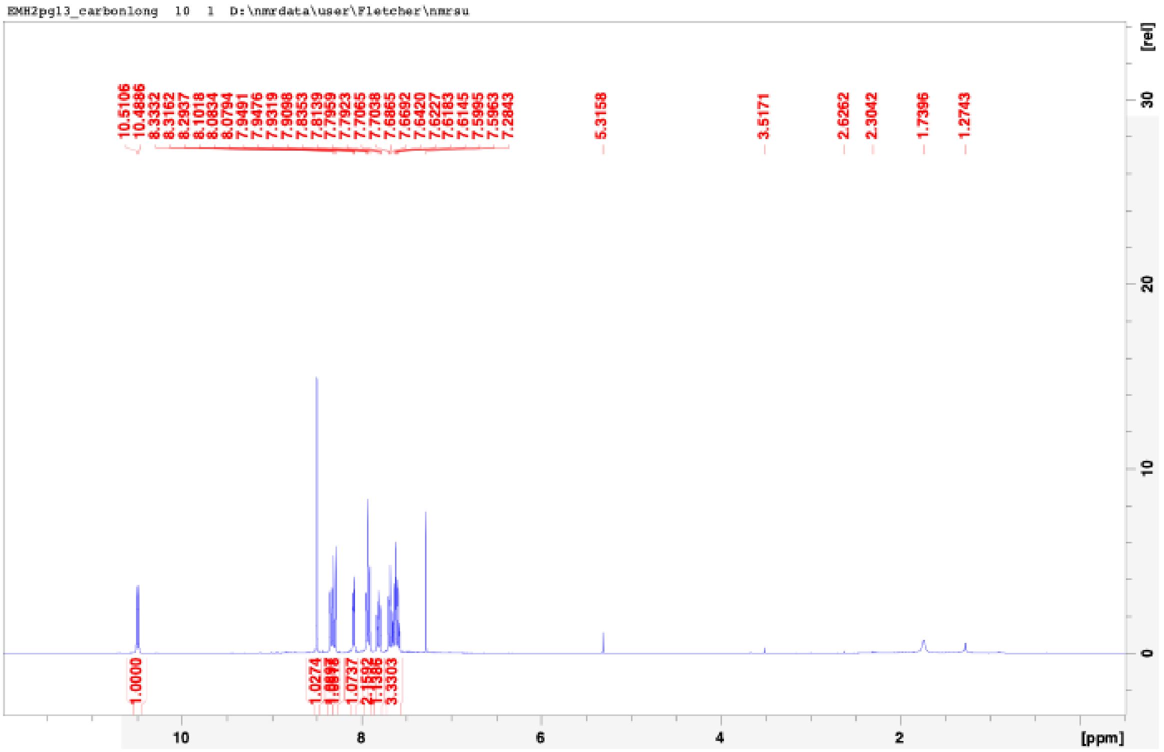

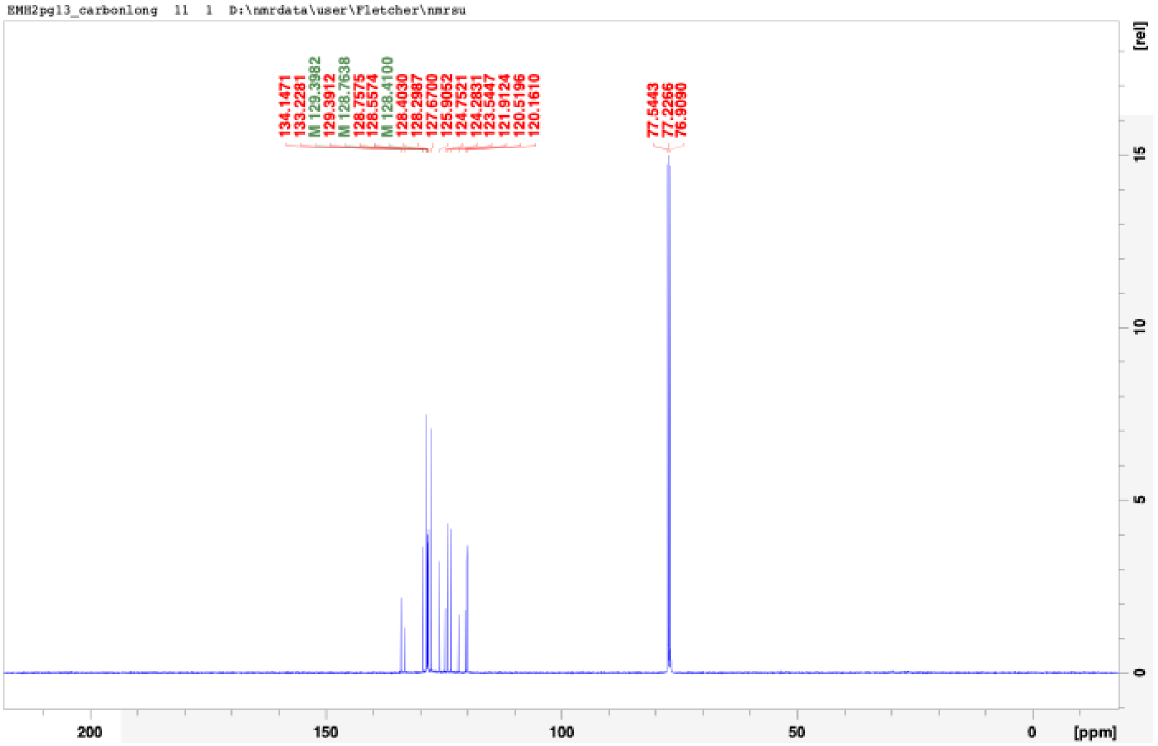

Dibenzo[*c*,*h*][1,2,3]triazolo[1,5-*a*][1,5]naphthyridine (**32**)

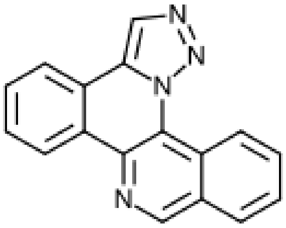

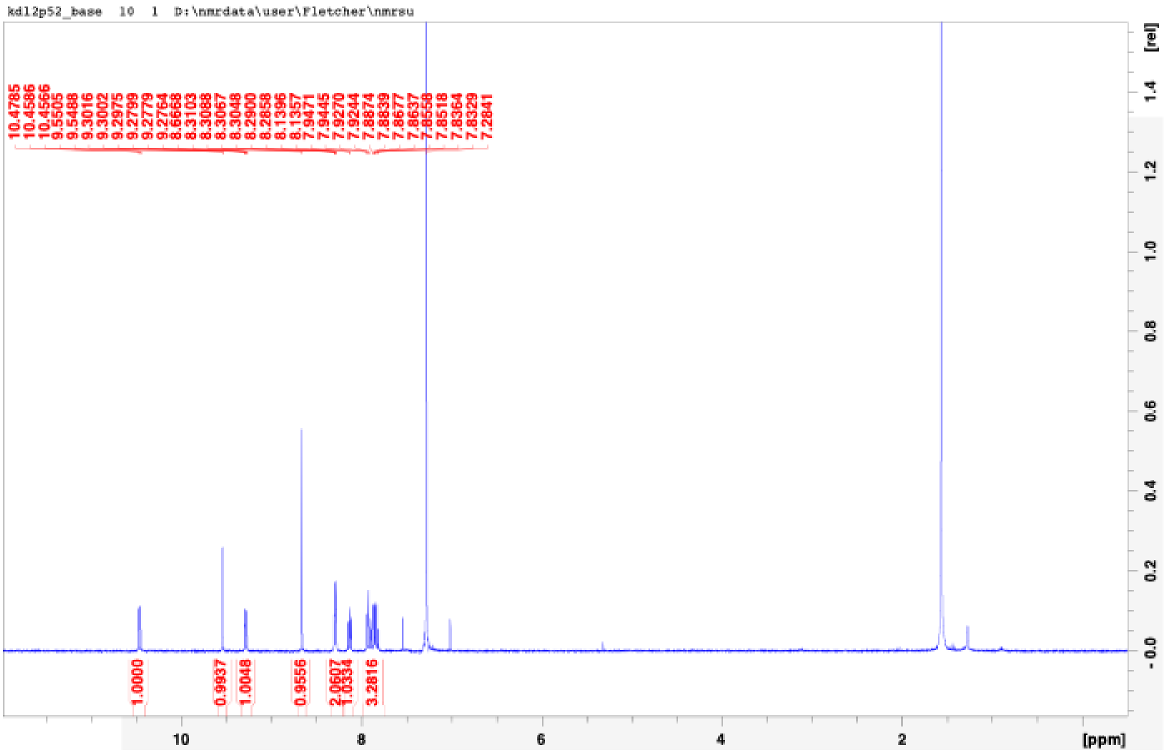

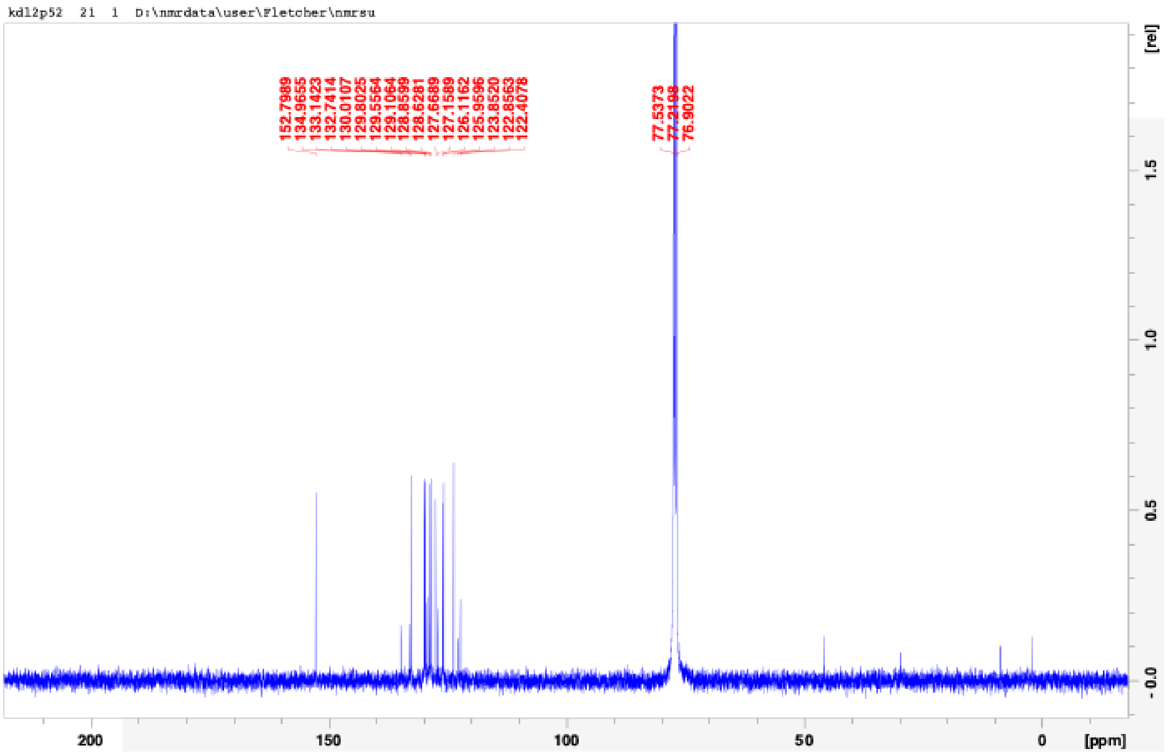

Dibenzo[*c*,*h*][1,2,3]triazolo[1,5-*a*][1,6]naphthyridine (**33**)

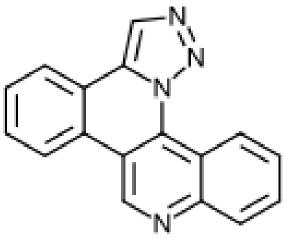

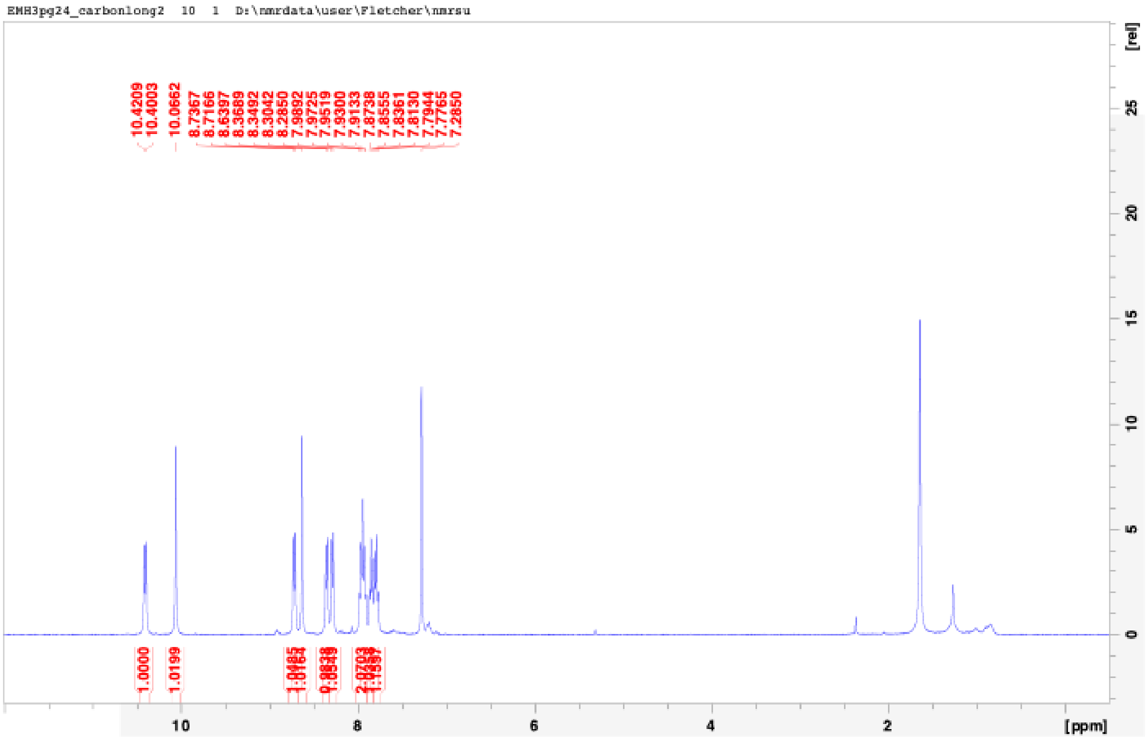

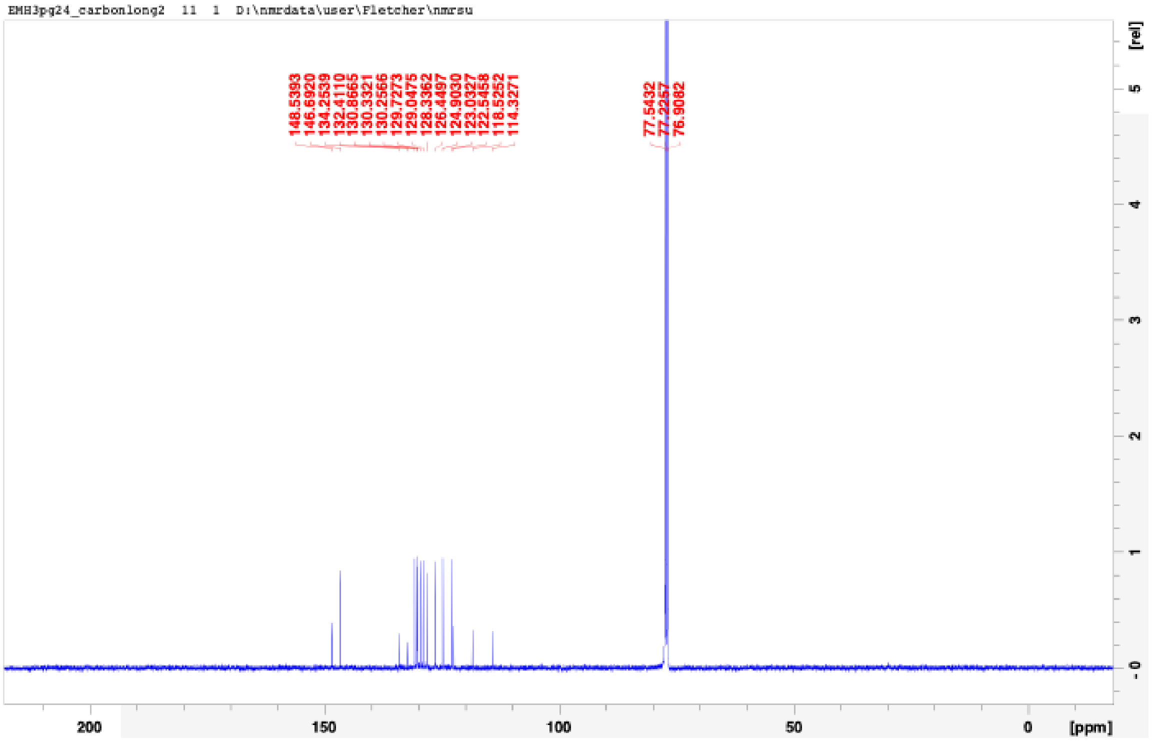

Benzo[*c*][1,2,3]triazolo[1,5-*a*][1,7]phenanthroline (**34**)

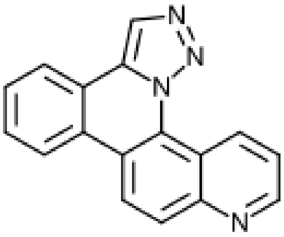

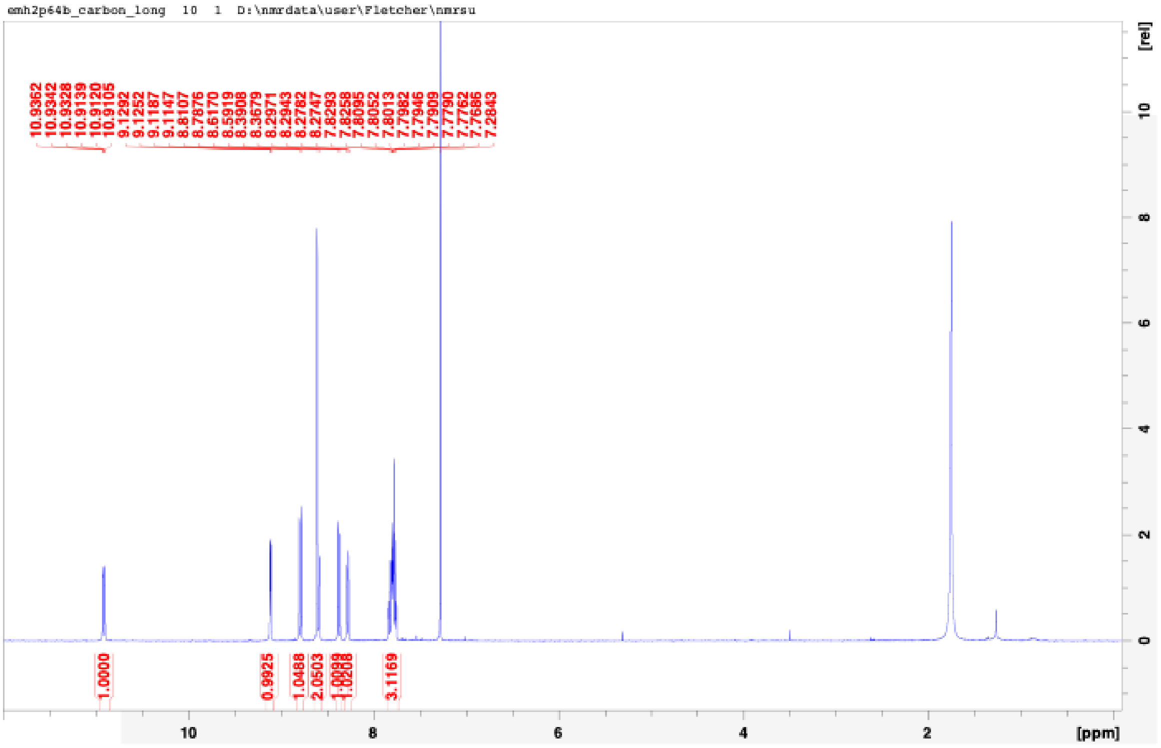

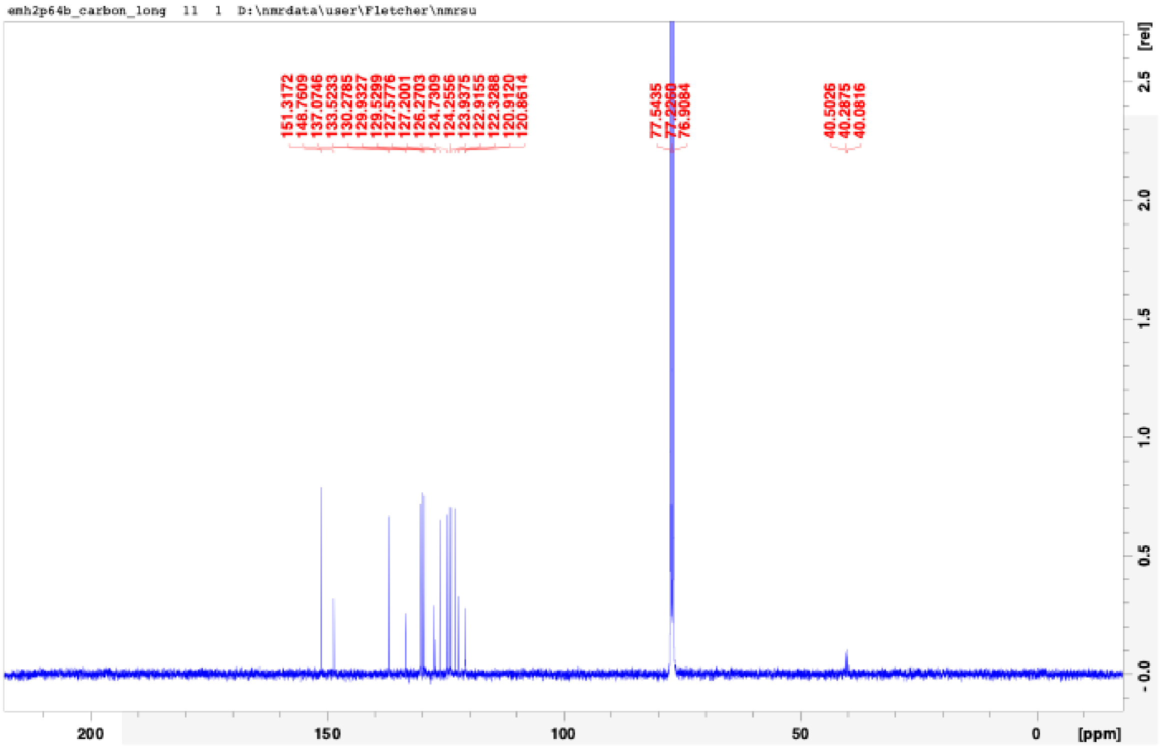

Benzo[*c*][1,2,3]triazolo[1,5-*a*][1,8]phenanthroline (**35**)

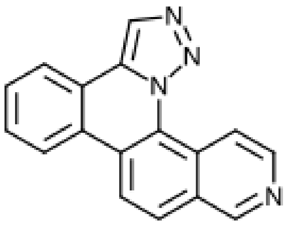

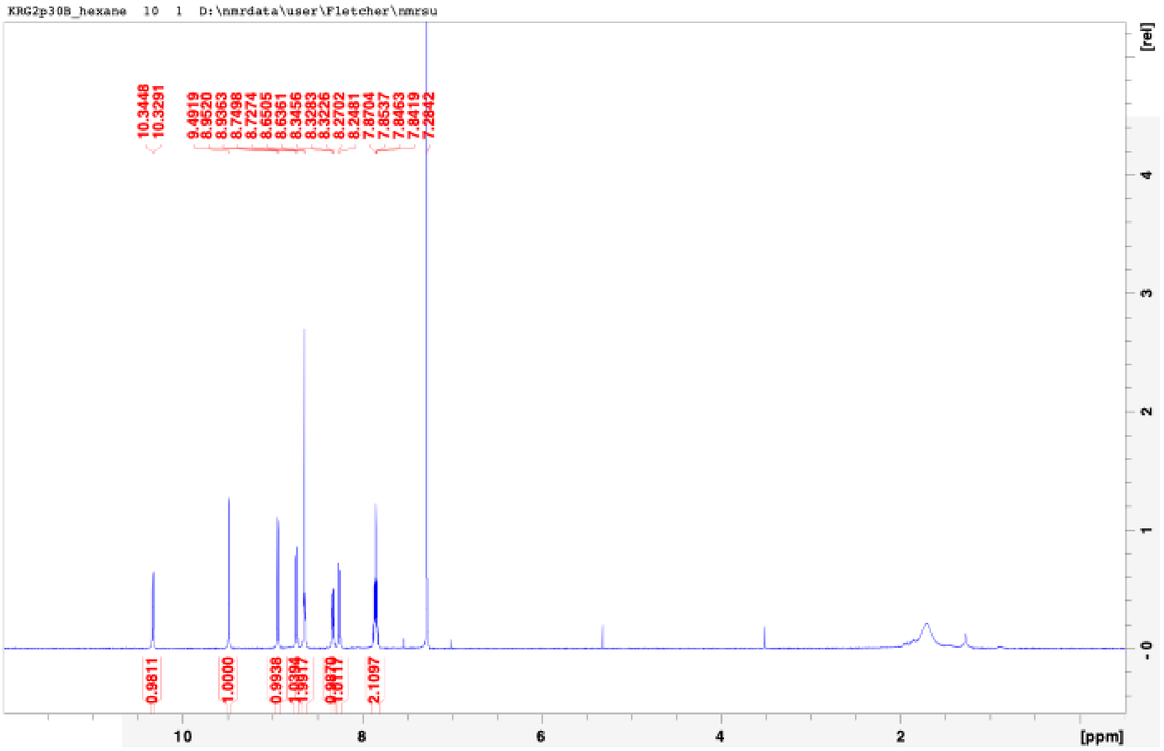

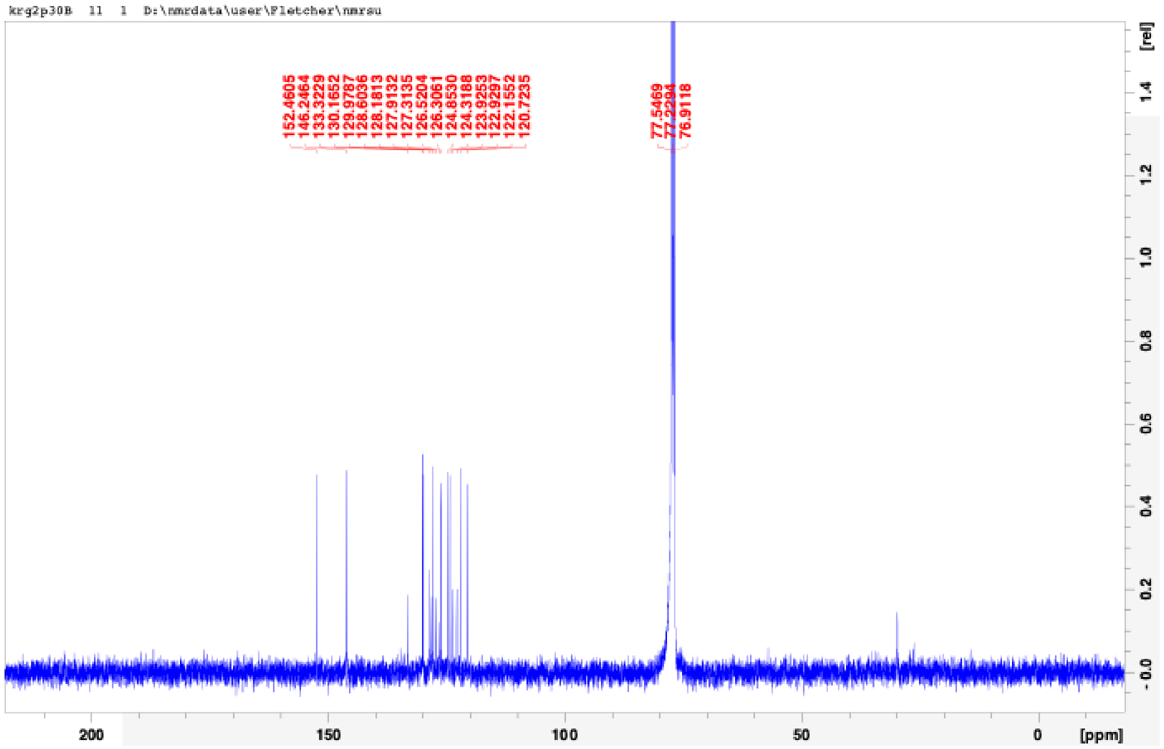

Benzo[*c*][1,2,3]triazolo[1,5-*a*][1,9]phenanthroline (**36**)

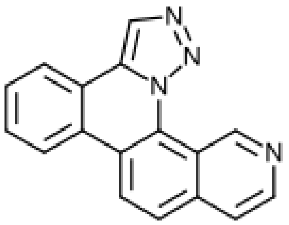

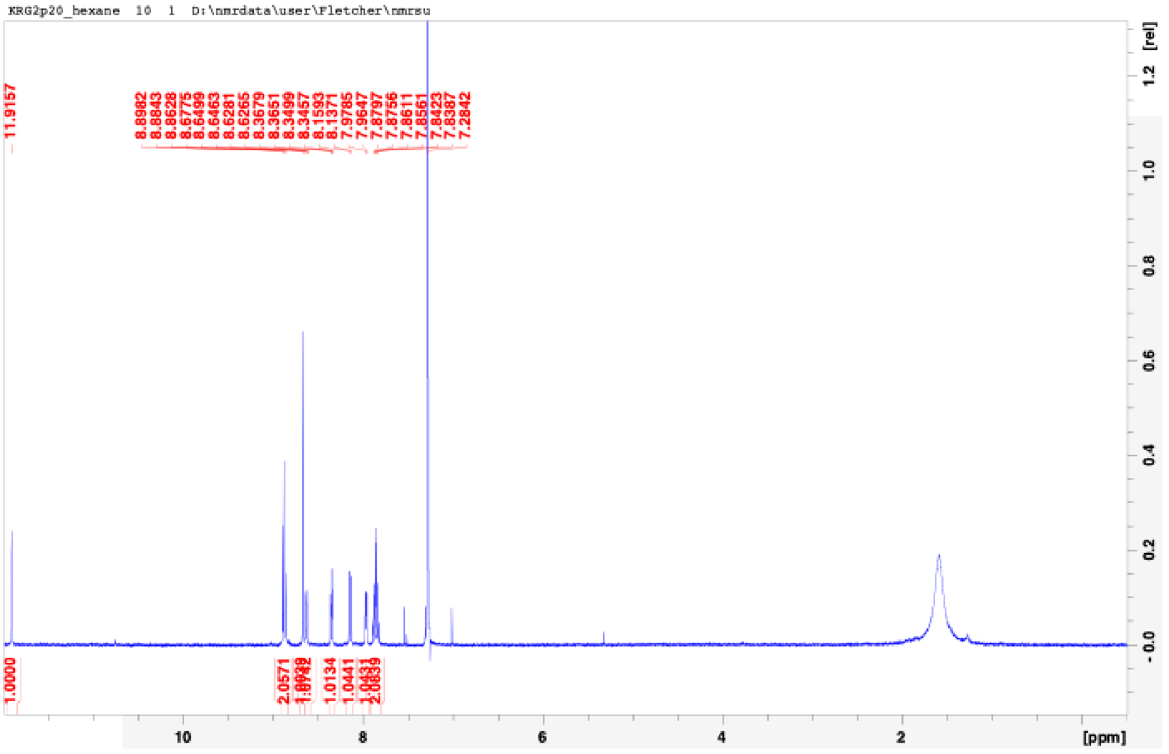

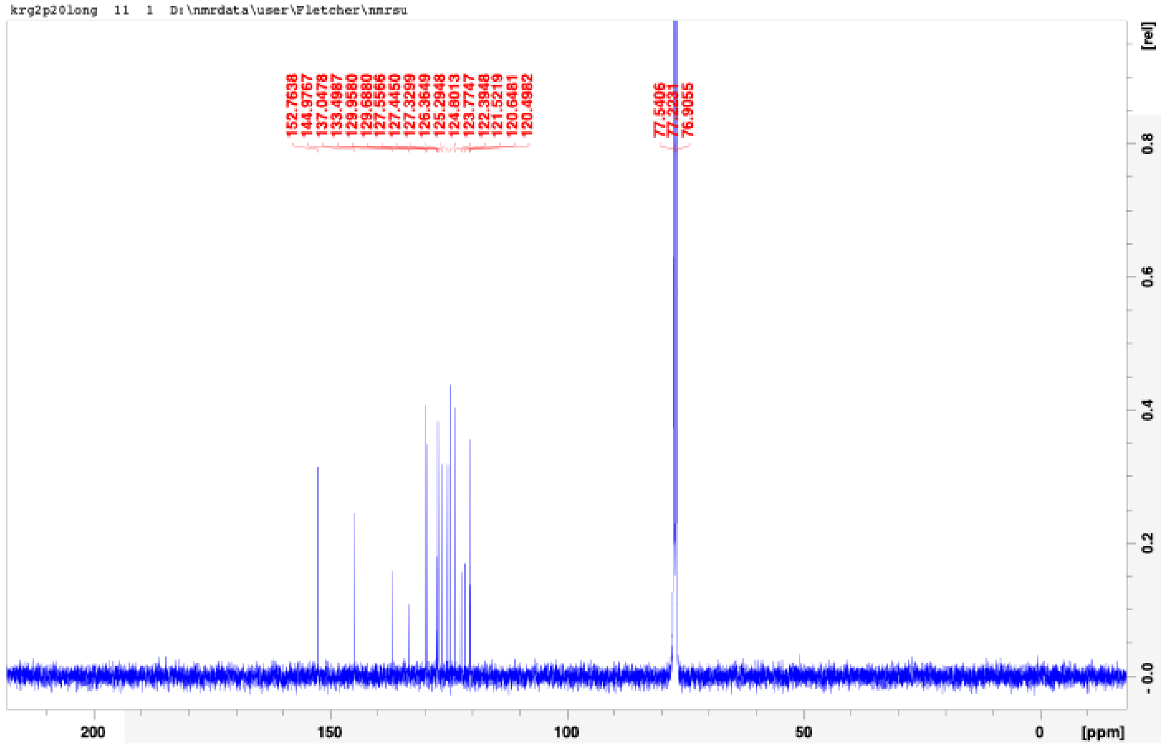

5-(1-Naphthalenyl)-1-phenyl-1*H*-1,2,3-triazole (**37**)

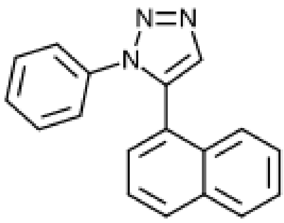

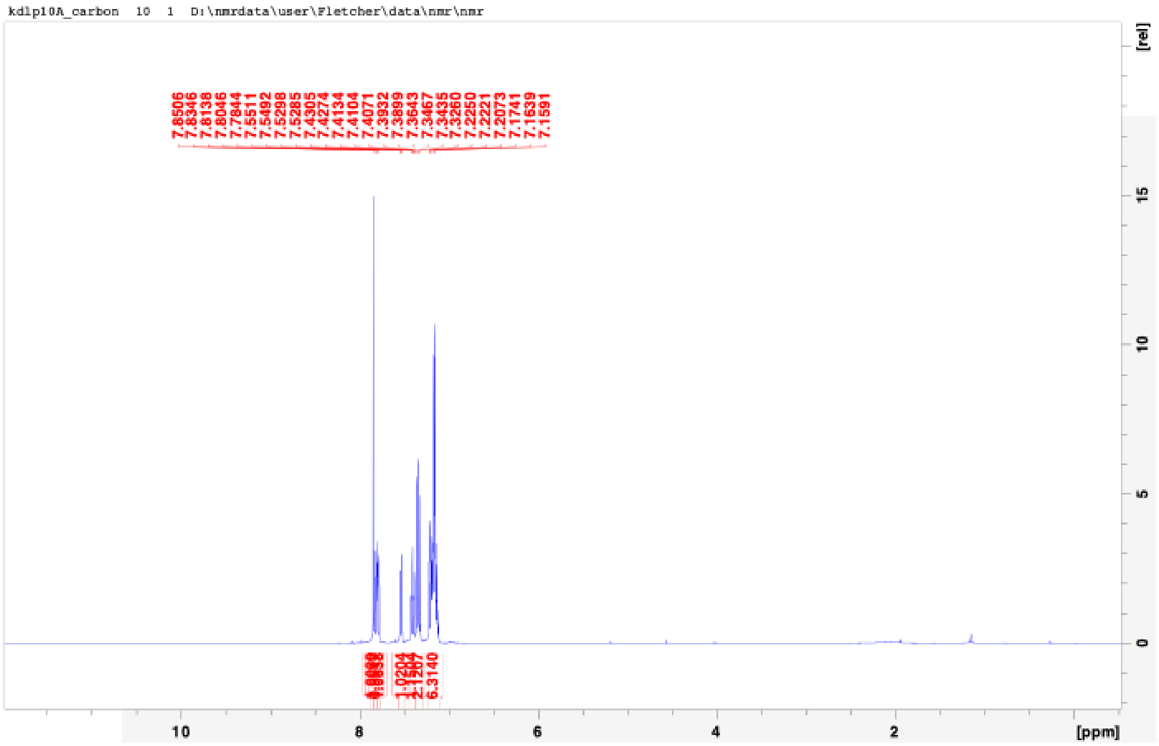

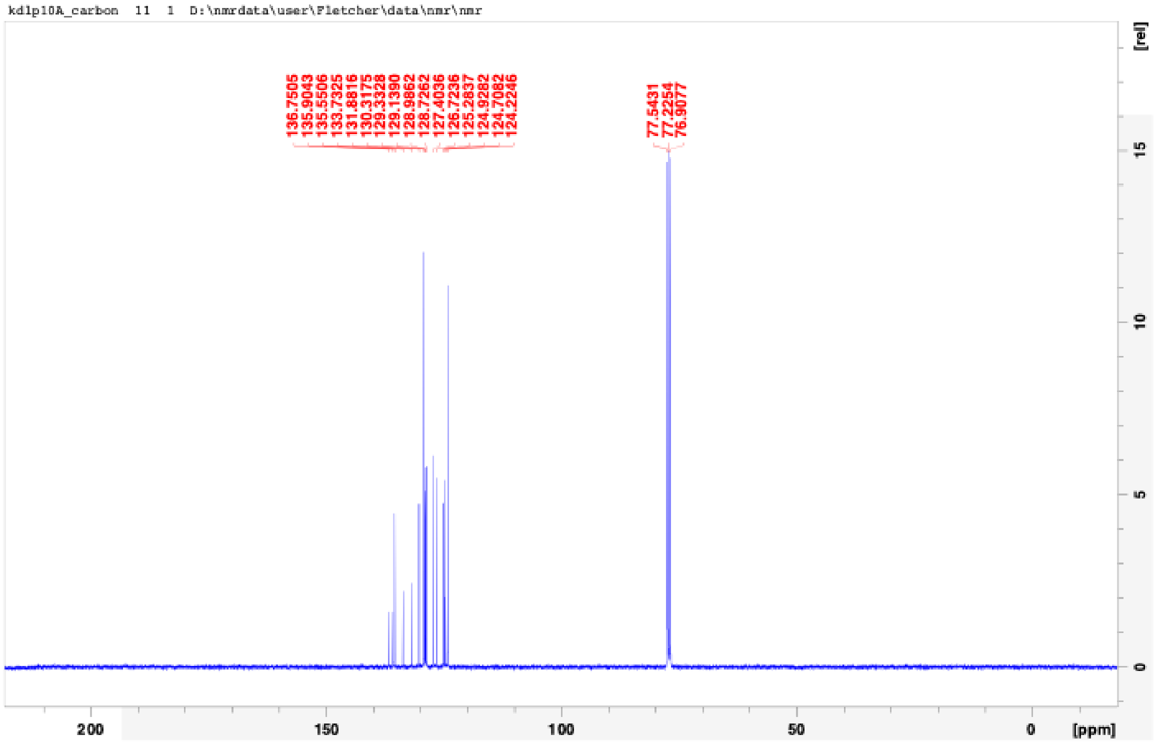

5-(4-Isoquinolinyl)-1-phenyl-1*H*-1,2,3-triazole (**38**)

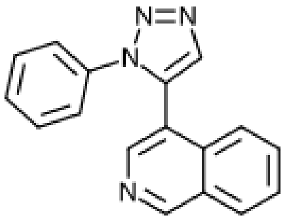

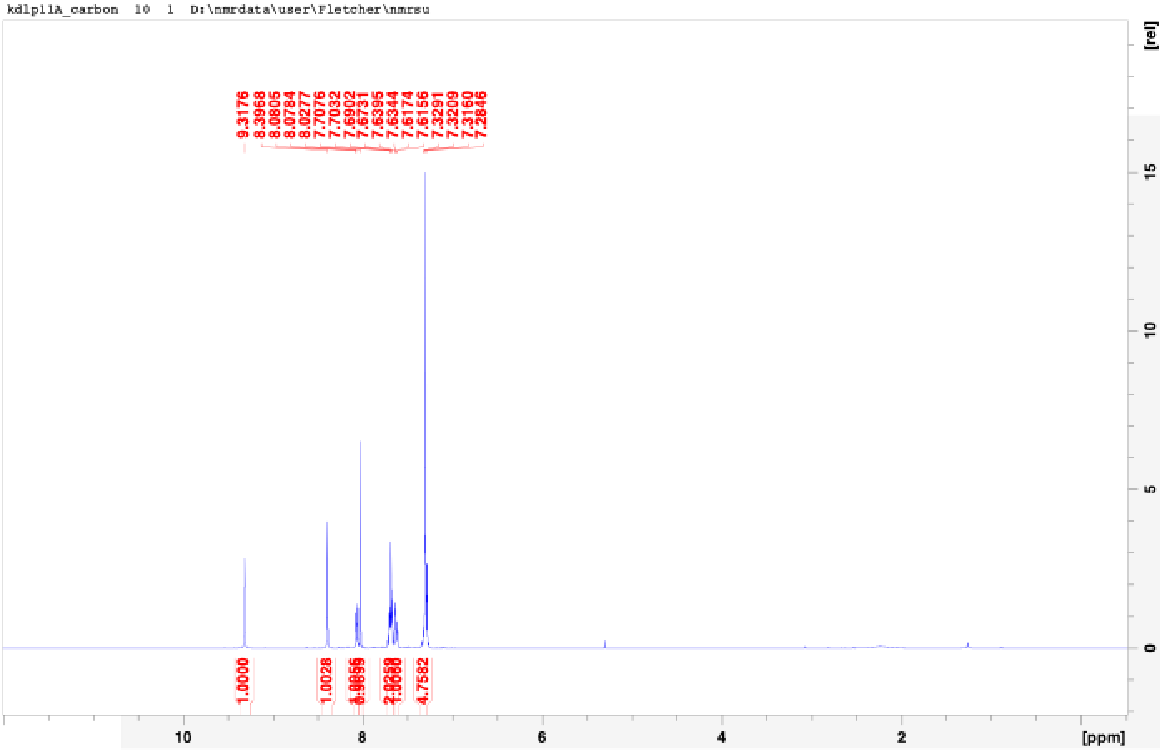

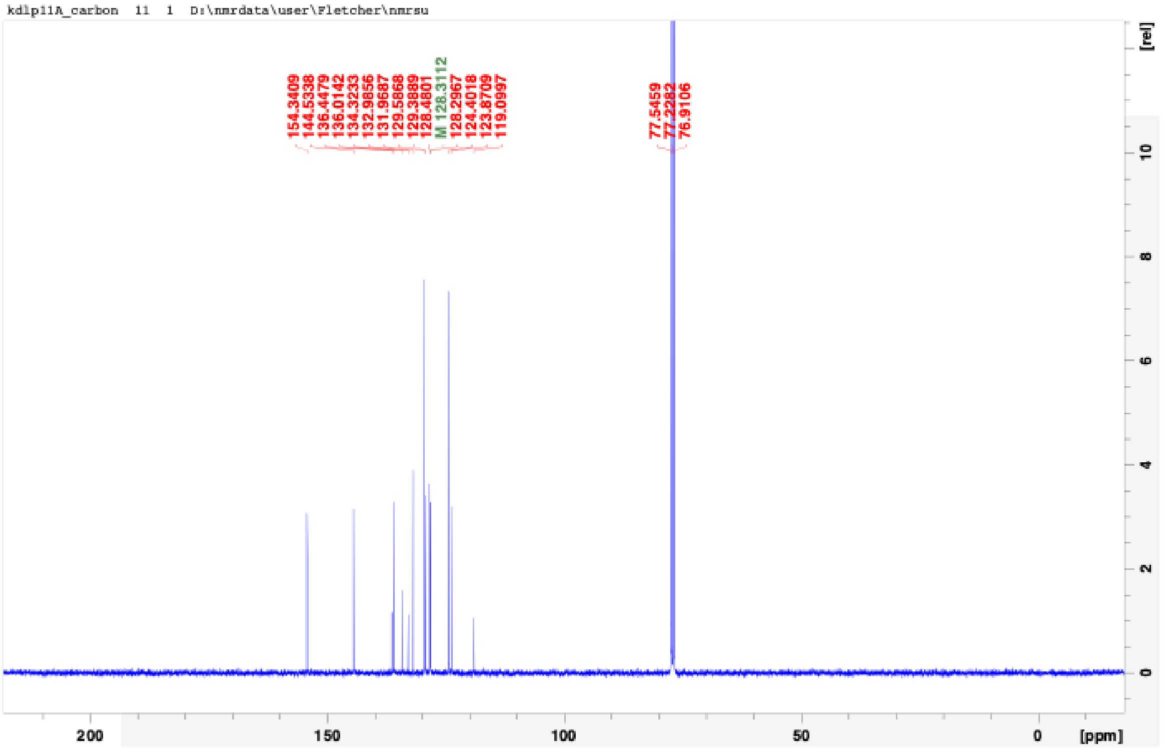

1-Phenyl-5-(4-quinolinyl)-1*H*-1,2,3-triazole (**39**)

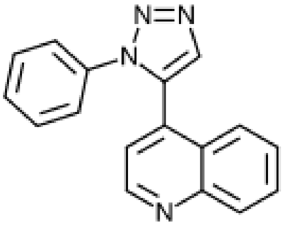

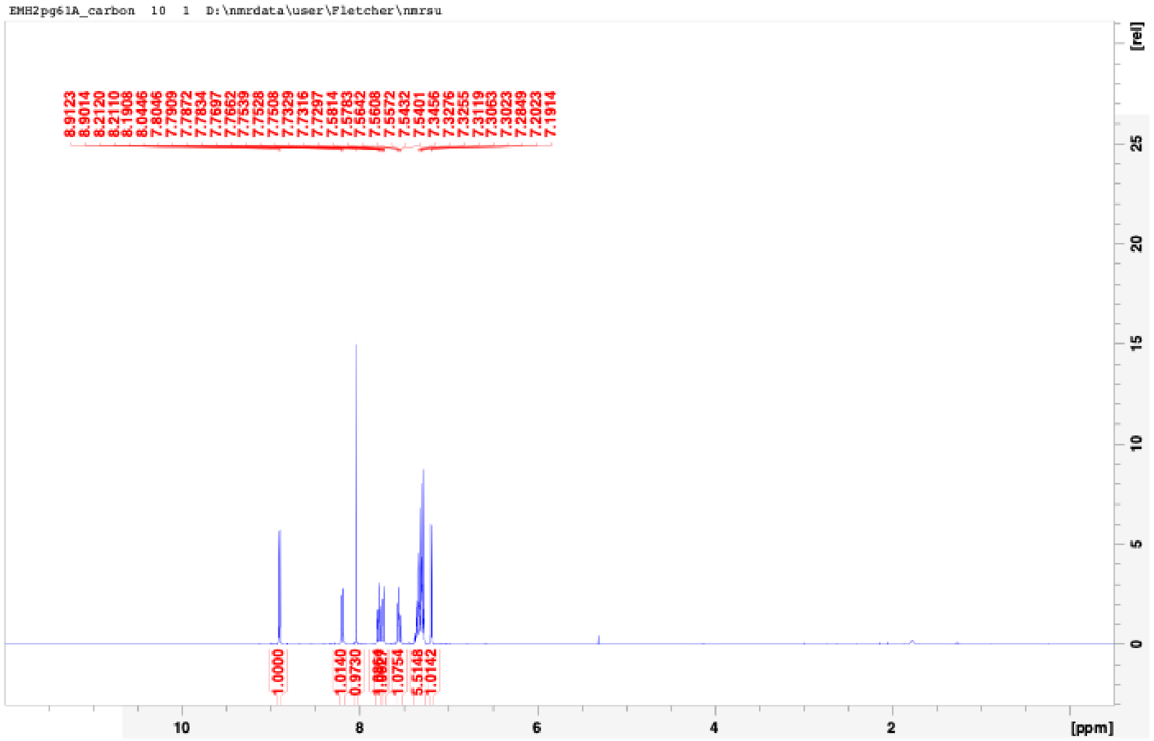

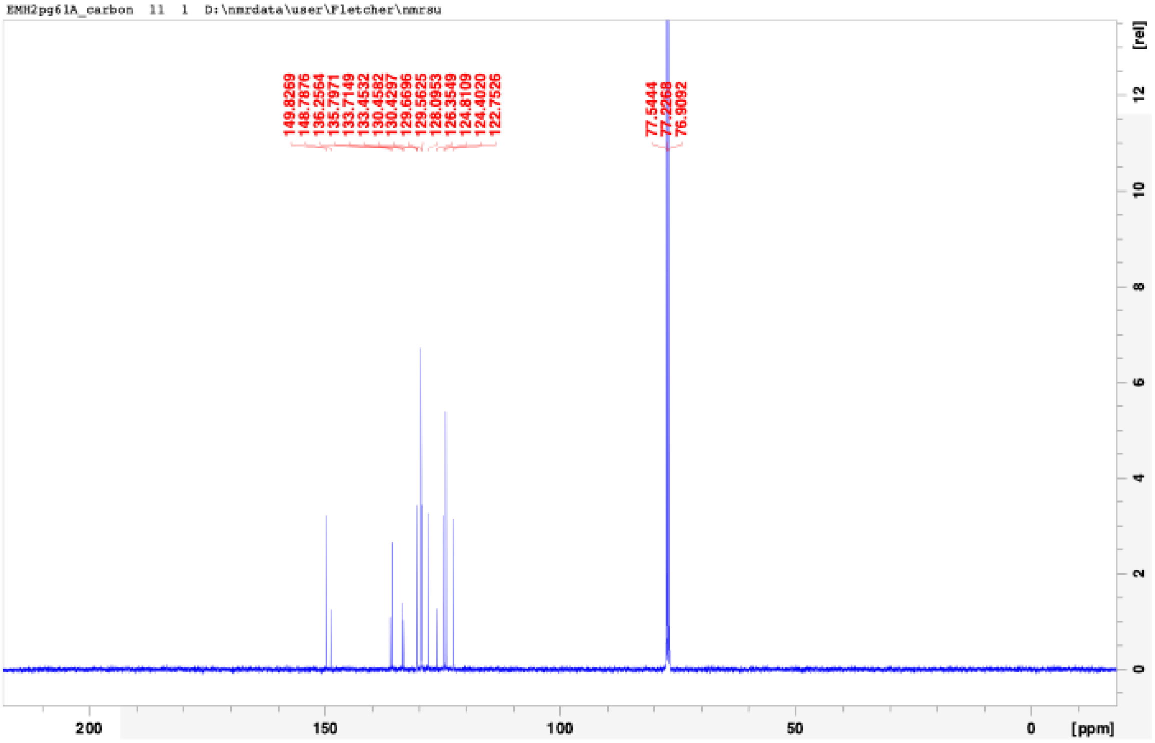

1-Phenyl-5-(5-quinolinyl)-1*H*-1,2,3-triazole (**40**)

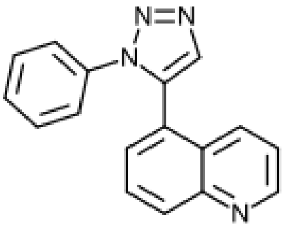

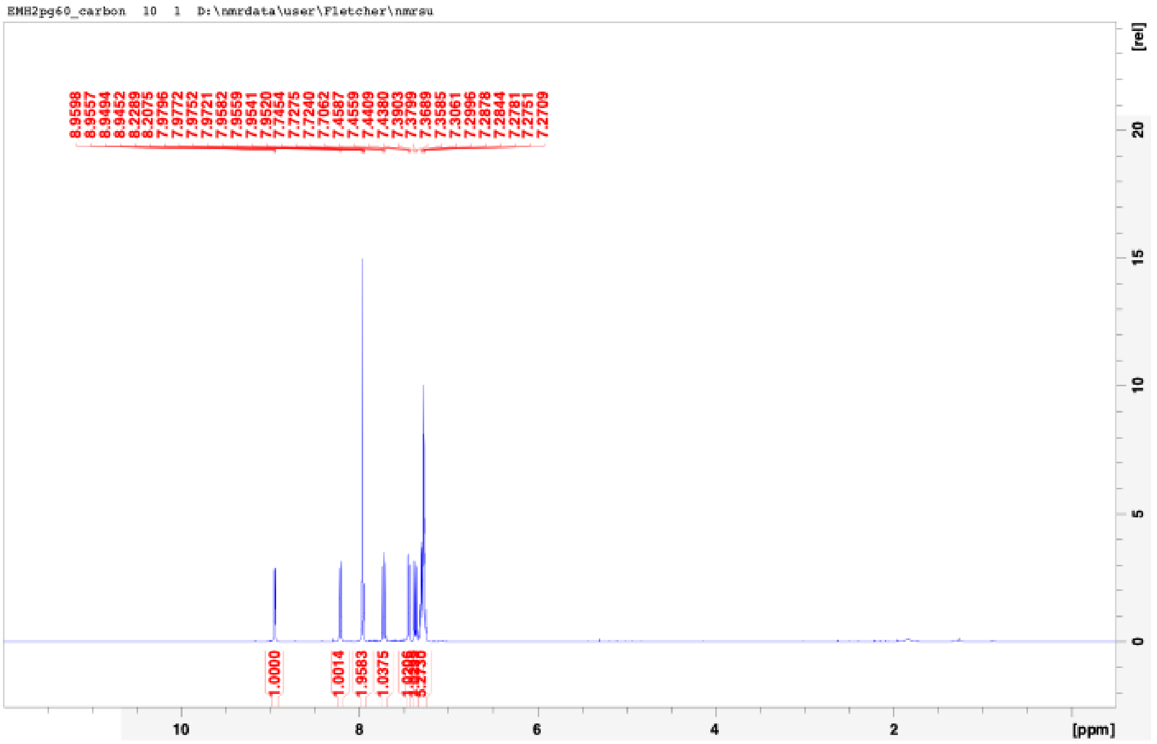

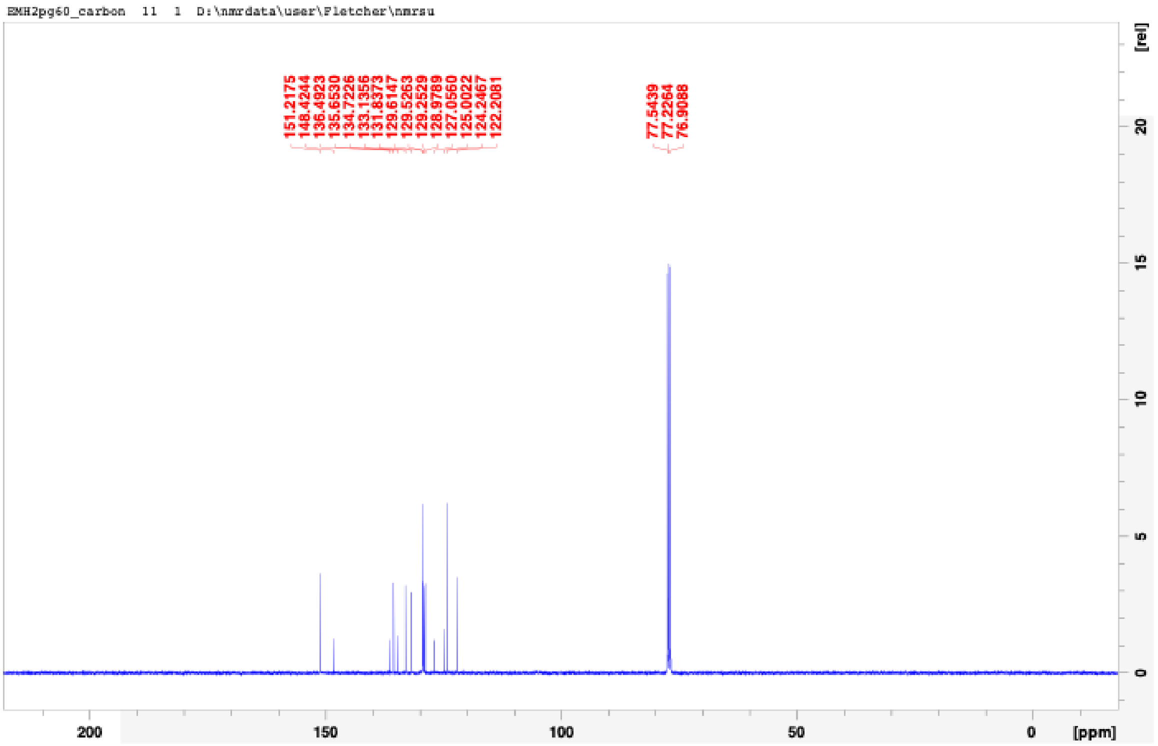

5-(5-Isoquinolinyl)-1-phenyl-1*H*-1,2,3-triazole (**41**)

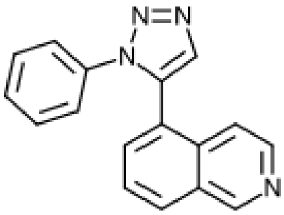

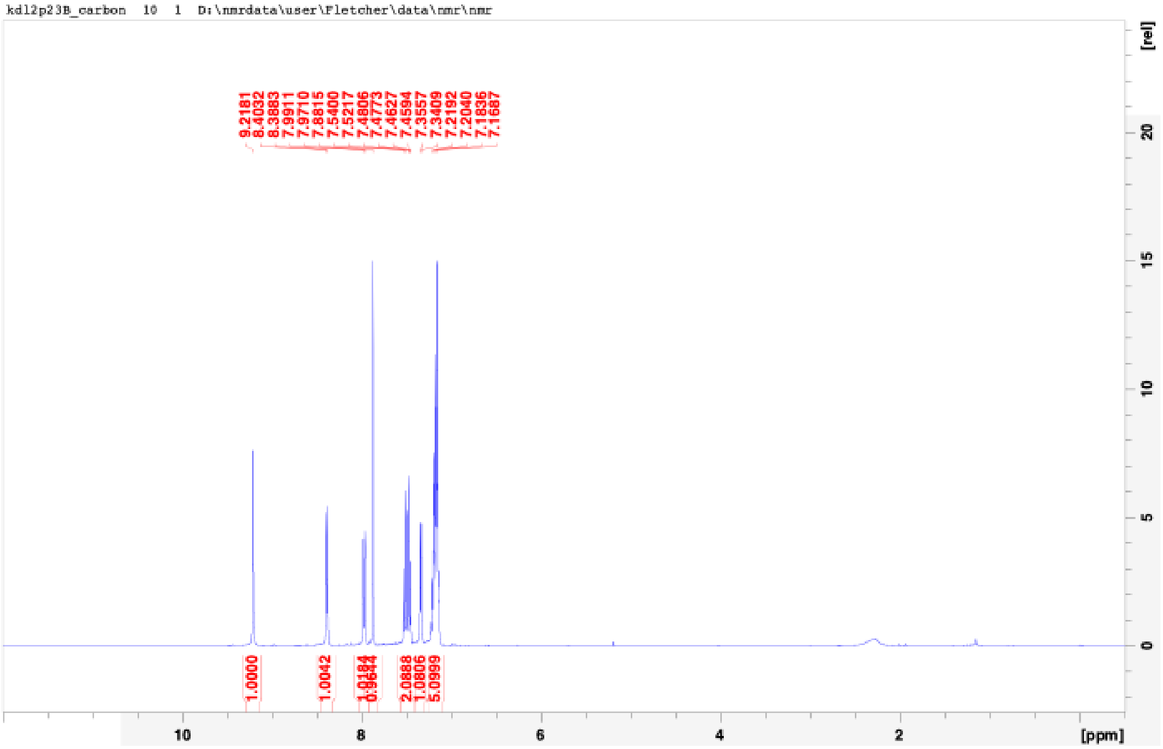

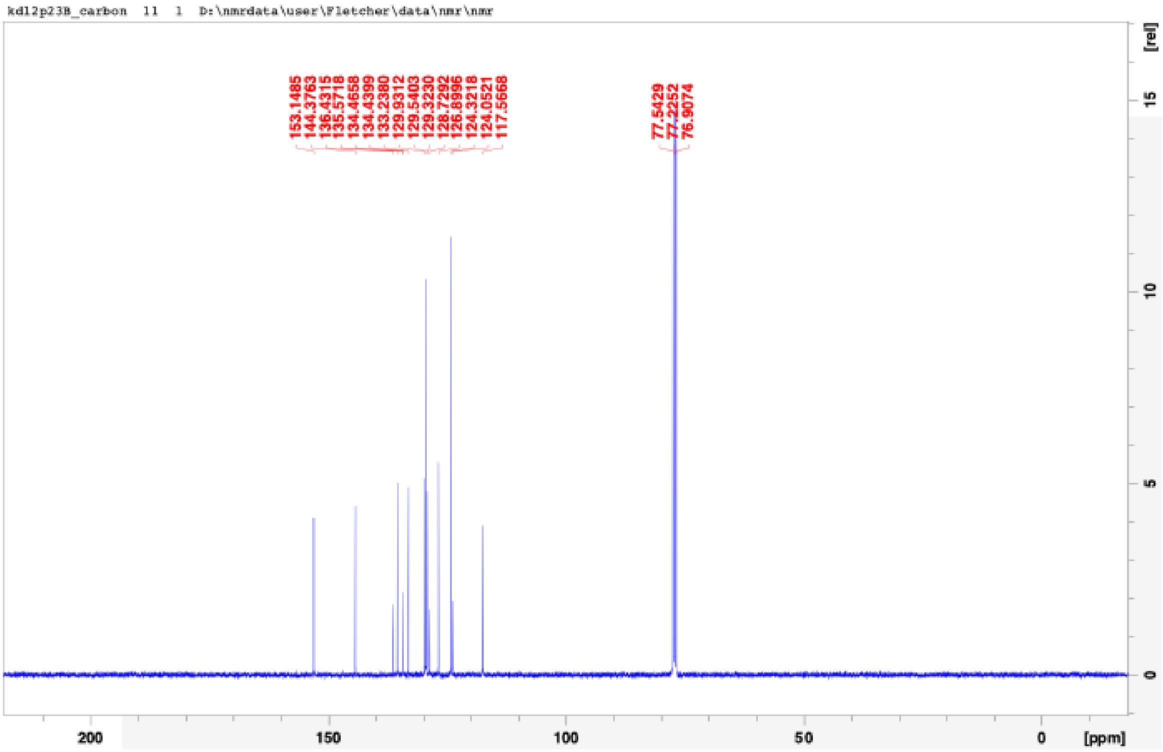

5-(8-Isoquinolinyl)-1-phenyl-1*H*-1,2,3-triazole (**42**)

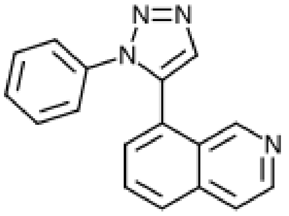

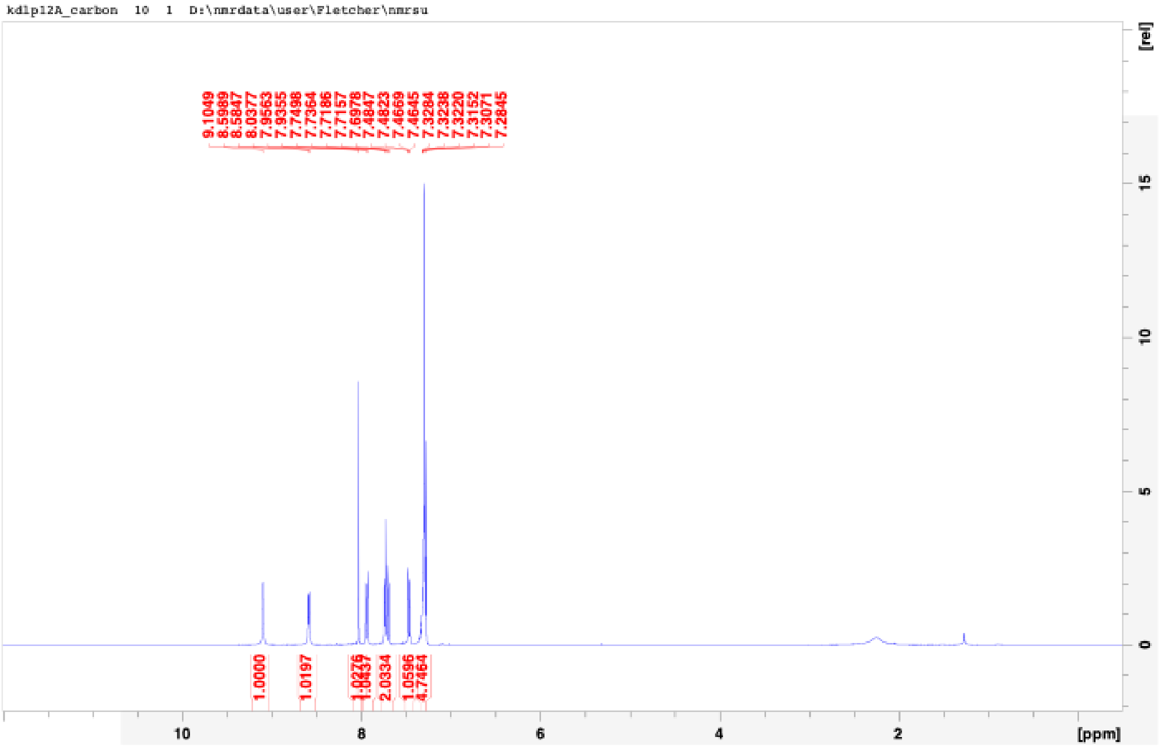

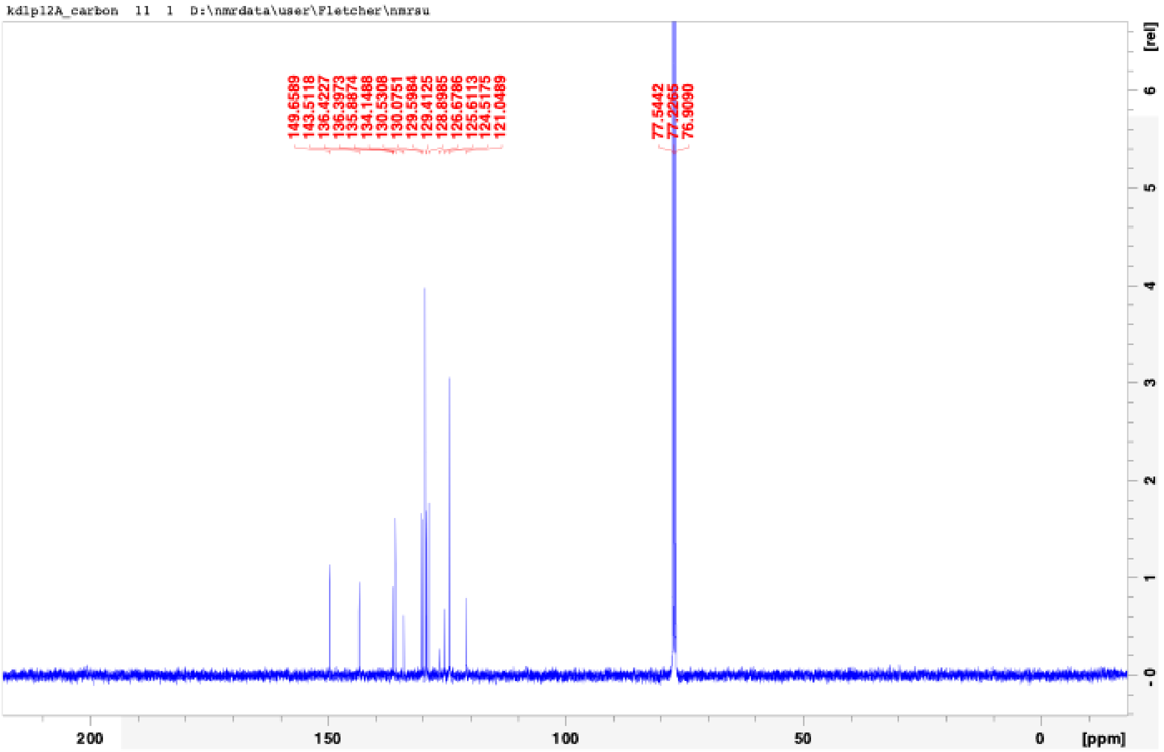

1-(1-Naphthalenyl)-5-phenyl-1*H*-1,2,3-triazole (**43**)

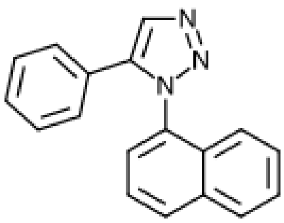

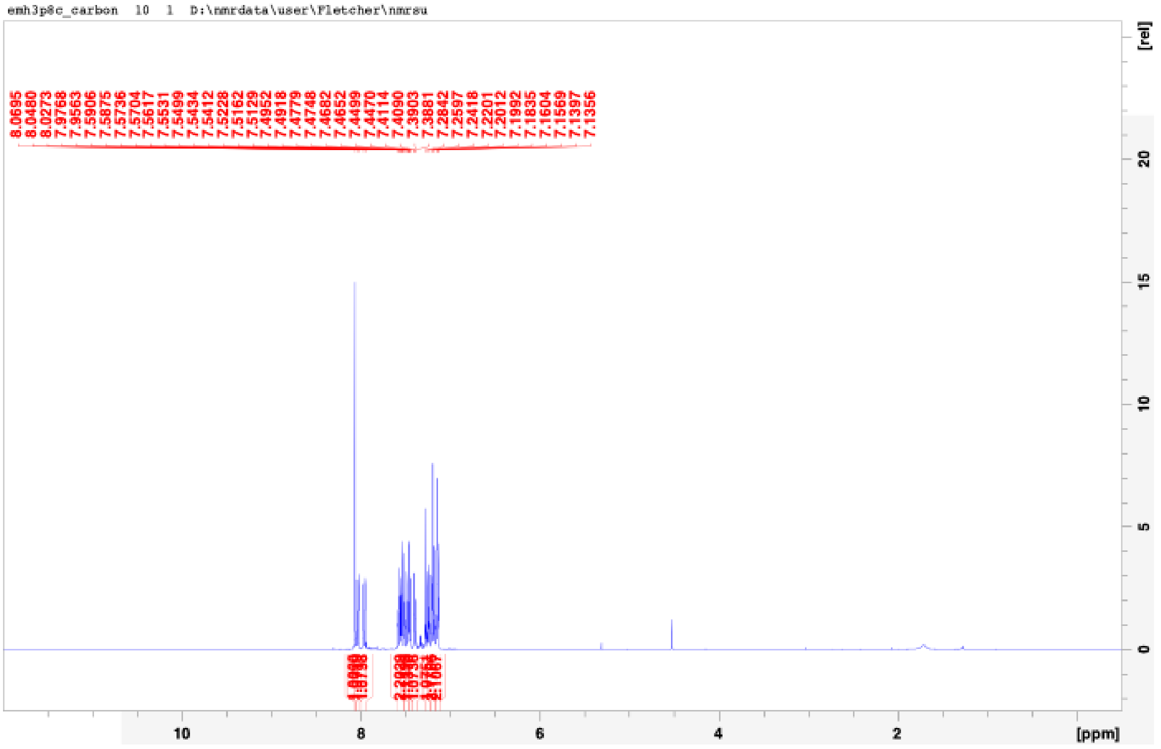

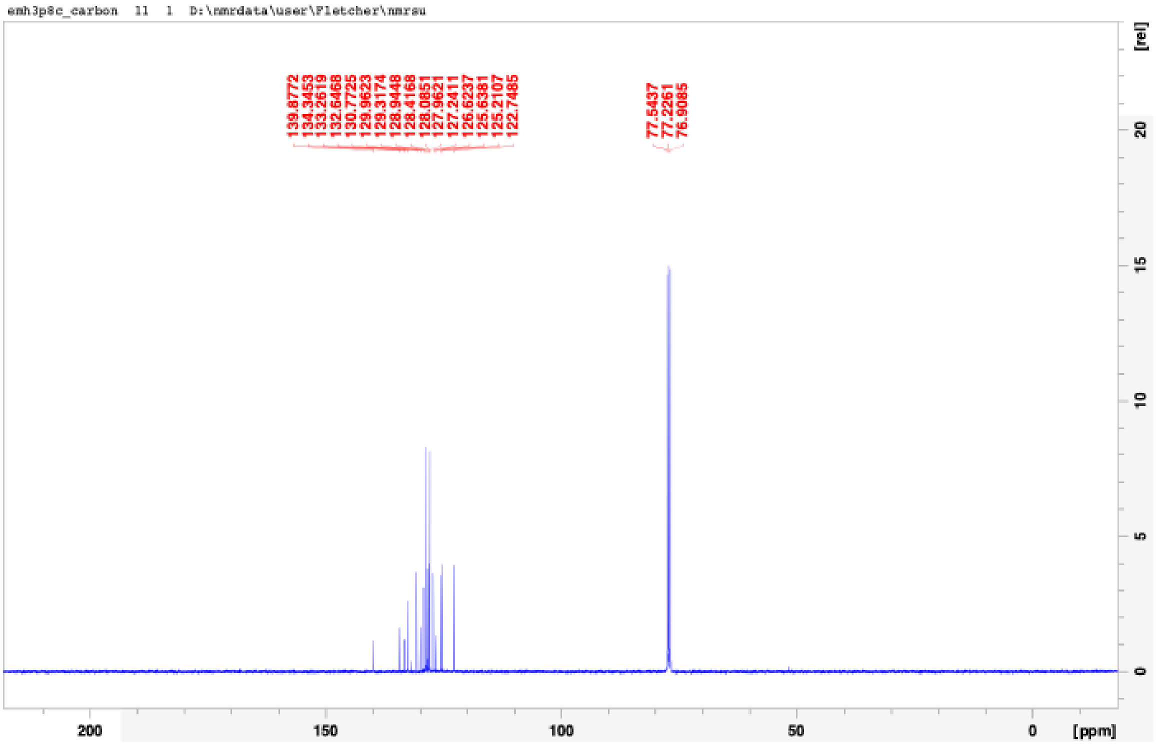

1-(4-Isoquinolinyl)-5-phenyl-1*H*-1,2,3-triazole (**44**)

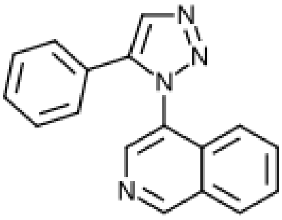

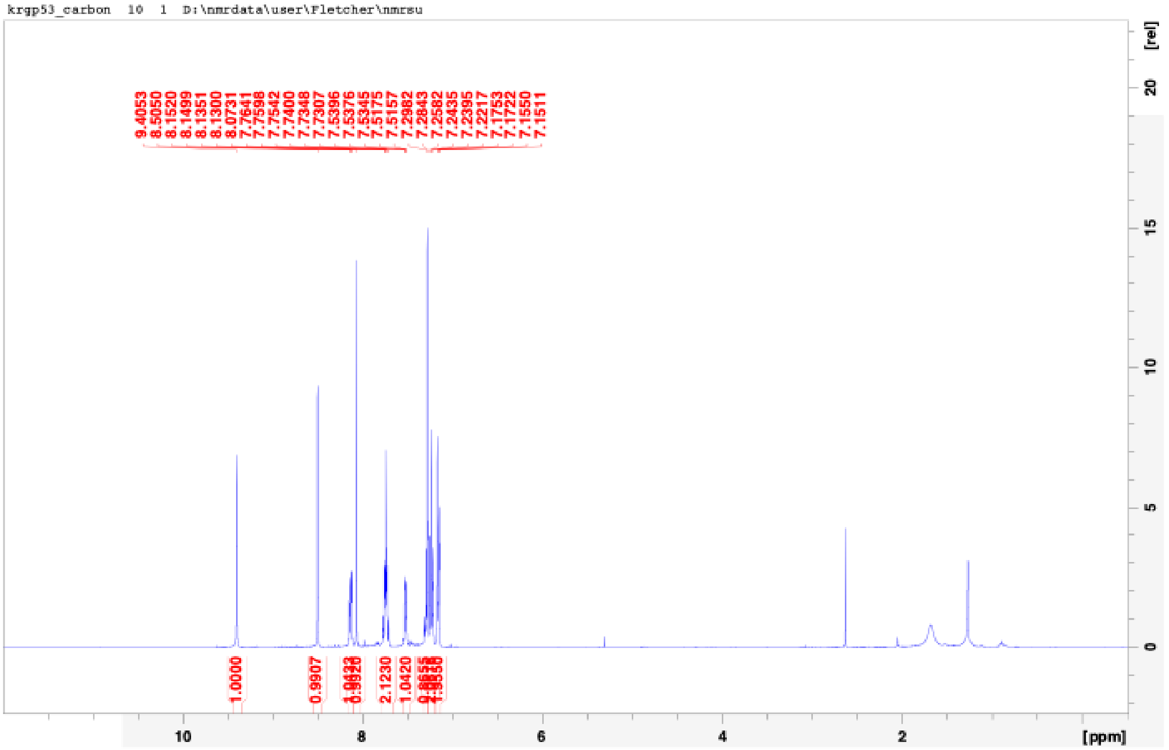

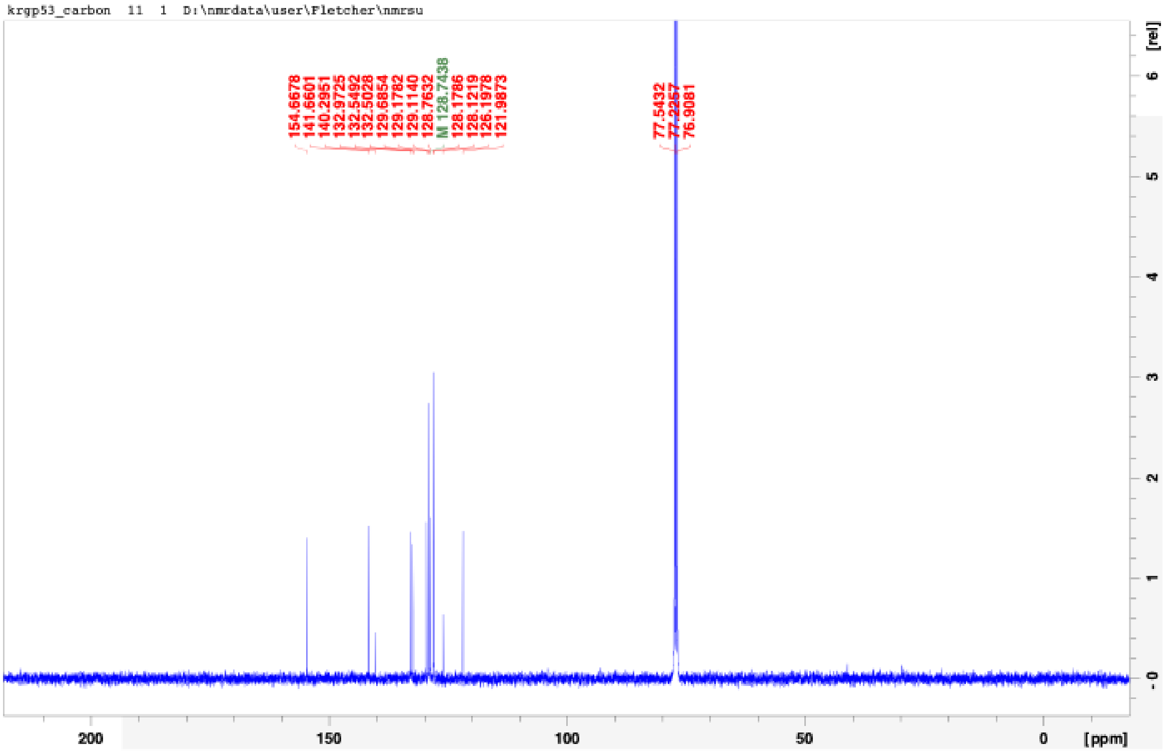

5-Phenyl-1-(4-quinolinyl)-1*H*-1,2,3-triazole (**45**)

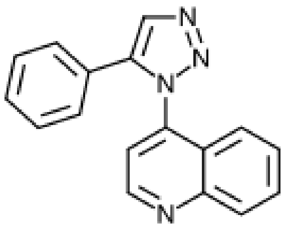

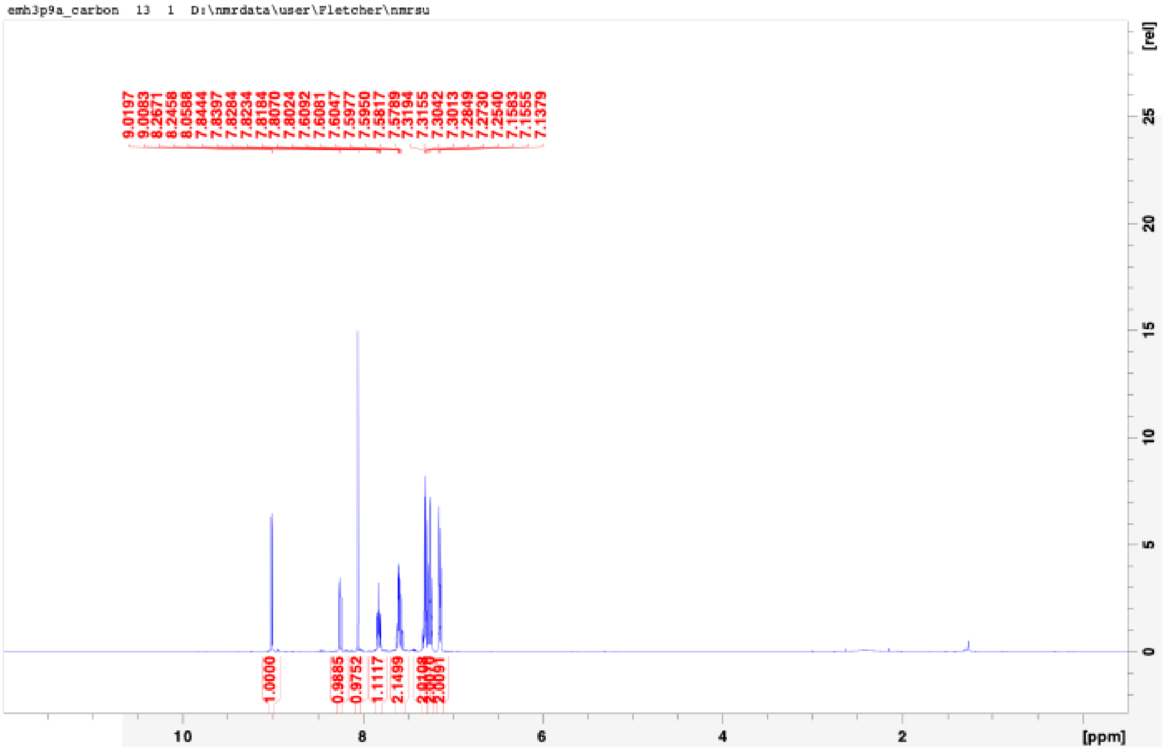

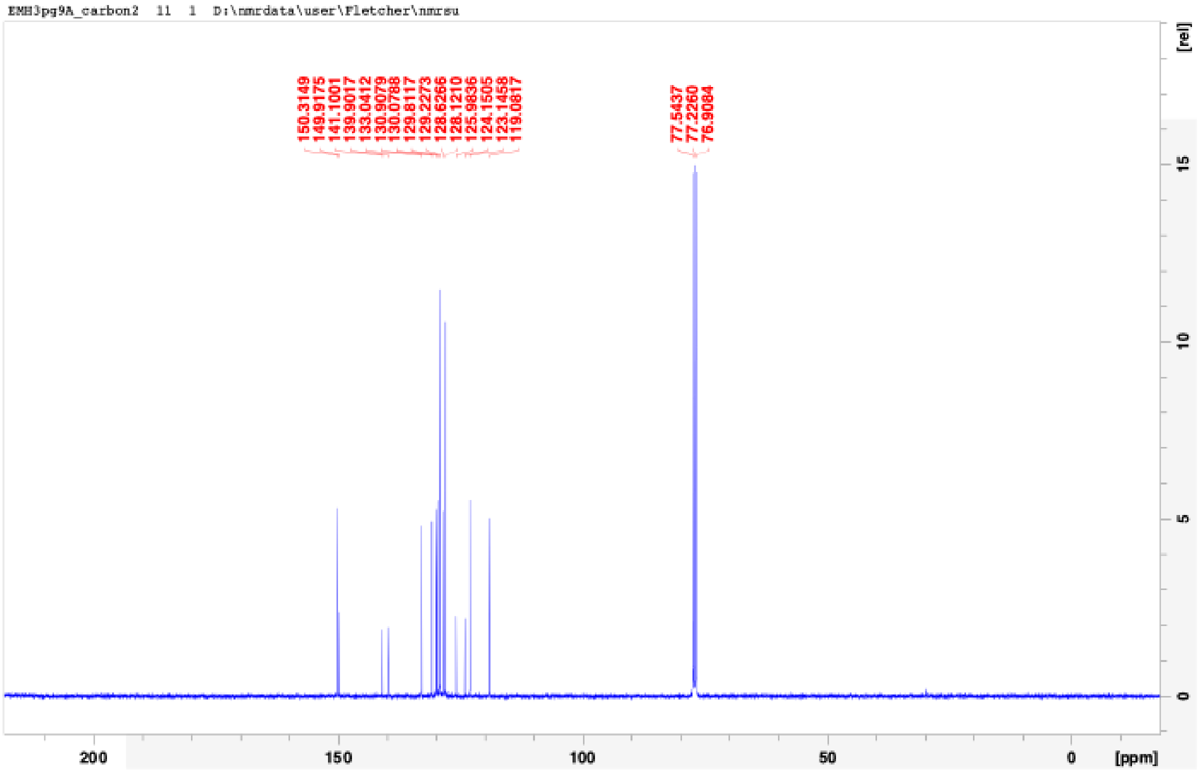

5-Phenyl-1-(5-quinolinyl)-1*H*-1,2,3-triazole (**46**)

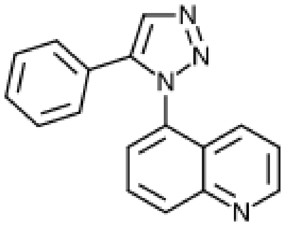

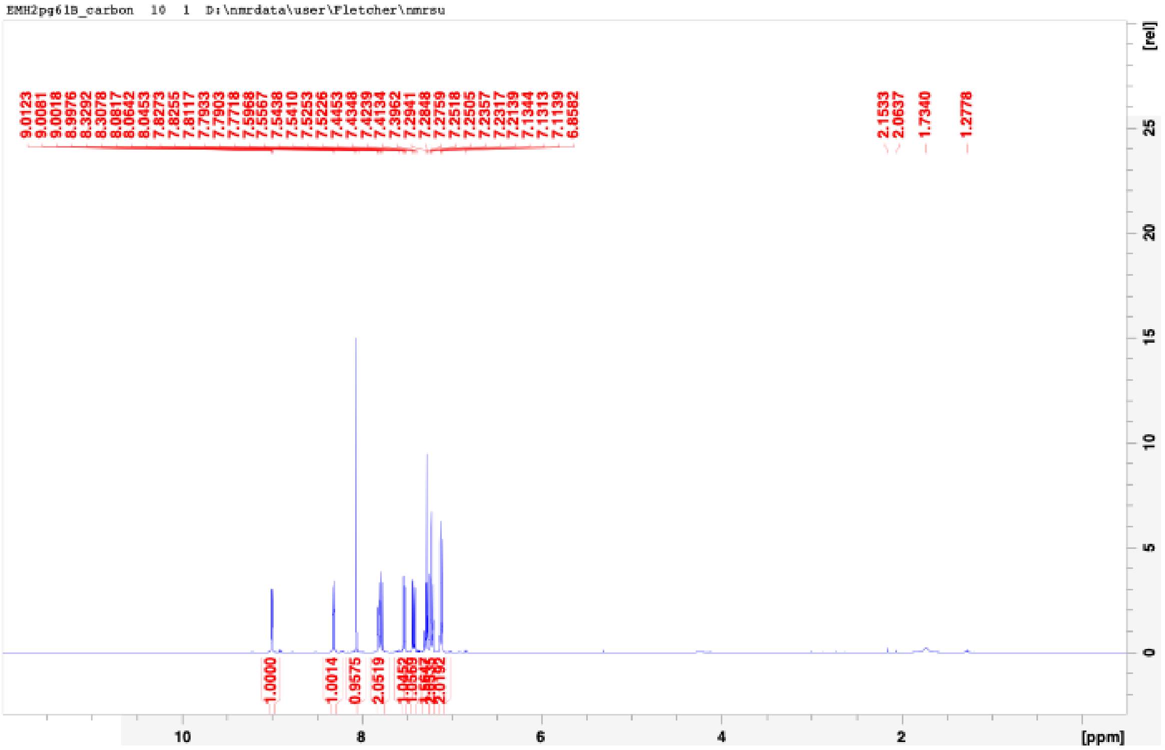

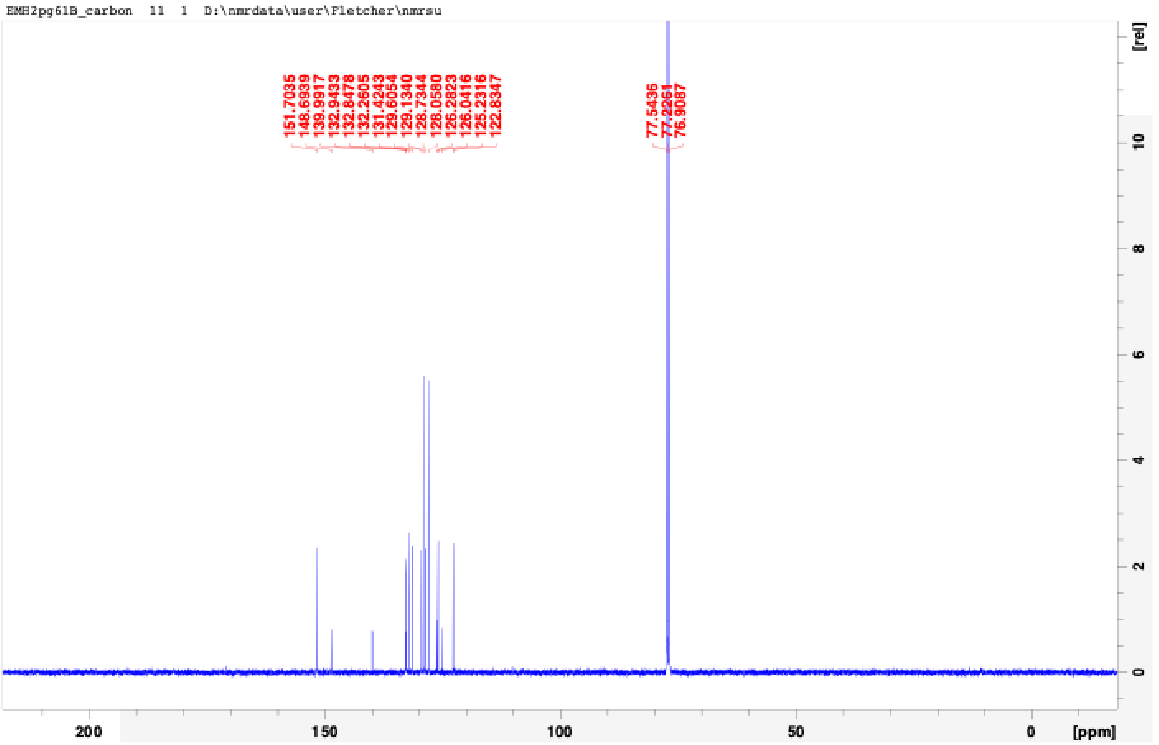

1-(5-Isoquinolinyl)-5-phenyl-1*H*-1,2,3-triazole (**47**)

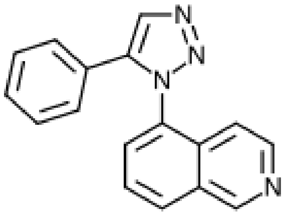

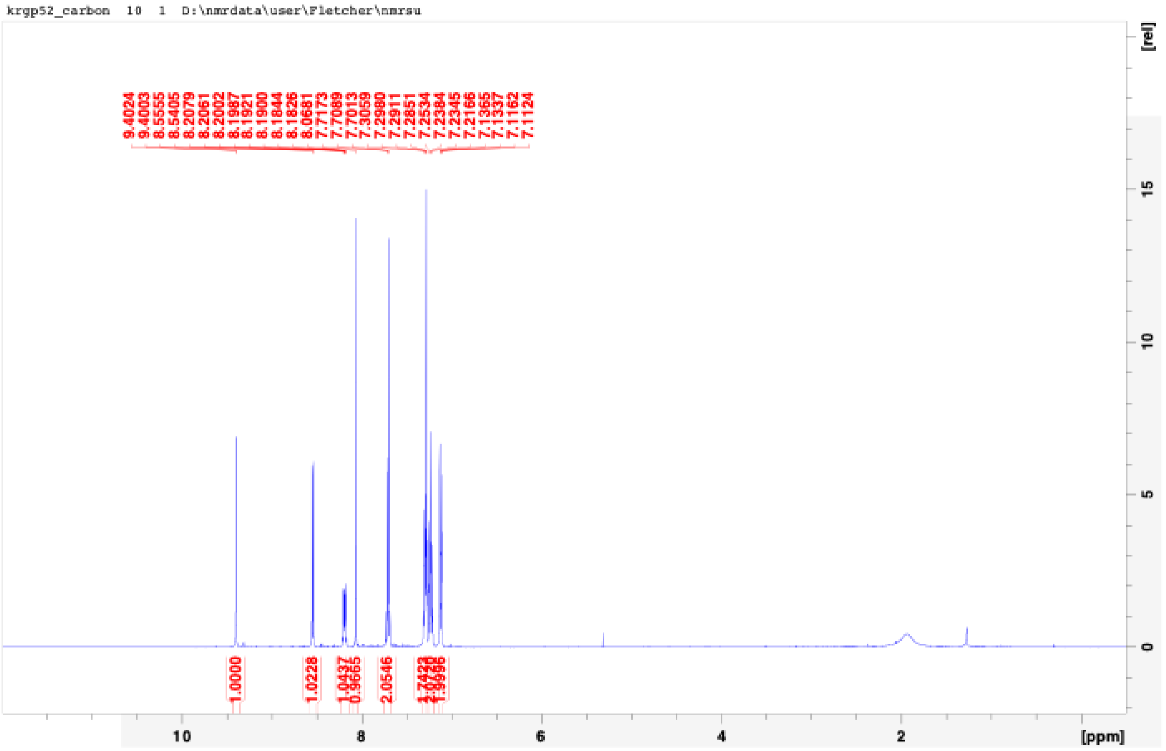

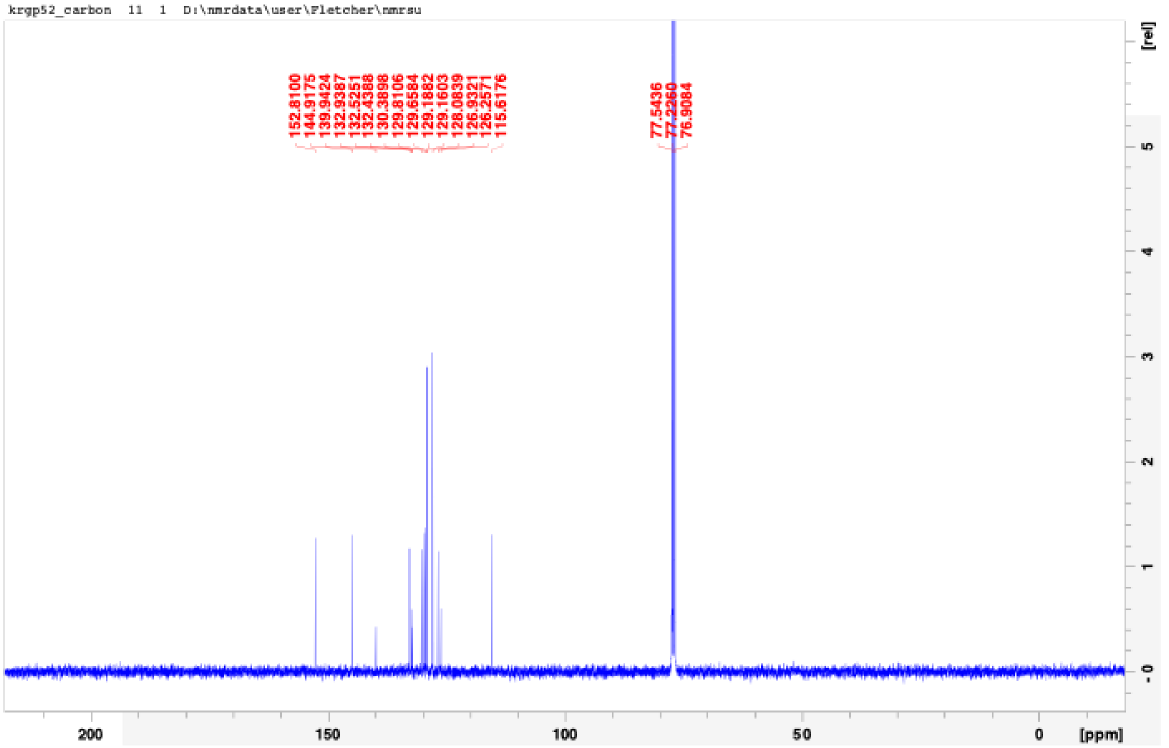

1-(8-Isoquinolinyl)-5-phenyl-1*H*-1,2,3-triazole (**48**)

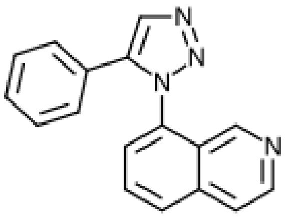

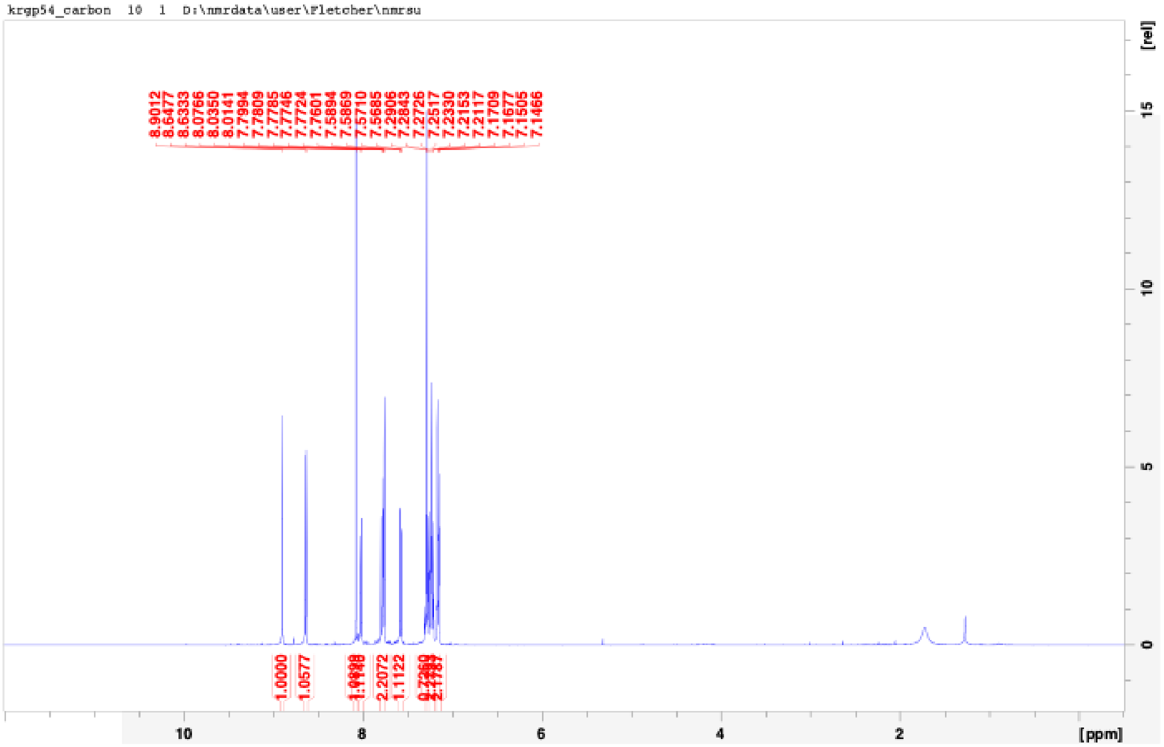

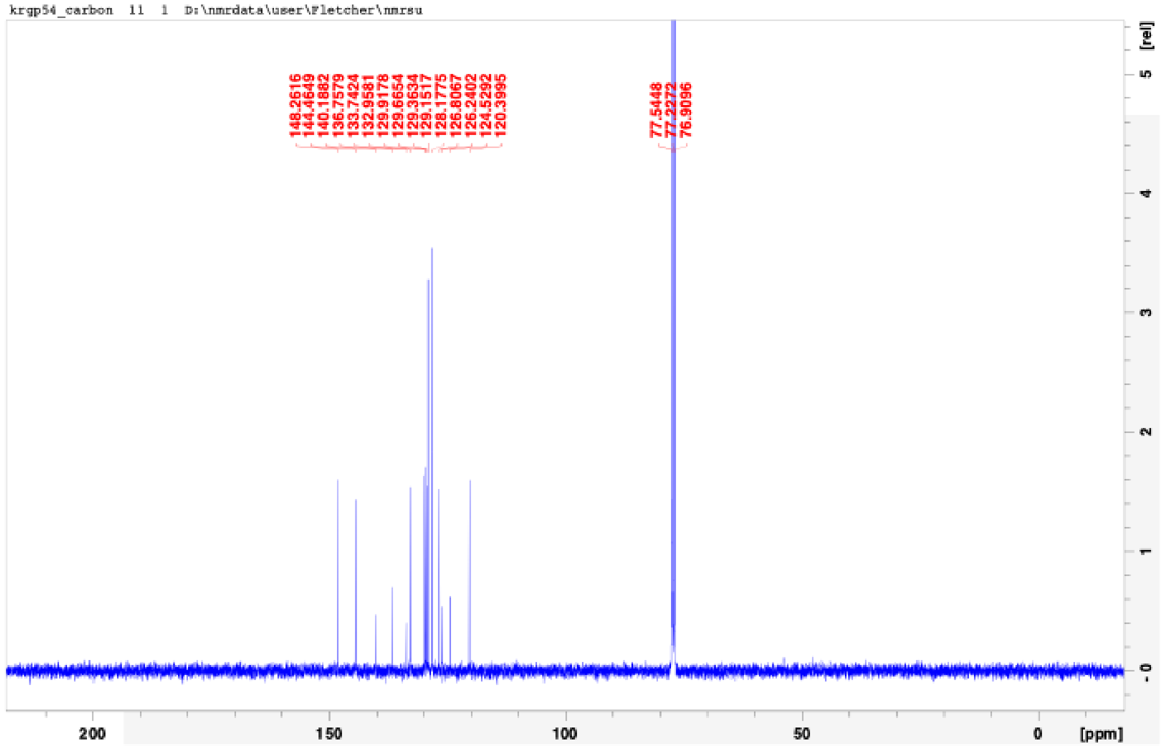

References

(1) Gou, G.; Zhang, Z.; Yuan, B.; Fan, T.; Li, L. *Dyes and Pigments* 2021, *194*, 109642. doi:10.1016/j.dyepig.2021.109642

(2) Xie, Z.; Wu, B.; Liu, Y.; Ren, W.; Tong, L.; Xiang, C.; Wei, A.; Gao, Y.; Zeng, L.; Xie, H.; Tang, W.; Hu, Y. *J Med Chem* 2020, *63*, 1397–1414. doi:10.1021/acs.jmedchem.9b01912

(3) Lei, Y.; Hu, T.; Wu, X.; Wu, Y.; Xiang, H.; Sun, H.; You, Q.; Zhang, X. *Tetrahedron Lett* 2016, *57*, 1100–1103. doi:10.1016/j.tetlet.2016.01.088

(4) Lu, C.; Wang, C.; Jimenez, J. C.; Rheingold, A. L.; Sauvé, G. *ACS Omega* 2020, *5*, 31467–31472. doi:10.1021/acsomega.0c05169

(5) Dutta, U.; Lupton, D. W.; Maiti, D. *Org Lett* 2016, *18*, 860–863. doi:10.1021/acs.orglett.6b00147

(6) Walther, A.; Regeni, I.; Holstein, J. J.; Clever, G. H. *J Am Chem Soc* 2023, *145*, 25365–25371. doi:10.1021/jacs.3c09295

(7) Hu, M.; Li, J.; Q. Yao, S. *Org Lett* 2008, *10*, 5529–5531. doi:10.1021/ol802286g

(8) Kirsch, P.; Stein, S. C.; Berwanger, A.; Rinkes, J.; Jakob, V.; Schulz, T. F.; Empting, M. *Eur J Med Chem* 2020, *202*. doi:10.1016/j.ejmech.2020.112525

(9) Mendes, E.; Cadoni, E.; Carneiro, F.; Afonso, M. B.; Brito, H.; Lavrado, J.; dos Santos, D. J. V. A.; Vítor, J. B.; Neidle, S.; Rodrigues, C. M. P.; Paulo, A. *ChemMedChem* 2019, *14*, 1325–1328. doi:10.1002/cmdc.201900243

(10) Chen, D.; Osipyan, A.; Adriana, J.; Kader, M.; Gureev, M.; Knol, C. W. J.; Sigmund, M. C.; Xiao, Z.; van der Wouden, P. E.; Cool, R. H.; Poelarends, G. J.; Dekker, F. J. *J Med Chem* 2023, *66*, 8767–8781. doi:10.1021/acs.jmedchem.3c00397

(11) Peng, X.; Wang, Q.; Mishra, Y.; Xu, J.; Reichert, D. E.; Malik, M.; Taylor, M.; Luedtke, R. R.; Mach, R. H. *Bioorg Med Chem Lett* 2015, *25*, 519–523. doi:10.1016/j.bmcl.2014.12.023

(12) Kwok, S. W.; Fotsing, J. R.; Fraser, R. J.; Rodionov, V. O.; Fokin, V. V. *Org Lett* 2010, *12*, 4217–4219. doi:10.1021/ol101568d

(13) Riss, A.; Wickenburg, S.; Gorman, P.; Tan, L. Z.; Tsai, H.-Z.; de Oteyza, D. G.; Chen, Y.-C.; Bradley, A. J.; Ugeda, M. M.; Etkin, G.; Louie, S. G.; Fischer, F. R.; Crommie, M. F. *Nano Lett* 2014, *14*, 2251–2255. doi:10.1021/nl403791q

(14) CLSI. *Methods for Dilution Antimicrobial Susceptibility Tests for Bacteria That Grow Aerobically.* 11th ed. CLSI standard M07. Wayne, PA: Clinical and Laboratory Standards Institute, 2017.

(15) CLSI *Reference Method for Broth Dilution Antifungal Susceptibility Testing of Yeasts.* 4th ed. CLSI standard M27. Wayne, PA: Clinical and Laboratory Standards Institute, 2017.
